# Conceptual framework for increasing legitimacy and trust of sustainability governance

**DOI:** 10.1186/s13705-021-00280-x

**Published:** 2021-03-18

**Authors:** Inge Stupak, Maha Mansoor, C. Tattersall Smith

**Affiliations:** 1 Department of Geosciences and Natural Resource Management, University of Copenhagen, Rolighedsvej 23, 1958 Frederiksberg C, Denmark; 2 University of Toronto, Toronto, Canada

**Keywords:** Bioeconomy, Bioenergy, Certification, Adaptive management, Governance, Legitimacy, Sustainability, Social license to operate, Policy, Trust

## Abstract

While the quantity of sustainability governance initiatives and systems has increased dramatically, crises persist over whether specific governance systems can be trusted as legitimate regulators of the sustainability of economic activities. This paper focuses on conceptual tools to improve our understanding of these crises as well as the facilitating factors and barriers for sustainability governance to play a role in transitioning to profoundly more sustainable societies than those that currently exist. Bioenergy is used throughout the paper as an example to aid contextually in understanding the theoretical and abstract arguments. We first define eight premises upon which our argumentation is developed. We then define sustainability, sustainability transition, legitimacy, and trust as a premise for obtaining effectiveness in communication and minimising risks associated with misunderstanding key terms. We proceed to examine the literature on “good governance” in order to reflect upon what defines "good sustainability governance" and what makes governance systems successful in achieving their goals. We propose input, output, and throughput legitimacy as three principles constituting “good” sustainability governance and propose associated open-ended criteria as a basis for developing operational standards for assessing the quality of a sustainability governance system or complex. As sustainability governance systems must develop to remain relevant, we also suggest an adaptive governance model, where continuous re-evaluation of the sustainability governance system design supports the system in remaining “good” in conditions that are complex and dynamic. Finally, we pull from the literature in a broad range of sciences to propose a conceptual “governance research framework” that aims to facilitate an integrated understanding of how the design of sustainability governance systems influences the legitimacy and trust granted to them by relevant actors. The framework is intended to enhance the adaptive features of sustainability governance systems so as to allow the identification of the causes of existing and emerging sustainability governance crises and finding solutions to them. Knowledge generated from its use may form a basis for providing policy recommendations on how to practically solve complex legitimacy and trust crises related to sustainability governance.

**Supplementary Information:**

The online version contains supplementary material available at 10.1186/s13705-021-00280-x.

## Introduction

Since the 2010s, sustainability governance has emerged in the literature as distinct from the well-established discipline of “environmental governance”. There is a diversity of approaches to sustainability governance, but common to all is that they assume a set of goals based on an understanding of sustainability. Yet, both sustainability and environmental governance differ in nature from many other policy areas because of the complexity of the goals and the uncertainty or ambivalence in which they are embedded [1, 2]. While sustainability governance has proliferated as a field of research, great concerns remain about unsustainable practices in several economic sectors. Existing sustainability governance systems must therefore continue to develop, adjust and improve to meet continuously identified challenges and concerns to maintain its legitimacy and people’s trust in its usefulness as a means of making progress towards more sustainable societies.

A broad range of research is being conducted to inform decisions about the design and development of specific sustainability governance systems within the disciplines of political, natural, economic and social sciences, as evident, for example, from this collection of articles in Energy, Sustainability and Society. Common across all these scientific disciplines is the pursuit of reducing complex questions to simpler ones, which lend themselves to scrutiny in the pursuit of rigorous scientific analysis. However, interdisciplinary collaboration and integration is necessary to better understand broader questions, such as how the design of sustainability governance systems influence the on-the-ground impacts of the activities they regulate, as well as their achieved legitimacy and trust that they lead to sustainable outcomes. The reason is the complex and multifaceted nature of sustainability [3], which encompasses, at minimum, ecological, social, and economic issues at local to global scales.

Building on existing efforts made within and across a wide range of scientific disciplines, we aim to set an even wider context by developing a framework that conceptualises how each discipline may contribute to answer the following question:

How is the design of sustainability governance systems linked to people’s granting of legitimacy to the system and trust that the system leads to more sustainable outcomes for the regulated economic activity; how do these relationships depend on various institutional, economic, social and environmental factors?

In order to address this question, this paper:

- introduces bioenergy as an example to illustrate conceptual and theoretical argumentation, and utilises this example throughout the paper;
- sets the premises underlying the argumentation in the paper, including defining and explaining key definitions, terminology and concepts;
- reviews existing “good governance” standards as a basis for suggesting how “good sustainability governance” can be defined as a concept, and how it can be translated for practical use in assessments of the quality of a sustainability governance system;
- suggests a conceptual governance research framework for the structured integration of knowledge across a range of scientific disciplines and as an approach to examine and answer the broader question given above; and
- discusses the broader context within which we seek to increase the legitimacy and trust granted to, for example, bioenergy sustainability governance systems.

This paper can be viewed as a contribution to the field of sustainability transition, which takes a systems approach to gaining insights on how to make progress towards more sustainable societies [4], using the bioenergy sector as an example throughout. We hope the contribution may form a starting point for the integration of existing research into informed policy-decision and thus become a small, but useful, piece in the larger puzzle of profound sustainability transitions.

## The example of bioenergy

This section provides the context for the examples of bioenergy used throughout the paper. It describes the development of bioenergy as a significant source of renewable energy around the world, outlines the crises that have emerged as some actors in society reject it as a sustainable source of renewable energy, and explains how the sustainability governance systems have not yet been able to suitably satisfy those actors.

Bioenergy utilises biomass to produce energy in all sectors: biomass and biogas is used for the production of electricity and heat while liquid biofuels and biogas is used as a replacement for gasoline and diesel in the transportation sector. Although there are a wide variety of feedstocks, bioenergy mainly come from agriculture, forestry, the residual streams from their related industries, and the waste management sectors. The International Energy Agency (IEA) reports that bioenergy is the main source of renewable energy, contributing 12.8% (46.4 EJ) of the total global energy consumption, with a distribution of 59% and 41% to traditional subsistence use and modern larger scale bioenergy production, respectively, in 2016 [5]. The IEA also reports that global transportation biofuel production increased by 10 billion litres to reach a record of 154 billion litres in 2018, with forecasts that production will increase a further 25% by 2024 [6].

### Bioenergy policy and sector development

Following a long period of nearly total fossil fuel dependency after World War II, countries turned to bioenergy as a renewable energy source in response to the oil crisis of 1973 [7]. Some Nordic countries and Austria, for example, began to rely on forest and agriculture-based bioenergy as an alternative to oil in domestic heat and electricity production [8–10]. Brazil responded similarly to the oil crisis with the government implementing supportive policies to establish sugarcane-based bioethanol as a substitute for fossil fuels in the transportation sector [11]. The shift toward an increasing use of bioenergy continued in the succeeding decades, to seek energy security and rural development. By the 1990s, though, climate change had moved to the forefront of global concern [12] and nations looked to bioenergy as a means to accomplishing the greenhouse gas (GHG) emission reduction targets pledged in international agreements, for example, the Kyoto Protocol signed in 1997 [13] and more recently in the Nationally Determined Contributions under the Paris Agreement signed in 2015 [14].

As a result of the international push to lower GHG emissions, since the 2000s, countries have increasingly implemented policy supports for renewable energy. By 2017, 128, 70 and 29 countries enacted policies that financially support the use of renewable energy in the power, transportation and the heat and cooling sectors, respectively [5]. Through such supporting policies, the European Union (EU) has become the largest consumer of modern bioenergy [5, 14]. Modern bioenergy does not include the traditional uses of biomass, such as open fires or cook stoves, which are most often used in developing countries.

In EU climate and energy policies dated 2007 [15] and 2014 [16], targets were set for GHG emission reductions, renewable energy, and improved energy efficiency for 2020 and 2030, respectively. Its 2018 long-term strategy [17] lays out the pathway to a low carbon economy in 2050 as part of EU’s commitment made in the Paris Agreement. The targets are enforced through the EU Emission Trading System (ETS), EU directives and EU regulations that are implemented in member states through national legislation.

The EU policies set an EU-wide target for GHG emission reductions at 30% and 40% below 1990-levels by 2020 and 2030, respectively. The corresponding targets for renewable energy consumption are 20% and 37% of the final energy consumption. The 2020 renewable energy targets are implemented through the Renewable Energy Directive from 2009 (RED I) [18], which will be repealed and replaced with the revised Renewable Energy Directive (RED II) [19] in 2021, to implement the targets for 2030. Both directives also set national renewable energy targets for each member state, with the intention that it will allow the EU to meet its overall renewable targets.

Related to transportation fuels, a minimum target of 10% renewable use for each member state was set by 2020 and 14% by 2030. However, concerns for indirect Land Use Changes (iLUC) led to the introduction of a cap that limits the contribution of transportation biofuels based on food and feed crops to maximum of 7% by 2020, but not more than 1% point above a member state’s share of such fuels for transportation in 2020. Fuels produced from feedstocks defined with “high indirect land-use change-risk” must also be limited to 2019 consumption levels, unless they are specifically certified to be “low indirect land-use change-risk” biofuels. In any case, the use of these high-risk fuels must gradually be phased out to 0% by the end of 2030 [19, 20].

About 80% of the globally produced transportation biofuels are consumed by the EU, Brazil and United States of America (US) [5]. In 2018, the US was the world’s largest producer and user of transportation biofuels followed by Brazil. These record levels of production and consumption were largely a result of supportive policies beginning in 2005, when the US introduced the Renewable Fuels Standard (RFS) under the Energy Policy Act [21]. The RFS program set a minimum blending target of 7.5 billion gallons (28.4 billion liters) of bioethanol consumption by 2012 [22, 23]. The RFS program was expanded in 2007 under the Energy Independence and Security Act [24] to increase the targets and apply minimum thresholds for life cycle GHG performance [23]. In Brazil, the new Biofuels National Policy (RenovaBio) program was introduced in 2018 through legislation with the overall aim to reduce GHG emissions and meet commitments to the Paris Agreement [25]. It also includes mechanisms that encourage companies to follow rules against deforestation caused by agricultural expansion and to generally reduce emissions from production [26].

Outside of Europe, the modern use of bioheat in buildings is large in North America and for industrial use in China and India, while biopower has a significant share of the energy consumption in all regions of the world except Africa [5].

### Bioeconomy policies

Several countries have broadened their interest in biomass as a raw material not only for energy but also for the bioeconomy. Forty-nine countries in the world have developed bioeconomy strategies and policies using a range of definitions for the bioeconomy [27, 28]. These strategies and policies seek transformation through increasing the production and utilization of high-value biobased products and materials of biological origin such as those from agriculture, forests, marine waters, or micro-organisms grown in artificial environments [29].

Bioeconomic activities are characterised by renewability, carbon benefits compared to fossil-based products, circularity with high potentials for waste reuse and recycling, and biodegradability [30]. Bioeconomic activities are also understood as having potentials for higher levels of product stability and endurance, longer lifetimes, less toxicity and less resource consumption, i.e. generally reduced levels of environmental impact. Additionally, an embedded hope is that the bioeconomy will benefit the economy, wealth generation and human health through sustainable innovation and reindustrialization [27, 30, 31]. The exact activities falling under the bioeconomy concept may vary between countries, for example, if it includes traditional uses, such as timber for construction or firewood in wood-based stoves [29].

### The conflicting views over the sustainability of bioenergy

The development of the bioenergy and the bioeconomy sectors is driven by policies aimed at more sustainable societies, mainly the mitigation of climate change. However, concerns over other undesirable sustainability impacts have grown, especially in connection with an increasing international trade of bioenergy products, sometimes leading to campaigns against the use of bioenergy, which resemble the concerns over deforestation and forest degradation that sparked the boycotts of tropical timber in the 1980s [32, 33]. The resistance against biomass feedstock production for liquid biofuels gained much of its strength in the late 2000s [34], notable after Searchinger et al. [35] published a paper warning about the increased GHG emissions caused by land use change in order to grow feedstock. In the US, though, data show that land use changes are generally caused by factors other than biofuel production [36], but concerns persist particularly related to the production of palm oil in tropical forests [37].

With the increased importation of wood pellets to EU markets in the 2010s, environmental and social concerns have gained steam. In 2011, Greenpeace Canada [38] released a report titled “Fuelling a biomess” suggesting that industrial scale “burning trees for energy will harm people, the climate and forests.” The Natural Resources Defense Council (NRDC) in the US also released a report that describes the concerns for wildlife, climate change and indigenous peoples under the heading “Our Forests Aren’t Fuel” [39]. At the time of writing in 2020, letters and opinion pieces continue to be published in the scientific literature questioning the sustainability of bioenergy feedstock production and use. For example, Norton et al. [40] conclude that bioenergy often increases atmospheric levels of carbon dioxide for substantial periods of time, but the data remains inconclusive as other researchers note errors and invalid generalisations behind this argument [41]. Other recent examples of scientific articles critical of bioenergy include Searchinger et al. [42], Booth [43], Elbein [44], and Raven and Booth [45]. Sustainability concerns have also been expressed for forest management more generally, for example, regarding the impacts of pulpwood harvesting to meet the demand for toilet paper in the US [46], and a scientific discourse on whether Europe’s forests contribute to climate change mitigation. Naudts et al. [47] assess that Europe's managed forests were a net source of carbon for most of the the last two centuries, while a large number of scientists responded to the paper saying that they do not agree and find that the applied methodology is incomplete.

While European industrial demands for wood pellets are expected to stabilize, demands of Japan and South Korea are expected to increase, with the Japanese demand likely to be met by North American producers [48] and the South Korean demand by Vietnamese producers mainly [49]. It is yet to be seen if this expected change in market development will give rise to additional sustainability concerns among international or domestic campaigners.

### Governance as a tool to reconcile conflicting views over the sustainability of bioenergy

Some of the concern over environmental impacts of economic activities has, over time, been addressed with various forms of governance. After the Earth Summit in Rio in 1992, the international tropical timber boycotts from the 1980s largely disappeared [32], sometimes ascribed to the shift in the views of Non-Governmental Organizations (NGOs) from contestation to consensus-building solutions [33]. For example, leading NGOs, including Greenpeace, Friends of the Earth (FoE) and especially the Worldwide Fund for Nature (WWF) played a decisive role in the formation of the Forest Stewardship Council (FSC) in the early 1990s in cohorts with governments and timber companies [50]. As an example related to bioenergy from the temperate and boreal zone, some of the Nordic countries also issued national forest biomass harvesting guidelines in 1980s and 1990s to meet concerns over soil fertility and biodiversity due to intensified whole-tree and residue harvesting in forests [8, 51]. Stakeholders were involved in processes to define these guidelines and some level of agreement was found on what is acceptable, which allowed intensified forest harvesting practices to continue in conditions that were assessed to have low risk of undesirable impacts. Several biomass harvesting guidelines have later been issued for various geographies and jurisdictions in North America [52] in addition to already existing voluntary or mandatory Best Management Practice (BMP) guidelines for various aspects of Sustainable Forest Management (SFM) [53].

In relation to bioliquids, WWF also played a key role in the development of the Roundtable on Sustainable Palm Oil (RSPO) certification system [54]. Sustainability criteria for bioliquids further found their way into legislation with the introduction of GHG performance criteria to the RFS in the US in 2007 [24] and a broader set of criteria, including biodiversity, to the EU RED I in Europe in 2009 [18], linking these to renewable energy policies and subsidies. The latter accepts private certification systems for showing compliance with the legislative requirements, such as an adapted version of RSPO, thus enabling enforcement also in non-EU countries [18]. The use of private regulation to show compliance with legislation has sometimes been presented as a new policy element of the EU [55], while others make the point that delegation of such responsibilities to standards’ bodies has been a common EU strategy in transnational trade for about three decades [56].

In response to disagreement about the need for EU-wide sustainability criteria for solid biomass used in heat, cooling and electricity production, the EU encouraged member states to voluntarily implement criteria corresponding to those for bioliquids [57]. As of 2020, the United Kingdom (UK) [58], Denmark [59], Netherlands [60], and Belgium have implemented such criteria [61]. The introduction did not cause major conflict among stakeholders in the UK and Denmark, while there were long-lasting negotiations with NGOs in the Netherlands until a final agreement could be reached in 2018 [62]. However, criticism persists that some of the Dutch criteria are formulated in ways that prevents implementation in practice [63], and an increasing similar pressure in Denmark resulted in the voluntary Danish Industry Agreement [59, 61] being transformed into national legislation with stricter criteria in 2020 [64].

These more comprehensive national systems will likely continue also after the adoption of a narrower set of EU-wide sustainability criteria for solid biomass under EU RED II to be enforced beginning in 2021 [19]. While the EU RED II criteria express a compromise among all member states and the Parliament, they have not resolved the conflict with campaigning societal actors who filed a constitutional challenge against the EU in March 2019 [65]. The plaintiffs alleged that EU RED II will destroy forests and increase GHG emissions and argue that this violates the environmental objectives specified in the Treaty on the Functioning of the European Union, a constitutional document. Specifically, the plaintiffs relied on Article 191(1) of the Treaty, which stipulates the preservation, protection, and improvement of the quality of the environment, with specific reference to address also climate change. In May 2020, the EU Court decided that the applicants did not have legal standing [65]. Private certification systems have also managed to gain some level of legitimacy to manage sustainability of economic activities [33, 66, 67], but researchers note various deficiencies. Fortin and Richardson [68] point out that financial compensation is too small to ensure a large uptake by producers, and a lack of credibility stemming from too much room for interpretation in the guidance documents, the occasional postponement of contentious matters, the lack of integration in the socio-politico-legal context, and the lack of effective external control systems (see also Ruysschaert and Salles [69]). Cattau et al. [70] furthermore emphasize that guidance must be more site specific, i.e. integrated with the biophysical context. Others point to problems with the use of certification systems for co-regulation under EU RED I, because the Commission’s recognition procedure and supervision of voluntary schemes is weak, leading to a lack of consistency and adequacy of the mechanisms for control and accountability [71–73]. In the context of the sustainability governance of bioenergy, Gamborg et al. [74] additionally identify the lack of transparency around value disagreement and regulatory complexity as fundamental problems that need to be resolved.

Other criticism addresses the balance of the involvement in decision-making rather than technicalities. Experiences thus suggest that reconciliation between economic actors, governments and long-established environmental NGOs around sustainability issues have led to a situation where the needs and priorities of less powerful local people are forgotten, suppressed, or neglected [33, 73]. Local people often have high stakes in the economic benefits specifically of bioenergy projects, but they, or their owner or labour associations, are typically less involved in developing certification schemes for sustainable bioenergy compared to those focusing on environmental aspects [75]. Schemes approved for the purpose of EU RED I co-regulation additionally show a solid representation of positions and interests of materially strong actors in global supply chains at the expense of weaker actors in developing countries [76]. This tendency has, for example, been seen in the RSPO [77]. The impacted actors in non-EU countries only have little access to EU decision-making processes, and the democratic legitimacy of co-regulatory approaches has been questioned [78]. The problem may seem unique to certification systems, but Pretzsch [32] also describes how many tropical state forest administrations historically failed to ensure that activities of international forest enterprises also led to long-term revenues for the local rural communities, as well as the state itself.

## Approach

We address the overall question of this paper with a feasibility analysis approach that is focused on the facilitating factors and barriers for sustainability governance to play a role in the transitioning to a profoundly more sustainable society. The situation in the bioenergy sector is used as a starting point and example to aid in better understanding the applied theoretical and abstract arguments. We see the sustainability governance crisis as pervasive to much broader scopes that also range from local to global dimensions (Sect. "Outlook").

**Fig. 1:**
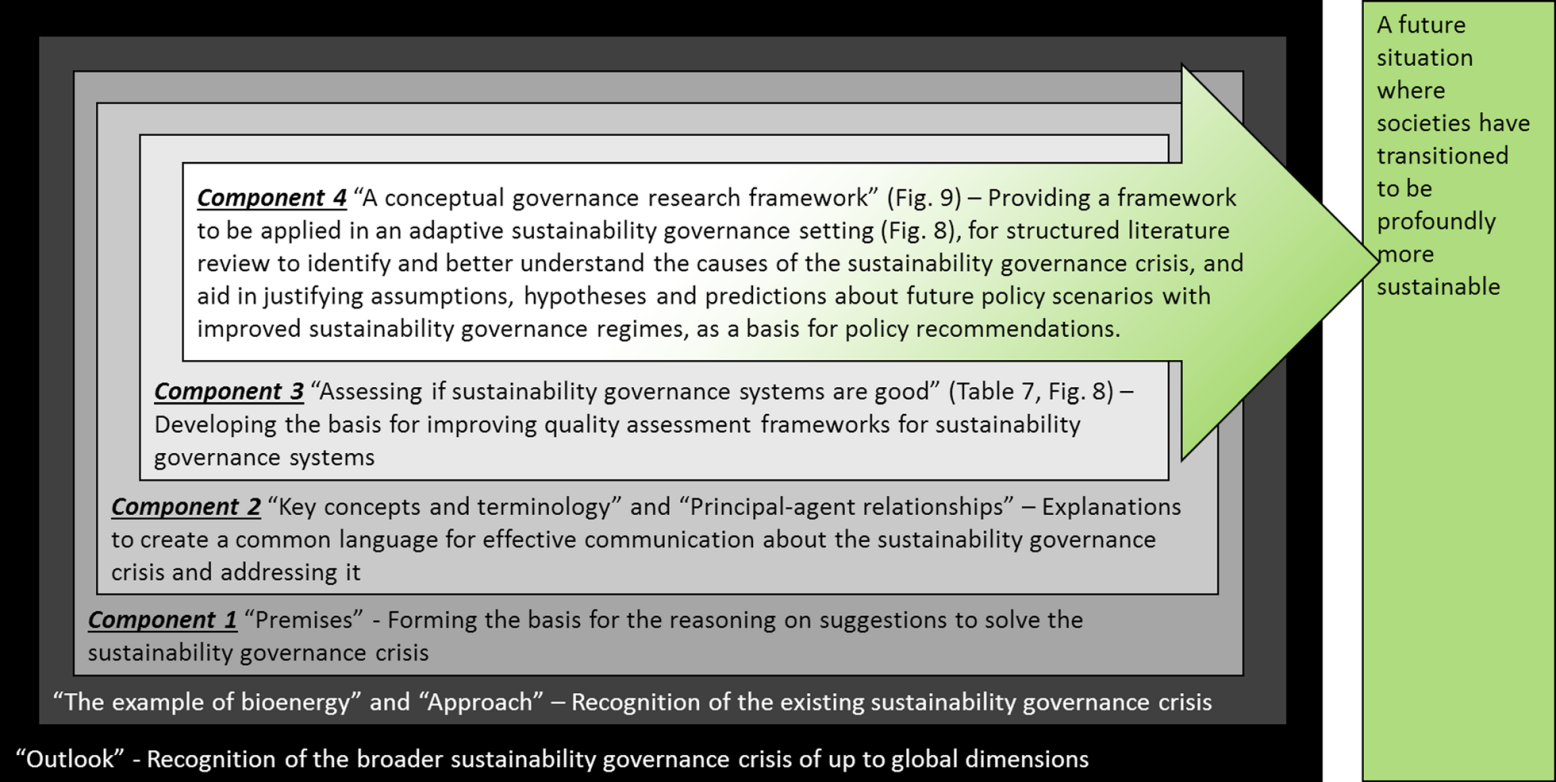
Conceptual diagram depicting the analytical approach with four components, which was applied in this paper

Our analytic approach consists of the following four components (Fig. 1):

- Component 1, Sect. “Premises” defines the premises that form the basis for the reasoning around the suggestions and arguments made in this paper.
- Component 2, Sects. “Key concepts and terminology” and “Principal–agent relationships” defines, describes and proposes basic terminology and concepts as a premise for communicating more effectively about the sustainability governance crisis as one of several barriers to sustainability transition.
- Component 3, Sect. “Assessing if sustainability governance systems are good” proposes how “good sustainability governance” can be defined based on the literature and proposes principles and criteria as a basis for developing assessment frameworks for the quality of sustainability governance systems.
- Component 4, Sect. “Conceptual governance research framework” proposes a governance research framework as a tool for adaptive sustainability governance systems to continuously review and identify the causes of existing or emerging sustainability governance crises, and design new research that generate knowledge that is useful to solve the challenges.

The work is informed by existing literature in a range of fields. Within social sciences, the paper draws upon literature from sociology, political, judicial, and economic sciences, as well as psychology, anthropology and philosophy. Within natural sciences, the paper relies upon contributions from environmental and ecological sciences, especially work conducted in an interface with social sciences, for example, McDermott et al. [79], Jones [80], Wies [81], and several other studies with conceptual content. The paper is also based on information about government initiatives and findings from several case studies, especially those addressing sustainability of bioenergy and bioeconomy supply chains, including the studies in this special issue of Energy, Sustainability and Society. The literature was used to underpin the arguments, and the paper’s intended merit is thus in the integration of knowledge otherwise largely disparate scientific fields, while comprehensive review of each topic is outside the scope of this paper.

## Underlying premises, concepts, and terminology

Deductive reasoning is reliant on logically building a bridge between a set of premises and a conclusion. Where issues are discussed under different sets of premises, whether intentionally or unintentionally, misunderstandings may arise around the made arguments and the drawn conclusions. The misunderstanding can be corrected to a disagreement if awareness around the differences in premises is increased. For example, there will be misunderstanding on the potential of sustainability governance systems to solve sustainability problems if there is no agreement as to whether a sustainability problem exists or whether there is a duty to correct it. A fundamental premise, as outlined below (see Sect. "Cooperation is needed to shape good sustainability governance systems"), is that key concepts and terminology must be clearly defined and explained. Hence, the key terms for this paper are explained and defined after outlining the premises.

### Premises

In this section we define eight premises (Table 1) that underpin the argumentation and conclusions of this paper. The premises are organized and discussed in the following three categories:

1. Human choices about our activities significantly impact life on Earth and there is a duty to care to transition towards more sustainable societies (Premises 1–2);
2. Societal trust is needed to make progress towards more sustainable societies (Premises 3–5); and
3. Cooperation is needed to shape good sustainability governance systems (Premises 6–8).

**Table 1:** Eight premises underlying the argumentation and framework proposed in this paper, separated into three categories (italics), as further explained in the main text, together with examples from the bioenergy sector

Number	Description	
*1. Human choices about our activities significantly impact life on Earth and there is a duty to care to transition towards more sustainable societies*		
Premise 1	Human choices about our activities may benefit or harm the life of other human beings and organisms on this planet	
Premise 2	Sustainability is a worthy even as an aspirational goal and all humans have a duty to care about mitigation of sustainability risks with a special obligation for those with decision-making power and influence	
*2. Societal trust is needed to transition towards more sustainable societies and governance is a tool*		
Premise 3	Trust among decision makers and other citizens or stakeholders is a critical prerequisite to transition to sustainable societies	
Premise 4	Sustainability governance is a tool to build societal trust through collaboration to reach agreement about what activities contribute towards more sustainable societies	
Premise 5	The ability of a governance system to build legitimacy and trust is affected by its design features that also influence the system’s effectiveness in achieving its goals or transition towards them	
*3. Cooperation is needed to shape good sustainability governance systems*		
Premise 6	Willingness to cooperate is needed when sustainability governance is shaped because complete agreement and trust are aspirational rather than fully realizable goals, as is sustainability itself	
Premise 7	A clear distinction between fact- and value-related disagreement is a prerequisite for constructive dialogue and reaching consensus on what activities that should be seen as sustainable, and under which conditions	
Premise 8	Efficient communication requires agreement around definitions and terminology	

#### Human choices about our activities significantly impact life on Earth and there is a duty to care to transition towards more sustainable societies

In May 2019, the Anthropocene Working Group (AWG), within the geological governing body of the International Commission on Stratigraphy, voted in favour of recognising the “Anthropocene” as formal unit within the stratigraphy. This was the first step in recognising the Anthropocene as a geological time interval. If recognised as an epoch, the Anthropocene would begin in the mid-1900s, reflecting the acceleration in population growth, industrialisation and globalisation that took place from the 1950s onwards [82]. Such recognition would signify the end of the Holocene, which is the time period following the end of the Ice Age, nearly 11,300 years ago, and recognise the modern era as one that is fundamentally dominated by human impact [83]. Domination in this context, however, is not value laden. Rather, it signifies that the modern human population is the driving force of the change in the physical and biogeochemical processes occurring on Earth [84]. There is plenty of evidence that humans make choices that benefit, or harm, various forms of life on this planet (Premise 1). The awareness of harmful impacts at global scales came to light especially in the late 1960s and 1970s, which saw the rise of the modern environmental movement in Western societies, with a key publication for its kick-off being Rachel Carson’s criticism of pesticide use in the highly influential book “Silent Sprint”, published in 1962 [85]. The latest conceptual development to recognise and address global scale impact is coined in the term “Planetary Boundaries”, which serves to explore the sustainable operating space for humanity on Earth [86].

In the case of bioenergy, scientific studies abound that bioenergy has the potential to mitigate or exacerbate environmental and social risks with a diversity of trade-offs depending on the biophysical, social, and economic context in which it is produced and consumed [87–92]. These impacts must be understood and balanced to determine the situations in which potential benefits exceed potential negative impacts.

This leads to the question of responsibility. It is a cornerstone of modern law that all persons have a “duty of care” for their neighbours. The legal concept of “duty of care” stems from early product liability cases heard in American and British courts, most significantly in 1916 and 1932 through the cases of*MacPherson v Buick Motor Co*^1^ and *Donoghue v Stevenson*,^2^ respectively [93]. More recently, duty of care is expanding in the context of environmental law [94], with the concomitant result being that governments and major polluters hold a duty of care to society to not destroy the environment, including by contributing to or exacerbating climate change. This is in addition to the long-recognised duty of care involved in traditional environmental liability cases where a private party can be held responsible for the damage caused by the environmental contamination of another’s property.

For example, in *Urgenda Foundation v The Netherlands*,^3^ the lowest court in the Netherlands, the District Court of The Hague, ruled that the Netherlands’ government had not fulfilled its duty of care to its citizens or the environment when it failed to implement adequate GHG emission reduction policies to mitigate climate change. This decision was upheld on appeal by The Hague Court of Appeal and further appeal by the Dutch Supreme Court, the highest court in the Netherlands, which stated [95]:

In short, the State has a positive obligation to protect the lives of citizens within its jurisdiction under Article 2 ECHR, while Article 8 ECHR creates the obligation to protect the right to home and private life. This obligation applies to all activities, public and non-public, which could endanger the rights protected in these articles, and certainly in the face of industrial activities which by their very nature are dangerous. If the government knows that there is a real and imminent threat, the State must take precautionary measures to prevent infringement as far as possible.

This decision marks one of the first major successes in a decade-long history of climate change litigation around the world. It suggests, along with other decisions stemming from Pakistan (mainly, *Leghari v Federation of Pakistan*^4^*)* and the Philippines (mainly, *Re Greenpeace Southeast Asia and Others*^5^), that government and major polluters must take climate change into consideration in the development of policies. Indeed, a more general duty of care regarding climate change impacts and GHG emissions may soon be recognised in jurisdictions around the world. In 2020, for example, the International Bar Association, the global governing body for lawyers, published the “Model Statute on Climate Change” [95], which is a guide for individuals and organisations to access the legal system in order to challenge government action/inaction on climate change based on fundamental legal principles.

Based on these recent legal developments, another underlying premise of this paper is that all relevant parties, including government and private actors, have a duty of care to the broader society to design policies supporting the transition to more sustainable societies (Premise 2). Because of the stewardship function of governments and multi-national or large corporations, though, these parties should especially take responsibility to make decisions that move society towards higher levels of sustainability, through sustainability policies, innovation, transformation and governance, while also being prepared to be held accountable for the impacts of their policies or activities [4, 80]. However, the obligation to help solving the challenges is on actors at all levels, including balancing the probability of achieving benefits with the risk of experiencing undesired impacts.

#### Societal trust is needed to transition towards more sustainable societies and governance is a tool

From the beginning of legal philosophy, trust has been fundamental to the social contract, whether in the context of institutional trust or trust between individuals (see also Sect. "Trust"). In the institutional context, John Locke, in his Second Treatise, argued that government is built through the onset of political society and trust, with trust being a distinct prerequisite for modern organisation of societies. Locke defines trust as a consensual agreement between the people and the ruler which is best reiterated by Geraint Parry in *John Locke*, “Government, to repeat, is not instituted by the contract. It is the recipient of a power entrusted to it for the same purpose as it was originally wielded by the society itself – the preservation of property. Governmental authority is limited by this trust and is forfeited if the trust is broken” [96]. In the context of interpersonal trust, David Hume [97] argues that trust between individuals is foundational for the functioning of a broader society: “To perform promises is requisite to beget mutual trust and confidence in the common offices of life.” Whether in the institutional or interpersonal context, it is clear that trust is necessary to keep social structures and political mechanisms together [98].

Van den Bergh et al. [4] argue that a sustainability transition cannot take place relying on market forces, but requires innovation policy and sustainability governance by legitimate powers. Based on this, we infer that a certain level of trust is necessary for sustainability transitions to take place at any scale (Premise 3). Specifically, sustainability governance can be a tool used to increase legitimacy and trust of an economic activity [74, 99, 100] (Premise 4) with the level of trust gained being affected by its design features [101] (Premise 5).

There are already several examples of well-functioning bioenergy supply chains, suggesting that the current level of trust among actors allows some level of implementation. However, several publications suggest that the potential use and benefits could grow [102] if considerable levels of conflict over sustainability and how it is best governed can be resolved. This suggests a need to intensify the discourse and improve social contracts on what constitutes sustainable bioenergy, with the way forward being improved design and increased uptake of sustainability governance systems [103, 104].

#### Cooperation is needed to shape good sustainability governance systems

Like Gamborg et al. [74], we recognise that cooperation is needed between stakeholders; governance shaped as full agreement between all actors is rarely possible. According to legal philosopher John Rawls, a just political culture is developed through the cooperation of citizens exchanging ideas. Rawls emphasizes that cooperation does not entail a compromise among worldviews, but rather is a set of doctrines that all citizens affirm [105]. Further, a political conception is only achieved where all actors are free and equal and there is a fair system of cooperation. Inspired by Rawls, we suggest that cooperation between all actors is needed to develop a sustainability governance system that has high levels of legitimacy and trust (Premise 6), not necessarily building on compromises among views, but on agreement between the actors on the premises and the set of core issues involved. We assert that prerequisites for cooperation between actors that disagree on the sustainability of a policy or an activity include a clear distinction between fact- and value-related conflicts, transparency around trade-offs between different impacts and values, as well as openness to discussing the limitations of governance as a tool to achieving sustainability goals [74], so that expectations are not unrealistic (Premise 7). The implication of not reaching any level of consensus is the lost opportunities to make further progress towards more sustainable societies. Finally, communication about facts or values can never be effective without a common language, i.e. clarity around key terminology (Premise 8).

### Key concepts and terminology

In this section we define and describe the following set of concepts and terms to ensure a common language in the discussion of sustainability governance as a tool to progress towards more sustainable societies and in interpreting bioenergy benefits and risks: “sustainability”, “sustainability transition”, “sustainability governance”, “trust”, and “legitimacy”. In the remainder of the paper, other concepts are explained in the context they are used.

#### Sustainability

The modern use of the term “sustainable” was introduced in the context of the 1972 book “Limits to Growth” [106], which argued for a “world system… that is sustainable” [107]. It was also an underlying principle at the United Nations (UN) Conference on the Human Environment in Stockholm in 1972, which was the first in a series of international conferences that considered the human impact on the environment. At this point in time, economic growth and environmental conservation were generally seen as conflicting ideas concerned with exploitation versus protection of resources [108].

By the 1980s, the early environmental movements were injected with a general concern for the social consequences of economic development, and together with environmental concerns, the issues became interweaved together in the term “sustainable development” [109]. The term was institutionalised in 1987 by the Brundtland Commission, which formulated the three pillar approach to “sustainable development” defined as “development that meets the needs of the present without compromising the ability of future generations to meet their own needs” [110]. Sustainable development was further institutionalised through the eight UN Millennium Development Goals (MDGs) [111], and the seventeen Sustainability Development Goals (SDGs) in 2015 [112].

Since its inception, sustainable development has been criticized as not being ideologically neutral. It assumes that equity, economic growth and environmental maintenance are simultaneously possible [108], with some arguing that this is an oxymoron [113]. Others have traced the concept to colonial capitalist narratives, asking “development for whom?” [109, 113]. The concept was also criticized for being more anthropocentric than eco-centric, as illustrated in the MDGs, which were focused mostly on human well-being.

For the purpose of this paper, we use the term “sustainability” rather than “sustainable development” in an attempt to abstain from ideologies about positing whether various environmental, social and economic sustainability goals can be simultaneously achievable. Sustainability, as concluded by Purvis et al. [109], has less historical baggage, and remains both context-specific and ontologically open. It thus requires a description of how it should be understood to make it operational, recognising this translation will be political in nature. This is evident from differences around the world; when defining sustainability, developed countries often focus on environmental concerns, with climate change increasingly constituting a cornerstone [75]. In developing countries, social and economic concerns are more dominant. Similar differences may also be seen between global and local scales; international NGOs are often more focussed on environmental issues, while local people often focus on jobs and economic development [75]. The sets of targets, criteria and indicators are thus context-specific for a particular time, scale, place and set of conditions, with priorities determined by the particular group of stakeholders that is relevant to the policy or economic activities being addressed. Definitions of sustainability may also consider impacts on future generations, define what an improvement constitutes [1, 114] and decide which criteria and indicators are most suitable for measuring improvements towards the targets. Improvements can be measured against a state at a certain point in time, or relative to some other trajectory of development, that will in the end involve some level of uncertainty due to human judgement about the assumptions.

For this reason, the concept increasingly gets value loaded as choices are made about which values should receive most attention, and no human endeavour is indefinitely sustainable. Additionally, improvements cannot be expected to continue infinitely over time, and sustainability is thus an aspirational rather than a fully realizable goal. We therefore suggest that “sustainability” is akin to the concept “justice”, as a high-level concept that underpins modern legal systems and society-at-large [105]. For this paper, sustainability encompasses a high-level understanding that each individual, and society, collectively, is responsible for creating a high quality environment, which includes maintaining genetic, species, and ecosystem biodiversity, air, soil, and water quality, and mitigating climate change, while also pursuing social and economic progress and that this pursuit is fundamental to society.

In the case of bioenergy and the bioeconomy, the three environmental, social, and economic pillars form the basis for defining what activities are sustainable and what activities are not. Bioenergy activities may impact the biophysical factors and ecological environment, including soil properties, primary productivity, surface and groundwater quantity and chemical composition, biodiversity, and GHG emissions, energy use, or waste disposal [115–118]. The possible social impacts include equity in access to resources and energy, respect for workers’ rights, equitable wages, safe working conditions, general human health, welfare of communities [119], and livelihoods and rights of indigenous communities [120]. Sustainability criteria for bioenergy and the bioeconomy may also seek to promote economic opportunities through creation of favourable framework conditions or direct support for development of industries, new supply chains and innovation in energy systems [4, 121, 122]. However, it remains an assumption that trade-offs must often be made among criteria [118, 123, 124].

#### Sustainability transition

Achieving higher levels of sustainability can be seen as a question of transition. Based on van den Bergh et al. [4], we define “sustainability transition” as a transition towards a more sustainable society based on goals and criteria of what a sustainable society means agreed upon by involved actors. Societal transitions will generally take place through innovation, technology transfer or governance, even if catastrophic events may also be a powerful driver [4].

The literature is clear that a sustainability transition is laden with difficulties. Van den Bergh et al. [4] point out that innovation takes time, requires costly research and development (R&D) investments and involves many failed efforts in order to reach a stage with market up-scaling. Van den Bergh also discusses an “energy and environmental rebound”, which reduces the expected gains from new technologies because of changes in behaviour incorporating the common second-order off-set effects. The authors argue that, in the short-term, changes are more likely to happen through adoption of existing technologies, and refer to economic studies showing that the major part of reduction of GHG emissions in the coming decades is more likely to come from environmental regulation rather than technological innovation [4].

Imperfect design of an environmental policy may, however, lead to unintended negative environmental consequences, as illustrated by the “green paradox” coined by the German economist Hans-Werner Sinn. It refers to the “leakage effect” or “announcement” effect of imperfect policies [125]. The leakage effect occurs when operations move to an unregulated jurisdiction, thereby avoiding the effects of the regulation. The announcement effect occurs when there is a time gap between the announcement of the policy and the implementation of it, incentivising firms to increase emissions for increased profits in the intermediate period [125, 126]. A similar phenomena might occur in the Land Use, Land Use Change and Forestry (LULUCF) sector, where new EU-wide GHG emission accounting rules for the LULUCF sector from 2021 [127, 128] by some parties are seen as limiting for future harvests. There are indications that forest harvesting has increased in some countries since 2015, even if a causal link with policies have not been established [129]. The ultimate consequence of the “green paradox” is that no jurisdictional policy can impact the least sustainable actors on the globe, leaving a binding global agreement as the strongest available policy, for example, in the fight against global warming [130].

Environmental policies have also been suggested to potentially lead to unintended positive environmental consequences, for example, the “Porter hypothesis” posited by Porter and van der Linde [131]. It predicts that environmental policies may lead to improved productivity, economic performance and competitiveness through environmental innovation, also known as eco-innovation, when companies seek towards more efficient material and energy use in response to environmental policy incentives or laws [132]. While both the green paradox and the Porter hypothesis are logical explanations to observed phenomena, their validity are still being debated.

Another point has been made, that if only environmental regulations are in place, currently cost-effective technologies would be favoured over less mature technologies that might be more desirable innovative alternatives in the long term [4]. Benefits that require a longer time horizon to realize may be promoted by policies that provide financial support for eco-innovation and renewable energy subsidies. Some have advocated that innovation policies can substitute environmental policies, but reliance only on eco-innovation policies might have undesirable consequences as it may increase a firm’s performance and opposite to Porter’s hypothesis lead to increases in raw material and non-renewable energy consumption, and cheaper prices that may also lead to increasing end-user consumption. Therefore, accompanying policies and regulations are needed so that consumption is limited. For example, renewable energy subsidies should be accompanied by restrictions to avoid second-order effects, such as cheaper energy and enhanced total supply of energy (e.g., electricity).

Kemp and van Lente [133] argue that sustainability transitions not only include the challenge of changing technological systems, such as an energy system, but also the challenge of changing the criteria by which consumers judge the appropriateness of new products, services and systems. The authors claim that without fundamental change in consumer behaviour, a transition towards a more sustainable society is unlikely to take place. Compared to pure technological transitions, sustainability transitions require not only technology change, but also a cultural change in technology use. An example is that car technologies may change from combustion engines to electric, but the change will also need to be accompanied by a shift in the public perception of electric vehicles as attractive means of transportation, and a shift in life style towards less total demand for transportation. Another example is the circular economy, which will not be realised unless views of waste change to viewing it as a resource. Both van den Bergh et al. [4] and Kemp and van Lente [133] conclude that as long as consumers’ desire for comfort, convenience and low costs dominate as the criteria for judging the appropriateness of products, services and systems, it will be difficult to transition towards more sustainable societies.

Bioenergy plays a prominent role in many nations’ goals for renewable energy and climate change mitigation, but as suggested by Kemp and van Lente [133], its uptake requires its acceptance as a renewable and sustainable energy source by consumers. Some authors question the extent to which bioenergy will able to advance sustainability agendas through innovation or governance [73]. In spite of ideological resistance and concern about the capacity of existing forests to meet the increased demand for wood fuels in a sustainable way, Gazull and Guatier [134] argue that a broadening of positions is bound to take place. They predict that the demand for energy and resources will be a driver for bioenergy development and acceptance, as will pressures from new industrial players. However, they also recognise that there may be undesired sustainability impacts associated with this transition.

#### Sustainability governance

We see governance as a collaborative tool that can contribute to finding solutions to sustainability challenges and building adequate levels of legitimacy and trust for their deployment and implementation (Premise 4). We start with the concept of "governance”, which can be described as the process of decision-making, including decisions made about the implementation of activities and solutions at international, regional, national or local scales [135].

The executing bodies in governance may be state or non-state actors [136]. State actors include domestic and international government agencies; and non-state actors include private corporations and businesses, communities, private independent third-party initiative NGOs, citizen movements [2, 66], indigenous peoples and local communities [137] or informal groups [138]. Public regulatory regimes include governmental regulation, ordinances, guidelines, BMPs, educational programs, and public awareness campaigns. International agreements and conventions with nations as signatories also fall under public governmental regulation. Regulatory regimes by non-state actors include private certification systems, i.e. standardization, company policies, e.g. Corporate Social Responsibility (CSR) [139], organisations’ or communities’ BMPs and education programs [52, 140].

**Fig. 2:**
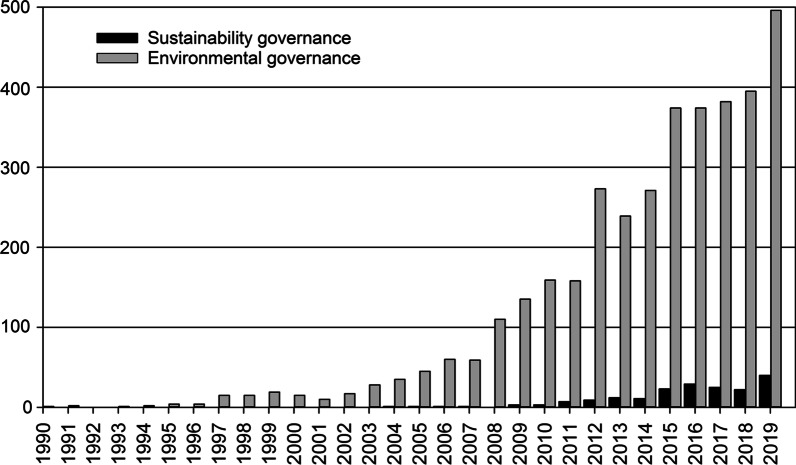
The number of new publications each year during 1990–2019 with the occurrence of the terms “sustainability governance” and “environmental governance”, respectively, in the title or the abstract in the Web of Science publication database, 1 June 2020

The complex and multi-scalar character of many critical environmental problems has changed the regulatory focus from “government” to “governance” as traditional governmental approaches in many cases have shown to be ineffective for solving the problems they intend to solve [136]. The term “environmental governance” has emerged in the scientific literature starting in the late 1990s (Fig. 2), defining a situation where several interdependent government and non-government actors work together to achieve environmental goals [2]. The term “sustainability governance” emerged later in the literature, starting from the mid-2000s [141] (Fig. 2). This term is inclusive also of the social sustainability dimension and is sometimes described as “steering for sustainable development” [1, 142].

**Fig. 3:**
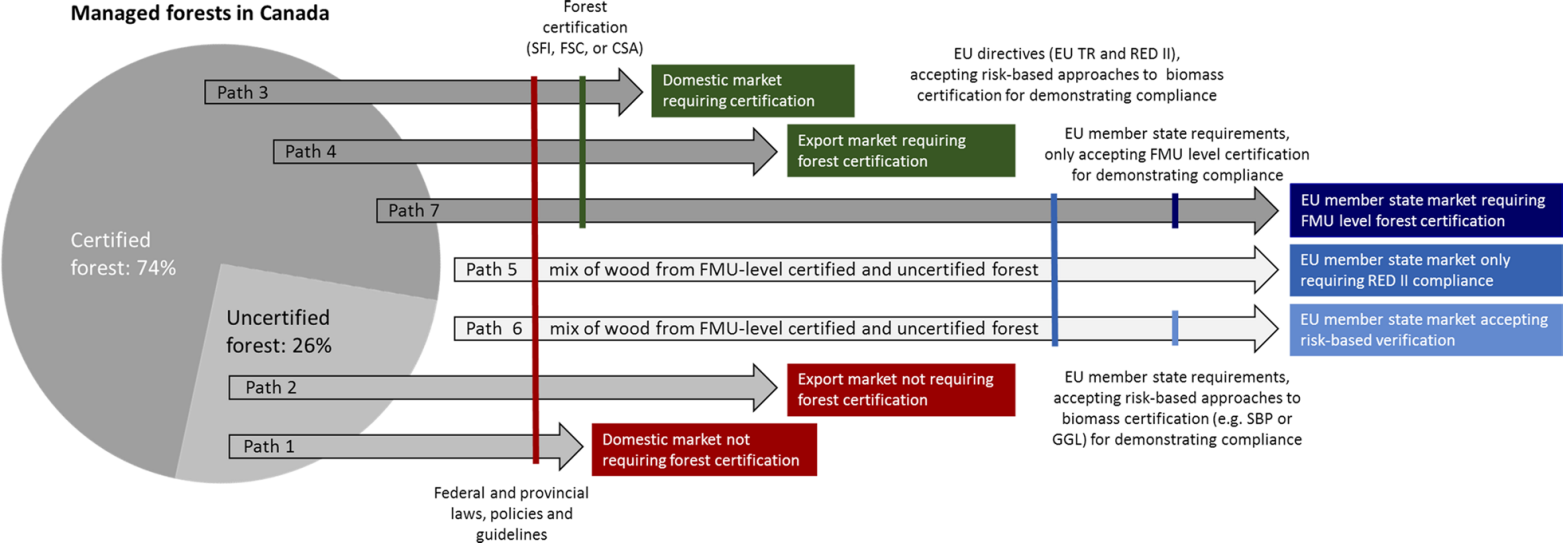
Pathways indicating the combination of Sustainable Forest Management (SFM) criteria or standards that a regulated forest biomass producer in Canada must meet to sell in a specific market. SFI: Sustainable Forestry Initiative, FSC: Forest Stewardship Council, CSA: Canadian Standards Association, EU TR: European Union Timber Regulation [149], RED II: EU Renewable Energy Directive of 2018 [19], SBP: Sustainable Biomass Program, GGL: Green Gold Label. Redrawn and adapted from Kittler et al. [143] with information input from Rachel Dierolf (pers. comm. 2020)

The number of applicable governance systems and how they are linked and interact can be highly complex in particular situations. For example, wood pellets exported from the US [143] or Canada to Europe for use in the heat and electricity sector involve multiple layers of governance, including international, federal, and state or province levels (Fig. 3) [143]. Multilevel or multilayered governance has more commonly referred to policies adopted in a higher level, e.g. federal, which must be implemented at a lower level, e.g. state or province, or country in the case of the EU [144], with “city” potentially being a third level [145]. However, with intensified globalisation, the state’s responsibilities and capabilities have been changing, leading to a broader view on how activities can be governed. Formal and informal participation and influence of non-state, private actors are increasingly accepted as a supplement to public regulation [146].

For the purpose of this paper, we understand sustainability governance as broadly as possible, including systems that may involve multiple levels of relevant governance schemes, formally related or not. We thus define sustainability governance as a set of formal and informal processes and mechanisms, undertaken by public or private actors that are formalised organisations, and which, alone or in collaboration with other organisations, seek to influence the actions and outcome by either state or non-state actors in the direction of a more sustainable society, based on defined sustainability goals. Such broader understandings of sustainability governance are shared with other authors, including Hogl et al. [2], Gamborg et al. [74], and Gunningham [147].

Bioenergy is a prominent example of a sector where multiple levels of sustainability governance are common (Fig. 3). The sustainability of bioenergy is already governed through several state and non-state tools that are more or less coordinated. Furthermore, the diversity of sustainability governance systems relevant to bioenergy applies from international to local levels and relies on regulatory regimes within many sectors such as forestry, agriculture, nature conservation, spatial planning, waste, energy and transportation [148].

#### Trust

In order to face the sustainability challenges of today, adequate levels of mutual trust between involved actors is needed. At the individual level, the concept of trust is intertwined with the virtues of honesty and integrity and has been described as “the feeling that the other would never do an injustice to one” [150]. Burlea and Tomé [150] expand the understanding of trust to public spaces and public organisations such as those between states and society, and corporations and their stakeholders.

Such higher levels of trust are expressed through the concepts of “social trust” and “institutional trust”. Institutional trust concerns the trust between societal members and a public institution. Jackson and Gua [151] characterise institutional trust as the belief “that the institution and enforcement officers use their power in judicious, restrained and appropriate ways”. As others [152–154], they base trust in “normative congruence” to justify power, where normative congruence is understood as the shared values between the community members and the governing institution. High levels of institutional trust results in voluntary cooperation, meaning that people will subscribe to the influence of the governance institution without much resistance and the governance institution will perform to the expectations of the people.

While social trust is inclusive of institutional trust, it may also include the trust among societal individuals and organisations. When there is societal trust, the community and its individuals share values and identities amongst its members [155]. Social trust has been long understood as a foundation for social order [156], which produces social regularities that may be more stable than structure resulting from self-interested or habitual pursuits [151]. As long-term investment prefers predictable conditions, it is crucial that social and institutional trust are established and exist at some level, if a long-term sustainability transition is to take place.

At the corporate level, a corresponding concept is the “Social License to Operate” (SLO). SLO expresses the mutual trust between economic operators and their stakeholders. SLO may be defined as the “community’s perceptions of the acceptability of a company and its local operations” [99]. Trust is also integral to this concept based on normative justifiability of the operator and its activities, with perceptions of an enduring regard for each other’s interests. Stakeholders perceive that the company is listening and responding to their needs and that it keeps its promises, engages in mutual dialogues, and exhibits reciprocity in its interactions with the stakeholders [99, 157].

In a study of trust as a “multilevel phenomenon across contexts”, Herian and Neal [158] similarly separate between three levels: individuals, groups and institutions, and advance the understanding of trust by creating a conceptual model with overlaps among different levels. Since governance of an activity or a value often implies both formal and informal collaboration among a variety of different actors, it is relevant to consider the nature of the mutual trust at different levels and among these levels. Based on Burlea and Tomé [150], we distinguish between three different levels: societal non-economic actors, economic actors in the market, and governmental institutions (Table 2). We include individuals and NGOs under “societal actors” and private actors operating along different supply chains as producers, traders, buyers, consultants and in certification under “economic actors”. This paper focuses on the trust between societal actors and economic operators and societal actors and governmental institutions.

**Table 2:** Types of social contracts through which trust is granted and received at three levels, inspired by Burlea and Tomé [150]. See Sect. "Principal-agent relationship" for an explanation of agency and principal–agent relationships

Receiving → (agent)		Individuals, societal actors		Economic operators in the market		Governmental institutions	
Granting ↓ (principal)		Type of trust granted to individuals by…	Strategies of individuals to receive trust of…	Type of trust granted to operators by…	Strategies of operators to receive trust of…	Type of trust granted to governments by…	Strategies of governments to receive trust of…
Individuals, societal actors		Inter-personal trust to depend on the other	Agreements on how to work together	Social License to Operate (SLO)	Corporate Social Responsibility (CSR) policies	Institutional trust, obey e.g. to tax payments	Effectiveness of government, democracy
Economic operators in the market		Provisioning of goods and services	Resignation from naming and shaming campaigns/ employees acting on behalf of their company	Trust to collaborate within the supply chain	Agreements on how to work together	Economic prosperity, tax payments	Create a stable business environment, effectiveness of government
Governmental institutions		Freedom, equality, welfare	Law-abiding behaviour	Governmental license to operate	Law-abiding behaviour	International relations and collaboration	Agreements on how to work together

Studies of trust may include considerations of its opposite, i.e. “suspicion”, with this being seen as another dimension rather than a contrast. Trust and suspicion may occur in the same relationship [155] resulting in a need for means to alleviate suspicion through, for example, monitoring and documentation. Monitoring and documentation may thus be tools to continuously keep up the level of trust, which is no longer entirely based on normative congruence (Table 3). A complication in the absence of normative congruence can be a power struggle between the various groups of actors involved in the granting of institutional trust or SLO [155]. However, the opposite situation, where trust relies entirely on normative congruence, may not be ideal. With no control or transparency but only trust, people will be left susceptible to manipulation and exploitation [159]. Overreliance on trust in interpersonal relationships can also lead to poor business decisions and nepotism.

**Table 3:** Integrated understanding of trust and suspicion, simplified and adjusted for the purpose of this paper after Lewicki et al. [155]

	Low levels of suspicion	High levels of suspicion
High levels of trust	Trust by normative congruence Poor incentive for monitoring and control Prone to emotional and ideological manipulation	Trust by verification High incentive for continuous monitoring and control Possibly prone to data manipulation, unless data quality is also monitored and controlled
Low levels of trust	Limited interdependence Poor incentive for monitoring and control	Harmful motives assumed and potential paranoia and conspiracy theories Monitoring and control is disbelieved Believe in ideology or “pathos” and “ethos” and disbelieve in data or “logos” is common Prone to emotional and ideological manipulation

Several of the constructs presented here are relevant to the study of trust in governance of sustainability of bioenergy. As mentioned, regulating activities take place at several levels and across sectors, which makes it crucial to consider both peer trust and trust among different levels. Normative congruence in the field would imply that sustainability is generally seen as a worthy goal (Premise 2) and actors have the same understanding of what constitutes the sustainability of bioenergy and what metrics are needed to determine whether something is sustainable or not. In bioenergy such normative congruence exists to some extent (Premise 4) but not to the extent considered ideal by some actors. For example, the energy industry accepts the sustainability criteria of EU RED II as useful, but it is not adequate to gain trust with some NGOs and individuals, cf. also Sect. "The conflicting views over the sustainability". The approach taken by the EU through RED I, RED II and by EU member states via their national schemes assumes there will be higher levels of trust if verification of agreed criteria is provided. Such strategies may be effective in the interaction with people that have high levels of both trust and suspicion, while it is unlikely to work as a strategy to connect and make progress with the groups that have a low level of trust and a high level of suspicion. A study by Baumber [160] analyses recent studies on cellulosic energy cropping to determine the extent to which they consider the issues that are key obtaining SLO. While issues such as distributional or procedural fairness are occasionally addressed, trust has only received little coverage. This highlights a risk that trust may be an overlooked factor to consider in the future by researchers, bioenergy proponents or policy makers [160].

#### Legitimacy

**Fig. 4:**
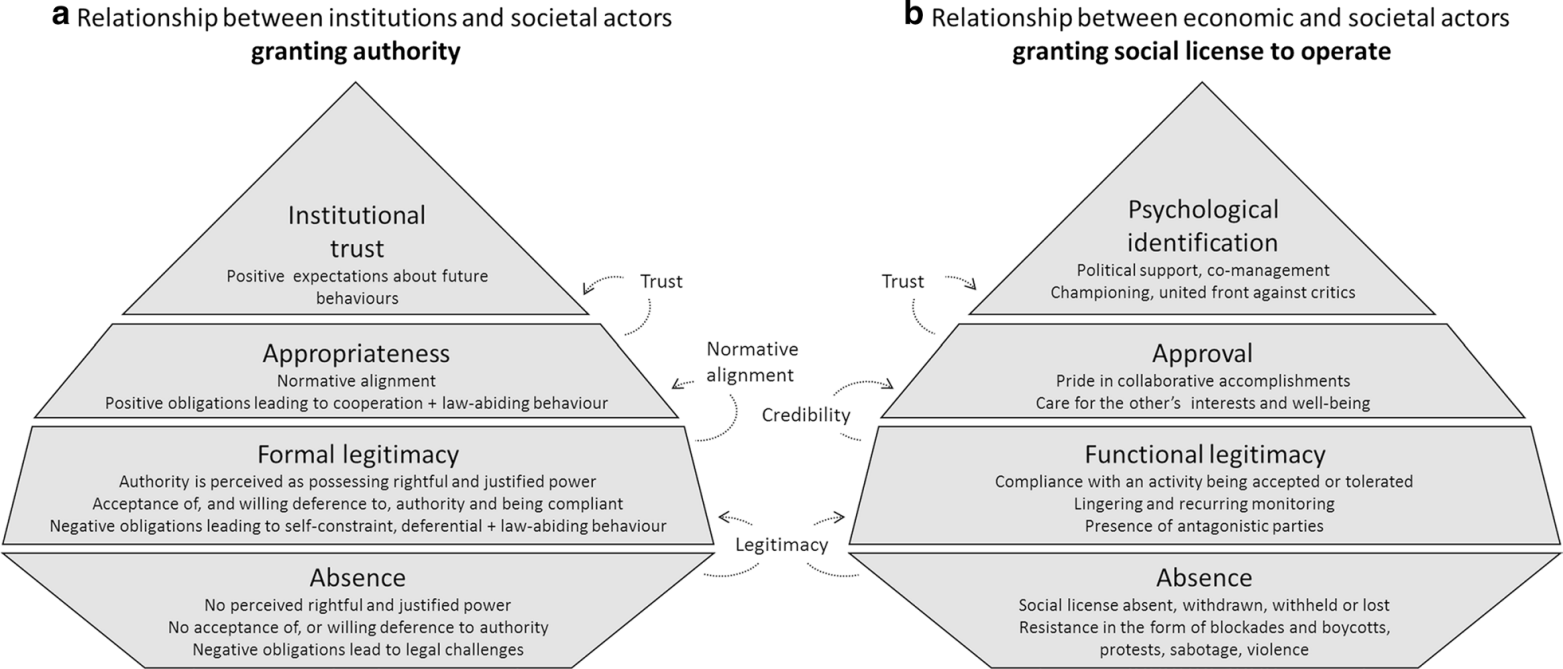
Conceptual model of how trust relates to legitimacy, inspired by Jackson and Gau [151] (a) and redrawn and adapted from Thomson and Boutilier [100] (b), respectively

Trust and legitimacy are related concepts but still differ in nature. The relationship between them can be conceived in models as a conceptual overlap [151] or with legitimacy as a precondition for granting of trust [99, 100, 157] (Fig. 4). The models of Jackson and Gau [151], and Thomson and Boutilier [100] were developed in the context of institutional trust and the SLO, respectively. Even if the rationale for each of them is similar, a fundamental difference is that a law is a formalised legal contract, which defines clauses and actions for involved parties, while an SLO is a process of continual negotiation [161].

Jackson and Gua [151] define legitimacy as the “property or quality of possessing rightful power and the subsequent acceptance of, and willing deference to, authority.” Legitimacy is not inclusive of personal relationships between individuals but includes relationships of individuals with formalised organisations and institutions. It does not necessarily coincide with trust as individuals in society may be willing to obey a law or its enforcement officers if they see the law as legitimate without agreeing with substance or content [151] (Fig. 4a).

Thomson and Boutilier [99] and Boutilier and Thomson [162] see legitimacy as the threshold where stakeholders move from the first of four distinct levels of the social licence, i.e. the level where social licence is absent, withdrawn, withheld or lost, to the second level where an activity is accepted or just exactly tolerated (Fig. 4b). The first level is marked by behaviours such as shutdowns, blockades, protests, violence, sabotage, and legal challenges, while the second stage is marked by lingering and recurring monitoring, and the presence of antagonistic outside parties. Credibility allows the activity to proceed to the next level of social license, where the activity may be seen as having achieved approval with the company seen as good neighbour and there is pride in collaborative accomplishments. Finally, trust allows movement to a level of social license with “psychological identification” where there is political support, co-management of projects and a united front against critics from the outside. The three categories of Jackson and Gua’s model [151] (legitimacy, appropriateness and trust) can perhaps be seen as corresponding to three levels of Thomson and Boutilier’s model [99] (acceptance, approval and psychological identification). At the highest level there is normative congruence between stakeholders and the company or the institution.

### Principal–agent relationships

**Fig. 5:**
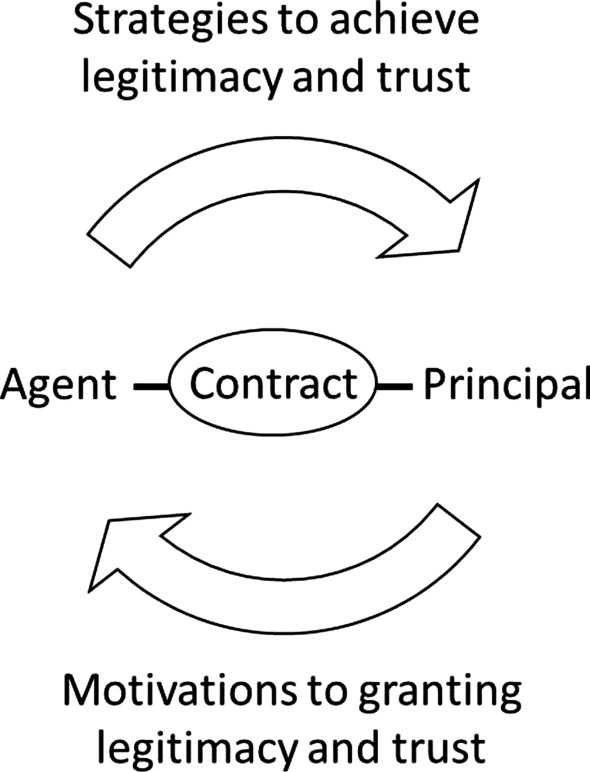
Illustration of the principal–agent relationship based on a formal or informal contract agreed upon by the parties

A key concept in the study of legitimacy is the principal–agent relationship (Fig. 5). The principal–agent relationship is an arrangement in which one entity, the “principal”, appoints another, the “agent”, to act on its behalf [163]. The relationship between the principal and the agent is called the “agency”. Agency relationships exist in formal and informal settings—in formal settings, for example, government action on behalf of citizens, or employee action on behalf of a corporation. In informal settings, children may complete tasks on behalf of a parent without a contractual relationship (Table 3). Agent and principal analysis frameworks may serve as a basis for further understanding who is influencing governmental or business policies, who has a stake in the policies, and why principals or agents might grant or achieve legitimacy, respectively, based on the appropriateness of the informal or formal contractual agreement.

#### Types of agents and agent systems

**Fig. 6:**
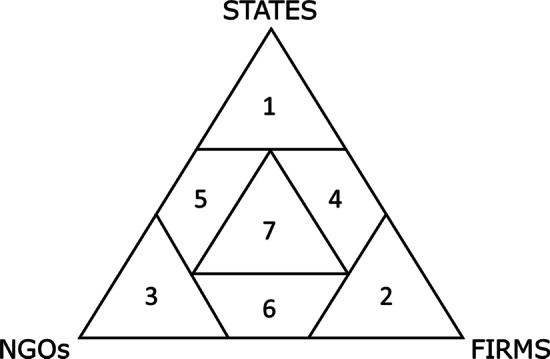
“The Governance Triangle” showing seven categories of governance systems based on the main types of involved actors. The associated types of regulatory activities can be described as (1) traditional top-down legal standards, typically laws, (2) self-regulation, (3) third-party private regulation, (4) standards of firms influenced by states (co-regulation), (5) standards of NGOs influenced by states (co-regulation), (6) joint efforts between firms and NGOs, (7) joint efforts between firms, NGOs, and states (transnational regulation). Redrawn after Abbott and Snidal [165] and Purnhagen [166], see also [170]

In the early days of environmental governance, the agent was typically synonymous with government. Today, governmental control over sustainability governance is increasingly being shared with other actors [147, 164]. Based on Abbott and Snidal [165], Purnhagen [166] proposes a typology for the governance system agent, where the agent is classified as various combinations of three major types of agents, including the state, firms and civil society actors such as NGOs. Seven categories are defined by the degree to which each of three main types of agents are involved (Fig. 6) ranging from the state as the single agent, with traditional law as the social contract, to newer systems that involve two or all three types of agents. These systems are also known as hybrid systems. The last three decades have seen a growing number of various hybrid systems [165], where the different types of actors co-regulate.

Hybrid systems emerged in a national context [147, 164] but have proven especially useful for the purpose of international trade and associated transnational governance [167] that are embedded in and supported by other modes of governance [168]. Transnational governance may be described as modes of governance, which “structures, guides and controls human and social activities and interactions beyond, across and within national territories” [168]. The emergence of such structures is seen as a sign that no single actor can address the multiple facets and interdependencies of environmental or social problems arising as a consequence of international trade with transboundary sustainability impacts [136, 167]. Cashore [66] suggests that an advantage accrues where an actor brings their own strength, for example, the assumed efficiency of a private firm, the knowledge base and social trust of an NGO, or the democratic legitimacy of a state actor. Similarly, Ewert and Maggetti [167] conclude that hybrid agents allow organisations to gather the competencies required in each case for the most efficient and effective approach to govern sustainability along the entire supply chain.

The nature of the hybrid agent may differ. Cafaggi [169] classifies the nature of their cooperation as “co-regulation”, “delegated co-regulation”, and “ex-post recognised private regulation”. In co-regulation, private regulators take part in different stages of the public regulatory process, for example, certification schemes approved to show compliance with EU RED I [170]. In delegated co-regulation, a public authority recognises private regulators for its own regulatory purposes, for example, the Danish Industry Agreement for sustainable wood chips and wood pellets, where the government approved a scheme developed and operated jointly through Danish energy producer associations [62]. Finally, in ex-post recognised regulation, private actors regulate autonomously and independently of the state with the initiative subsequently being recognised by public authorities for their purpose. For example, the state’s recognition of private forest certification systems for state procurement policies constitutes an ex-post recognised private regulation [170]. Hybrid governance systems may also take the form of “network governance” or “meta-governance”. Network governance is carried out by networks of various public and private actors with capacity to solve a specific problem and enhance participation in policy making [171]. Such networks are often seen as the most effective in a policy setting characterised by “a multiplicity of social and political actors, vague and incomplete problem definitions, the need for specialized knowledge, conflicting policy objectives, and a high risk of political antagonism.” Meta-governance can be defined as the governance of governance to make sure that formal or informal governance processes operate according to the prevailing conceptions of what constitutes “good governance” [171], see also Sect. "Assessing if sustainability governance systems are good".

#### The principal

In the late 1900s, business communities started to show interest in stakeholders and how to engage with them as firms’ reputations, success and survival increasingly became vulnerable to the influence of societal groups and movements [172]. Environmental issues, too, increased their potential to mobilize societal actors with a prominent case from the mid-1990s being Shell seeking to dump the oil rig, Brent Spar, into the sea but giving up due in part to boycotts and naming-and-shaming campaigns [147]. This triggered the need to understand more about the people and groups with the potential to obstruct or support institutional goals, whether to defeat, neutralise, mobilise or empower them. These people and groups are captured under the term “stakeholders”, which is understood as persons and organisations that have a stake, interest or concern in relation to a policy or decision being made, such as those by businesses or governments.

The scientific literature addressing stakeholders has increased since 1990 when only 45 publications could be found in the Web of Science database, but increased to 2,890, 24,631 and 123,828 in the years 2000, 2010 and 2020, respectively. A commonly used analysis framework classifies stakeholders into four categories based on their interest and influence relevant to a certain business activity (Table 4).

**Table 4:** Categorisation of stakeholders along the two dimensions of interest and influence based on text by Reed et al. [173]

	Low level of interest	High levels of interest
High level influence	“Context setters” May be a significant risk Should be monitored and managed in case of risk	“Key players” Are often influential supporters Should be actively groomed
Low level of influence	“Crowd” Have low interest and influence There is only little need to consider this group, except monitor if their status changes over time	“Subjects” Are often supportive but lack the capacity for impact, although they may over time become influential by forming alliances with other stakeholders Should be empowered if they are supportive, and managed if they are unsupportive, especially if they gain influence, e.g. through alliance

The analysis can be improved by adding other attributes, for example, if stakeholders are more or less supportive of the activity. Such a descriptive analysis is not a goal in itself but may form a basis for elaborating strategies on how to engage with different groups of stakeholders. The model has been further elaborated by Reed et al. [173], who propose that involving stakeholders can help create more meaningful and relevant stakeholder analyses. Stakeholders may, for example, bring insights by identifying other relevant stakeholders, categorizing themselves and other stakeholders, and describing the relations between each of them. The outcomes and categories of such a process may deviate from the framework outlined in Table 4, as was the case for a typology developed for stakeholders to wind turbine conflicts within forests in Germany and the US, where seven different actor types of participants were identified, depending on their role and attitudes in decision-making in a multilevel governance setting [174].

As digital innovations are gaining interest as tools for stakeholder engagement, new stakeholder analysis frameworks are emerging. Lutz and Hoffmann [175] propose a framework that differentiates between participation and non-participation, active and passive, and positive and negative (non-)participation, resulting in eight stakeholder categories (Table 5). However, it remains to be examined if such frameworks can provide additional understanding in a sustainability governance and bioenergy context.

**Table 5:** Typology of online participation along three dimensions: participation (participation / non-participation, activity (active / passive), and attitude (positive / negative)

	Participation		Non-participation	
	Active	Passive	Active	Passive
Positive	Constructive engagement engaging audiences online for a purpose commonly accepted as beneficial	Incidental contribution moved to engage by others without any genuine intention but in a beneficial way	Abstention refrain from an activity to further a cause considered socially desirable	Lack of awareness or motivation no awareness of any need to engage in an activity
Negative	Destructive engagement engaging for a purpose widely considered harmful or undesirable	Involuntary imposition drawn into forms of participation deemed detrimental	Silencing, self-censoring refrain from online engagement due to perceived pressure or threats	Exclusion exclusion from participating despite the potential usefulness of participation

From Lutz and Hoffmann [175]

#### Principals’ motives to grant legitimacy and trust

The principals’ motives to grant legitimacy to the agent can vary. The literature on granting and achieving trust or legitimacy often exhibits a similar, even if not identical, conceptual understanding of these motives and processes, regardless of the focus on individual, organisational or institutional levels (Table 6). Based on examples from the literature, we classify motives into the following five categories: egoism/hedonism, altruism, tradition, value and observation. Motivations to grant legitimacy and trust may thus be self-interested, may be entirely concerned with other people’s interest and well-being or may be concerned with various degrees of reciprocity. Legitimacy and trust may also be granted based on what is already well-known and familiar, or for moral and normative reasons. Finally, motivations may include perceptions of what is meaningful and appropriate.

**Table 6:** Comparison of different scholars’ classification of motivation to grant trust or legitimacy or the process in which this takes place, based on the principal’s perceptions about the agent and its conduct

Category of motives	Description of motivation or process leading to granting of legitimacy or trust, based on the principal’s perception of the following:	Scholars (principle–agent)				
		Aristotle^a^	Max Weber^b^	Mark C. Suchman^c^	Thomson and Boutilier^d^	Burlea and Tomé^e^
		(Individual-individual)	(Individual-public authority)	(Society-institution)	(Stakeholder-corporation)	(Individuals- organisations)
Observation	Coherent, understandable, and meaningful activities or automatic conformance with developments in societal priorities	–	–	Cognitive legitimacy	–	–
	Competence, rule of law	–	Rational authority	–	–	Competent trust
Value	Positive normative judgment, shared values, and perceived benefits for society	–		Moral legitimacy	Socio–political legitimacy	–
	Perceived high level of personal virtuousness and integrity	–	Charismatic authority	–		Referential trust
Tradition	Tradition, and what has always been there (feudalism, religion)	–	Traditional authority	–	–	–
	Family or group identity	–		–		Identitary trust
Altruism	Enduring mutual regard for each other’s interests	Friendship due to goodness	–	–	Institutionalised trust (identification)	Affective trust
	Reciprocity in interactions, where the agent listens and responds to the needs of the principal, keeps promises and engages in mutual dialogue	–	–	–	Interactional trust	Optimistic or mutual trust
Egoism, hedonism	Achieving self-interested benefits	Useful friendship	–	Pragmatic legitimacy	Economic legitimacy	Opportunistic trust
	Achieving pleasure	Pleasant friendship	–	–	–	–

^a^Kraut [176]

^b^Smith [177]

^c^Suchman [178], Cashore and Stone [179], Burlea and Popa [180], Nielsen [181]

^d^Thomson and Boutilier [100], Boutlilier and Thomson [99], Gehman et al. [157]

^e^Burlea and Tomé [150]

Based on results from neuroscience, Herian and Neal [158] argue that the processes that underlie people’s judgments about companies differ from those that underlie their judgement about other people, but it is still an unresolved matter if it is useful to theorise about intergroup or inter-institutional legitimacy and trust based on measurements at interpersonal levels or if different conceptual understandings are needed at these “higher” levels.

#### Agents’ strategies to achieve legitimacy and trust

The agent may elaborate and apply different strategies in the pursuit of principals’ granting of legitimacy and trust, depending on assumptions about their motives to grant legitimacy, or based on information from stakeholder analyses.

Almunawar and Low [182] suggest that Aristotle’s modes of persuasion can be translated into the context of social trust in organisations, with a shift in focus from an individual’s personal trust in another individual to stakeholders’ impersonal trust in an organisation. Aristotle’s modes of persuasion include the following four elements: pathos, ethos, logos and kairos. Pathos is the appeal to the audience’s emotions, while ethos the speaker’s conveyance of authority, credibility or charisma. Logos is the appeal to the audience’s sense of logic through data, facts and figures and kairos refers to the selection of a time and space where the audience will be open to the message. Almunawar and Low [182] translate the first three concepts into “appearance”, “reputation” and “performance”, respectively. Appearance deals with the agent’s emotional influence on the principal, while reputation has to do with the agent’s credibility, for example, through perceived ethics, integrity, documented competences and demonstrated good intentions. Reputation has increasingly become volatile in the past decades, as sensitive statements can quickly go viral. In times of extreme crises, such as the COVID-19 pandemic, it may be decisive for future reputation and company survival if companies show their good intensions to help society [183]. Performance is the presentation of sound evidence about impacts, for example, through statistics or scientific data. All three modes should be jointly used to optimize the chance of organisations or institutions to convince the principals to grant legitimacy and trust.

Based on observations of institutions, Suchman [178] develops a conceptual model to further understand how institutions form strategies to achieve legitimacy. His model has been applied in several other governance-related studies [66, 180, 181]. In Cashore’s [66] version, the model distinguishes between “conforming”, “manipulating” and “informing” strategies. Each of these strategies can be developed in different directions depending on knowledge about the principals or stakeholders and what motivates them to grant legitimacy. A conforming strategy may thus aim at conforming to the principals’ selfish needs, moral ideas, or external sources depending on the principals’ “pragmatic”, “moral” or “cognitive” motive to grant legitimacy. A manipulative strategy correspondingly aims to manipulate the principals’ self-interested needs by advertisement, undertake activities that have spill-over effects to moral ideas, or promote the legitimacy of activities as if they are already taken for granted. Finally, informative strategies aim to “[get] the word out there” to more people with self-interest, explain how activities match up with societal concerns, or link to activities that already possess cognitive legitimacy [66].

Suchman [178] further theorises that building new legitimacy and trust is different from maintaining or repairing lost legitimacy and trust. For example, organisations applying a cognitive strategy to gain new legitimacy could try to popularise, professionalise, standardise or certify the new “model” they wish to introduce. Suggested strategies to maintain legitimacy include consultation and keeping in contact with the principals or to use simplified communication, with clear explanations suggested to repair lost legitimacy.

## Assessing if sustainability governance systems are good

Two underlying premises of this paper are that governance can be an effective tool to promote sustainability goals (Premise 5) and that the design of the system affects its ability to do so (Premise 6). We also assume that the opportunity to develop such systems is reliant on the existence of societal trust (Premise 4), especially among principals and the agent. If the system’s design leads to its success in achieving its goals, it is a question whether this coincides with “good governance” as described in the literature. To examine this question, we review the origin of the good governance concept and some of the most prominent examples of good governance indicators, and finally discuss the theoretical and practical challenges associated with the use of the concept to assess the quality of a governance system (Sect. "The history of the good governance concept and existing assessment frameworks"). Based on this, we introduce “good sustainability governance” as a concept, examine how consensus can be developed around its definition, and how the concept can be translated to an operational standard for assessment of the quality of specific sustainability governance systems (Sect. "Defining good sustainability governance and proposing an assessment framework"). We propose that a general structure of principles, criteria, indicators and verifiers (PCI&V) is useful for the latter. A principle can be understood as a fundamental truth or value that guides further reasoning or action; a criterion is a standard or rule by which a principle is determined to be fulfilled or in progress; an indicator is a variable that can be measured or assessed to infer the status or direction of development for a particular criterion; and a verifier is the data or information that are collected to assess an indicator value [184, 185].

The proposed structure includes three principles (P) based on a theorisation of the tripartite legitimacy concept of input (P1, see Sect. “Input legitimacy”), output (P2, see Sect. “Output legitimacy”), and throughput (P3, see Sect. "Throughput legitimacy") legitimacy. The proposed structure includes several criteria that are kept open-ended with no associated indicators or verifiers, with later participatory processes in mind for their identification. Finally, we link the proposed good sustainability governance principles and criteria to a model for adaptive governance; adaptive governance is proposed as a way to move forward in situations with imperfect knowledge, uncertainty lack of predictability, and trade-offs.

### The history of the good governance concept and existing assessment frameworks

The good governance concept emerged in the late 1980s in the context of western foreign aid to developing countries. Under the International Monetary Fund (IMF), the World Bank and individual western aid donors, attempts were made to make developing countries adopt so-called structural adjustments to governance in return for loans [186]. The underlying understanding was that further economic development was limited by a governance crisis. By 1992, the World Bank Group introduced the good governance concept in their report *Governance and Development* [187].

Apart from the administrative and managerial meaning of good governance applied by financial institutions, the good governance concept occasionally includes democracy as a political element, which links good governance to the concept of legitimacy. Good governance may then be defined as “a state enjoying both legitimacy and authority, derived from a democratic mandate and built on the traditional liberal notion of a clear separation of legislative, executive and judicial powers” [186]. In other cases, the concept of good governance also includes human rights as a more substantive component.

One of the first and most widely acknowledged indicator frameworks for good governance was thus developed in a World Bank research program starting in the mid-1990s [188]. Their Worldwide Governance Indicators (WGI) were designed to assess the administrative and managerial qualities of governments [189]. The framework has six dimensions or aggregate indicators that are each composed of several sub-indicators: voice and accountability, political stability and absence of violence, government effectiveness, regulatory quality, rule of law, and control of corruption (see Additional file 1: Table S1). The indicators were assessed the first time in 1996 and annually since 2002 for over 200 countries and territories, with the results published and available from the World Bank’s WGI website [188]. The WGIs are used by researchers to examine their relationship with development parameters, such as Gross Domestic Product (GDP), reduction of poverty, or promotion of equity [190]. For example, by reviewing the data on sub-indicators under the corruption dimension, together with data on sustainable development and genuine investment in 110 countries, Aidt [191] concludes that corruption negatively affects a country’s sustainable development and erodes its capital base.

The WGI framework and the concept of good governance are not without criticisms. From a theoretical point of view, they have been claimed to be too broad in scope as the ambition to investigate an excessive number of issues may lead to no clear results on any of them [192]. The concept has also been criticized for being too functional, leading to its conceptual circularity as the indicators have been chosen to correlate directly with economic growth [192, 193]. This is illustrated by a sequence of rhetorical questions included in Rothstein and Teorell [192] from an article by *The Economist* (June 2, 2005) [194]: “What is required for growth? Good governance. And what counts as good governance? That which promotes growth.” A related criticism is its lack of suitability to address many non-economic relevant goals of governance such as high societal trust and perceived happiness and well-being [192]. Third, the data used to assess the WGIs are largely based on perceptions, which may cause a bias if the people surveyed hold prejudices toward how their country performs.

Addressing government specifically, Rothstein [193] and Rothstein and Teorell [192] suggest that the good governance concept be replaced with “quality of government” and that the quality of a government should be estimated based on the extent to which a government can implement and enforce laws and policies impartially, i.e. without taking into consideration anything about the citizen or the case that is not beforehand stipulated in the policy or law. This suggestion could be expanded from government to governance more broadly, suggesting replacement of good governance with quality of governance. Rothstein [193] argues that impartiality captures quality of government in a universal manner that will be acceptable to a wide range of religious, moral or philosophical doctrines, meaning that it has a stronger theoretical and normative underpinning, compared to competing concepts such as democracy, rule of law, efficiency and effectiveness [192]. He also argues that impartiality will not lead to a circular reasoning and finds that the concept shows better correlation with economic growth, life satisfaction and trust in institutions compared to the WGIs. He recognises, however, that there are exceptions such as China, which shows good progress on the same parameters, even if the quality of government score is low [193, 195]. Rothstein [195] infers that stringency in following the rule of law in this case is replaced by dedication to a political doctrine, which creates a government that is suited for effectively implementing policies for economic and social development. Others judge, however, that such pursuit of utilitarian legitimacy entails great risks of losing overall legitimacy [196].

The creators of the WGI framework have responded to the criticisms with clarification of its strengths and weaknesses [197–200], and similar frameworks continue to be developed by intergovernmental organisations. For example, the UN Economic and Social Commission for Asia and the Pacific published a good governance framework containing the eight principles [135] and the Council of Europe published the European Label of Governance Excellence (ELoGE) consisting of twelve principles for “good democratic governance” [201]. In the academic literature, good governance frameworks have also been developed for the advisory [202] or scientific testing of hypotheses on relationships between indicators of good governance and people’s trust in a particular governance regime. These frameworks generally include the principles of effectiveness, transparency, and accountability [203] (Additional file 1: Table S1).

Despite the criticisms, the UN has continued to utilise good governance as a means to achieve the MDGs, adopted in 2000 [204]. With adoption of the SDGs in 2015, however, the UN began to recognise good governance as a strategy rather than a goal, seeing it as a means to promote state and non-state actors working together in multi-stakeholder partnerships and networks to solve global challenges [205]. At the same time, the debate on the theoretical underpinning of the concept appears to have shifted to more pragmatic implications of the term. Gisselquist [206] argues that in the search for a proper definition of good governance, it is less important to ascertain the theoretical fit or avoid descriptive complexity, but rather, focus on ascertaining sound concept formulation, content validity, reliability, replicability, robustness, and relevance to the underlying research questions.

Gisselquist [206] furthermore recommends caution with comparisons made across studies that assess good governance due to the diversity of the contents and approaches of existing frameworks. Examples of diversity include most of the existing good governance frameworks, which focus on procedural contents, as compared to the ELoGE framework, which includes substantive contents, such as sustainability, human rights and financial soundness [201]. Furthermore, assessment frameworks may use the same words and terms to define the principles, criteria and indicators of good governance, but have different meanings for the terms. For example, Bennet and Satterfield [203] list “efficiency” and “accountability” as indicators under the category “effectiveness”, while in other frameworks these terms occur as side-listed categories (Additional file 1: Table S1). Even further, different data may be used to quantify and assess otherwise identical good governance indicators, resulting in risks that conclusions about differences arise from methodology and data choices rather than the tested matter [206].

Frameworks for good governance have also been developed within the private sector, in particular, by the Sustainability Standards Movement [207], when emerging Standard Development Organizations (SDOs) were seeking for practical and scientific knowledge that could guide them in “strengthening and promoting credible and accessible voluntary standards as effective policy instruments and market mechanisms to bring about social and environmental change” [207, 208]. Currently, the main organising agent of the movement is the International Social and Environmental Accreditation and Labelling Alliance (ISEAL), which was officially launched in 2002. ISEAL organises their framework under three Codes of Good Practice for social and environmental standards, including codes for standard setting [209], assessment of impacts [210], and assuring compliance [211].

Compared to the WGIs, the ISEAL codes were developed in a process that brought in concerns and experiences from ISEAL’s eight founding SDOs, that, for example, came from practical challenges experienced when trying to audit and certify farmers and firms [208]. The codes were developed in an iterative and interactive process involving an even broader range of stakeholders, including full, associate and affiliate ISEAL members, non-members, and various organisations representing the public, private and NGO sectors. The ISEAL standards have been tested for practical purposes, with auditors expressing that it has helped to solve problems with their own standards or certification systems [208]. The approaches of the World Bank and ISEAL thus represent opposite approaches to the work of good governance: a scientific and technocratic approach under the former, and an experience-based and participatory approach under the latter.

Gisselquist [206] questions if the WGI and similar frameworks will be considered as legitimate by those assessed for compliance. She suggests assessments conducted by impartial observers, with full transparency around methods and data to allow for replication, will likely reach a higher degree of legitimacy, as will systems with stakeholder participation. It has even been found that a poorly designed law can be perceived as more legitimate than a well-designed law if it is more meaningful in the context, making people feeling more motivated to act in agreement with its intent [212]. However, this question falls outside Premise 5 regarding the relationship between effectiveness of a governance scheme and its design. The assessment of private voluntary standard schemes against the ISEAL codes of good practice is voluntary, which makes it more likely that it holds a high degree of legitimacy among those choosing to be assessed. However, to the knowledge of the authors, there has been no rigorous scientific testing of the ISEAL codes to show how they are linked to perceptions of legitimacy or measures of impact. The technocratic and the participatory approaches might benefit from inspiration from each other, while still acknowledging that they were created for two different purposes, assessing the quality of government and assessing the quality of voluntary sustainability standards, respectively.

### Defining good sustainability governance and proposing an assessment framework

The great variety of approaches used to define good governance and assessing the quality of governance, i.e. its level of “goodness”, calls for more discussion and finding common ground to ease comparisons and communicate effectively about the topic (Premise 8). An informative parallel example exists in Sustainable Forest Management (SFM), which between 1992 and the early 2000s, went from being a virtuous concept [213] to one that was translated for operational use at the governmental and enterprise levels. The translation occurred as a result of intergovernmental collaboration [214, 215] and private initiatives, such as the FSC, Sustainable Forestry Initiative (SFI), and the Programme for the Endorsement of Forest Certification (PEFC), respectively. In the case of SFM, the intent of high-level principles was largely in agreement by interested parties, but there was disagreement related to the criteria and indicators (C&I). To some degree, however, consensus on C&I in SFM has been reached over time through repeated revisions of the PCI&V frameworks with more or less deliberate benchmarking as part of the input. At the level of C&I there is still a need for flexibility for meaningful implementation at national or sub-national levels [216–218].

In the context of sustainability governance, we propose that finding common ground might be possible with an open-ended definition and a structure for assessing “good sustainability governance” at the level of principles and criteria based on literature on “good governance”, including existing frameworks (Sect. “The history of the good governance concept and existing assessment frameworks”, Additional file 1: Table S1), the definition of sustainability in this paper, and the experiences from the development of the SFM concept. We thus propose to define good sustainability governance as an aspirational goal to be pursued through continual improvement of the governance system design, including not only standards, but also its implementation and enforcement systems, with improvements being driven by decision processes that are informed by science, monitoring and evaluation results and stakeholders’ practical experiences. This definition is inclusive of public governance, non-state market driven initiatives and other types of systems and initiatives, operating with different scopes and at different scales and levels of formalisation. The understanding of what is good or high quality in terms of sustainability governance will thus be defined by the politically legitimate entity [219] that identifies and prioritises the PCI&V by which the quality of the sustainability governance system should be assessed. The ultimate aim is that the system continuously improves and adapts to be effective and legitimate. The political entity may be a single or hybrid-agent who receives its legitimacy from relevant societies, communities or groups of stakeholders, or they may all directly be part of the entity. This understanding of good sustainability governance thus implies a value-based system with no single global optimal or true translation, but one that allows consideration of the natural and socio-economic context, as also recommended in the literature [164]. Grindle [220, 221] thus suggests that any elaborations must be seen as a “good enough” solution, indicating also expected upper boundaries for what can be achieved through sustainability governance.

In spite of this open-ended definition, more agreement is useful for operational PCI&V standards, at least at the level of principles. We venture to propose such common principles, as well as more open-ended criteria, based on the same sources as the definition (Table 7). According to UNDP [204], actors ascertain whether governance is good by looking at “the mechanisms that promote it, the processes used to govern, and the outcomes achieved.” We consolidate this understanding of the legitimisation process taking a starting point in the dual concept of legitimacy that was theorized in 1970 by Fritz W. Scharpf. He makes an often-cited distinction between input and output legitimacy [2]. In the context of European decision-making processes, he summarizes input legitimacy as “government by the people” and output legitimacy as “government for the people” [222]. In more recent literature, part of what was contained within input legitimacy is separated and placed under the newer concept of throughput legitimacy, which can be summarized as quality of the governance processes “with people” [223]. Despite the inconsistency in the issues that are included under each component of the tripartite legitimacy concept, the concept is well suited to contain the intents of the good governance principles that have been proposed in the literature. Our review of selected good governance frameworks suggests that several intents are common, especially principles expressed with concepts as justice, impartiality, comprehensiveness and balance of representation, responsiveness, inclusiveness, effectiveness and efficiency (Additional file 1: Table S1). We structured and expanded these concepts into a set of three principles and a list of associated open-ended criteria that is non-exhaustive (Table 7). Further arguments underpinning the structure are given in Sects. “Input legitimacy”, “Output legitimacy”, “Throughput legitimacy” and “Adaptive sustainability governance systems”.

**Table 7:** Principles (P, italics) and open-ended criteria (C, short name in italics) for good sustainability governance, which can be elaborated to define CI&V for assessment of the quality of sustainability governance systems

*P1. Seeking high levels of input legitimacy, also known as “political legitimacy” or “governance by people”. High quality of citizens’ political participation in governance systems and the governance system’s responsiveness to their inputs*	
C1.1	*Context and participatory approach:* Take time and make the effort to fully understand who the citizens or stakeholders are, for example, their interests and concerns, as a basis for deciding on the appropriate type and design of participatory approach for making decisions about the goals and the design of the governance system
C1.2	*Participation*: Establish principles or rules for legitimate participation in decision-making based on qualifying concepts, which can, for example, be democracy, balance of power, voice, inclusiveness, equality and equity of representation, which must be further specified
C1.3	*Early involvement*: Involve stakeholders at an early stage in the formation of the sustainability governance system, for example, to develop shared understanding of the challenges and consensus around the sustainability goals, including the level of standard strength and the procedural rules, based on the most relevant sources of scientific knowledge and knowledge about the context
C1.4	*Communication and mutual learning:* Create opportunities for continuous education of and communication between stakeholders to allow for exchange of experiences, mutual learning and possibly co-production of outcomes, if relevant
C1.5	*Monitoring, evaluation and adaptivity*: Gather data on stakeholder satisfaction regarding their involvement in decision-making and allow for engagement to discuss these data. Adjust the design of the engagement strategy if the level of satisfaction is inadequate
C1.6	*Responsiveness and openness:* Be open to innovation and change and make every participant’s contribution valued, with fairness in opportunities to contribute, and managing power dynamics if needed to achieve this
*P2. Seeking high levels of output legitimacy, also known as “performance legitimacy” or “governance for people”. High quality of performance which encompasses policy efficacy and effectiveness, and thus achieving the intended goals or making progress towards them*	
C2.1	*Context and policy design*: Take time and effort to fully understand the biophysical, social, economic and institutional context of the sustainability challenges, to increase the probability that the sustainability governance system design suits the conditions
C2.2	*Capacity and degree of institutionalisation*: Match the capacity and degree of institutionalisation of the governance system with its ambitions
C2.3	*Implementation and enforcement:* Design implementation and enforcement systems to achieve efficacy, effectiveness, and efficiency, considering the context
C2.4	*Monitoring, evaluation and adaptivity:* Measure, monitor and evaluate the governance system’s ability to effectively achieve its goal or make progress towards them, based on output, outcome, or impact indicators. Adjust the policies and standards if the performance is inadequate, and the monitoring and evaluation (M&E) system if it does not capture relevant developments, for example, new concerns, or provide evidence as intended
C2.5	*Efficacy and effectiveness:* Process the gathered data into information and knowledge that establish the evidence of the governance system’s efficacy and effectiveness in achieving the intended goals or making progress towards them
C2.6	*Efficiency:* All participants use limited resources efficiently to achieve the desired level of performance, i.e. optimise performance per used unit of resource, for example, costs, time, and administrational efforts. Specific means to achieve efficiency include collaborating with other governance systems (such as through mutual recognition) and actively identifying new technology that can improve efficiency of monitoring, auditing, and information transfer down through the supply chain
*P3. Seeking high levels of throughput legitimacy, also known as “procedural legitimacy” or “governance with people”. High quality of the system’s conduct in implementation and enforcement*	
C3.1	*Fairness in conduct*: Procedures are implemented and enforced according to rules that feel fair to all. Fairness may be summarized with concepts as impartiality, neutrality, rule of law, justice, and accountability in how the rules are enforced, with no knowledge assumed about the person abiding the rules. Include a mechanism for resolving conflicts
C3.2	*Truthfulness and transparency:* Unfalsified, un-manipulated, updated, transparent and easily accessible documentation of principles for participation, decision-making processes, resource use efficiency and performance, including data, information and knowledge and transfer of documentation through the supply chain
C3.3	*Absence of negatives*: No corruption, nepotism, structural racism, sexism, or arbitrariness in decision making and communication or in conduct of implementation and enforcement activities

The contents are based on sources given in Additional file 1: Table S1 [135, 173, 188, 193, 201–203, 224–226] and underpinning arguments and references given in the main text. The P&C are ideally embedded in an adaptive governance framework (Fig. 8)

### Input legitimacy

This section defines input legitimacy and describes the a

mbitions for achieving it from the viewpoint of a sustainability governance system. Given that stakeholder participation is at the core of input legitimacy, we proceed to address what good sustainability governance means in terms of stakeholder participation. We review literature on how different stakeholder participation governance system design features may influence the quality of the participation and help to generate legitimacy and trust, as reflected in the structure proposed in Table 7.

#### Defining input legitimacy and level of ambitions

Input legitimacy concerns the processes that inform the development and operation of governance systems and the degree to which these processes conform to the system’s procedural demands [225, 227, 228]. It was later proposed to distribute the issue of procedural demands to the throughput legitimacy concept, see Sect. "Throughput legitimacy". Input legitimacy is commonly described as based on balance of power, representation and the right to be heard (voice) where decisions are made [223, 227, 228]. In the context of government, input legitimacy has also been understood and assessed from officials’ experiences with the extent of negative incidences, such as bribing, nepotism, or success in lobbying [193].

Strategies for involving stakeholders may strive towards different levels of ambitions. Approaches aiming at changing the behaviour of stakeholders to achieve strategic government policy or business goals may be termed instrumental or pragmatic strategies [173]. The focus of such an instrumental strategy can, for example, be avoidance of counteractive boycotts and campaigning, with legitimacy seen as achieved when behaviours are law-abiding, or when an activity has been accepted (Fig. 4). Achieving trust in government or psychological identification with a firm, however, is beyond the scope of an instrumental strategy. For this purpose, a normative strategy is more likely to be successful. Normative approaches focus on stakeholders’ empowerment through their involvement in the decision-making [173] as a way to increase the credibility around the agency as a basis for granting trust (Fig. 4). Normative approaches are at the same time likely to achieve instrumental goals.

#### Criteria for quality of the stakeholder participation

There is general belief that good governance and gaining legitimacy is inextricably linked to effectively engaging with stakeholders. For example, it is compulsory to document stakeholder engagement when reporting under the Global Reporting Indicator (GRI) guidelines [229], a well-recognised CSR tool among large European companies [230]. Stakeholder involvement is also a fundamental requirement throughout the establishment, operation and improvement of standards and certification systems, see for example the three ISEAL Codes of Good Practice [209–211] and the WWF Certification Assessment Tool [231]. Evidence of the link between participation and granting of legitimacy is not comprehensive [230], but is emerging with evidence for example from water management [233].

Through an engagement process, stakeholders may articulate concerns and interests [234], provide on-the-ground perspectives in relation to policy implementation, and offer new and diverse sources of information [235]. Engagement processes are at the same time intended to contribute to maintaining appropriate levels of transparency for continuous betterment of the system. Continuous interaction between the principal and the agent may thus help to ensure that the agent is continuously aware of the principals’ possibly changing interests, and principals are better able to continuously make sure that the agent acts on their behalf without conflict of interest [147]. High levels of stakeholder engagement are thus assumed to result in more responsive, effective and legitimate governance systems, as compared to top-down models.

To the extent a linkage can be consolidated between a set of procedural rules for stakeholder participation and stakeholders’ confirmation that the governance is perceived as good, a system’s quality, in terms of input legitimacy, can be assessed indirectly from the contents of the procedural standards [223, 227, 228]. It is challenging, however, to consolidate the positive experiences and assumptions with rigorous science and the challenges are larger for normative compared to instrumental approaches [173]. However, based on a theoretical model derived from the scientific literature, Reed et al. [173] find that stakeholders’ early involvement and equal opportunities to contribute to the formation of the governance system were some of the most important factors to increase the likelihood that their participation lead to granting of legitimacy and trust. Tufte and Meflopulos [236] additionally find that early participation and continuous involvement through every stage of the governance system formation and operation increases the likelihood of arriving at an accurate and high-quality program, suggesting that high levels of input legitimacy leads to high levels of output legitimacy. The generality of such relationships are, however, questioned by Newig [232].

Consideration of context and scale of the involvement was another important factor to the successfulness of the participation [173]. This is in agreement with Wondolleck and Yaffee [237], who find that stakeholders may face fatigue when engagement processes require large participatory input, and with Hoffman and Lutz [238], who find that smaller stakeholders often find the costs of participation prohibitively high and therefore do not participate particularly in developing countries. Evidence of a similar situation is provided by Bennett et al. [239], who find that support for biodiversity conservation measures are more closely correlated with provisioning of education and knowledge to stakeholders and consideration of their interests in transparent decision-making, for example, their rights, livelihoods, traditional knowledge, and culture. Hence, when governance is considered fair and responsive, the need for participation and being heard in deliberate processes may be less important.

Stakeholder engagement processes thus tends to involve the most advanced actors with high capacity and power in society [228]. When smaller stakeholders are relatively homogenous, they may form alliances to increase their power and voice [138, 240] but they are still less likely to be organised with adequate capacity and competences for fully engaging [241]. This may force them to use their resources strategically for a limited number of topics. Also, relatively quick rotations of employees and volunteers in organisations with less capacity can lead to fluctuating levels of engagement and competences. These challenges underpin the need to tailor scale, duration, intensity and the level of formality of stakeholder participation processes to the situation.

Representation and equal opportunities to contribute is another criterion for high quality of participation [173]. Governance associated with global and international trade is especially challenged in this regard. In the case of biomass for bioenergy, the EU governs sustainability through co-regulation with approved private certification systems accepted as documentation for compliance with the EU RED I sustainability requirements [18, 19]. EU member state and parliamentary elections ensure democratic domestic representation, but criticism has been raised that there is democratic deficiency with regard to the influence and voice of impacted countries outside the EU, including developing countries. Stakeholders from these countries may have some influence through the certification scheme memberships and the participatory processes of these schemes, but policies and contents have already been laid down in the legislative frameworks of the EU. The only way to fully address this criticism seems to be through the establishment of a global governance regime for relevant environmental and social issues.

Literature from the 1990s and 2000s argues that application of soft systems is a precursor for normative stakeholder participation approaches to successfully achieve their goal. Soft systems are spaces or platforms that are seen by stakeholders as legitimate for facilitating negotiation and learning. It is ideally a forum where stakeholders can share and mutually validate their understandings of the issue in focus in order to reach consensus [173], or they may exchange experiences for mutual learning. The establishment of the biogas sector in Denmark is an example of how stakeholders connected through communication platforms established by the government results in rapid learning from a joint body of experiences as well as a rapid development of the general sector [91]. Reed et al. [173] suggest that interactive participation in stakeholder analysis may beneficially be used in connection with such communication platforms as a way for stakeholders to learn about each other and as a basis for further constructing social realities through negotiation and social interactions. Social realities can be described as creations of the human mind that are relatively stable and founded on human agreement, but still have objective existence [242] and impact on human life and societies. Examples of powerful social realities are money or nations. An example from bioenergy could be carbon parity time if used as an eligibility criterion to quantify climate change impact of using biomass for energy. In line with this, Megdal et al. [233] find that stakeholder engagement is critical to developing a common understanding of the context as a prerequisite for making sound decisions about an activity. Soft systems may be instrumental in creating the needed level of responsiveness and openness to stakeholder input by the system.

Recent scholarship suggests that online media platforms may increase the logistic feasibility of broader participation by overcoming the challenges to costs and capacity for smaller actors, but until now studies have rarely gone beyond the focus of the stakeholder as a customer [238]. An exception is Fraussen and Halpin [243], who suggest that digital innovations provide few benefits to groups that apply more traditional legitimacy principles of membership, such as “representation”, with an example being farmers’ membership to a farmers’ association. Another traditional legitimacy principle is “solidarity”, with an example being membership to an environmental NGO by anyone who shares the values or issue positions that the environmental NGO may advocate for. In comparison, the authors conclude that digital innovations provide more benefits to groups that accept membership based on a “subscription” principle, meaning only low-threshold actions of engaging on social media by following the group’s account or subscribing to its newsletter. It is yet to be seen if these more recent principles for legitimate membership can be developed to address democratic deficiency in international governance, cf. concerns for countries outside the EU that are impacted by transnational EU regulation of biomass sustainability (Sect. "Governance as a tool to reconcile conflicting views over sustainability").

### Output legitimacy

This section defines output legitimacy and describes ambitions that may be held by a governance system in this regard. We review relevant literature on how different design features influence the level of performance and effectiveness, as these are parameters at the core of output legitimacy, and a basis for the good governance principles outlined in Table 7.

Recognising that knowledge about the relationship between design and effectiveness is scarce and uncertain, we suggest as McDermott et al. [79], that systematic analysis of policy requirements may provide insights and is a precondition for deeper analysis and understanding of what makes policies effective. We attempt to contribute by proposing frameworks for policy analysis based on the literature in this field and discuss how different policy styles may be related to a system’s effectiveness in achieving its goals. This also underpins the arguments for the proposed good sustainability governance principles for output legitimacy (Table 7).

#### Defining output legitimacy and level of ambition

Output legitimacy is concerned with performance, i.e. the efficacy of solutions, their effectiveness in problem-solving and making progress towards the governance system’s sustainability goals [223, 227, 244]. Regulatory effectiveness is commonly understood as the extent to which the regulated entities or people act in accordance with the law [212], while efficacy can be understood as its ability to fully achieve a goal. Quantification of effectiveness requires consideration of at least the following three factors [155, 245, 246]:

- Effectiveness of the rules to the problems at hand;
- The degree to which the rules have been implemented and enforced; and
- The total number of actors bound by the rules.

Concerning the effectiveness of the rules to the problems at hand, the literature distinguishes between three approaches depending on what is measured: output, outcome or impact effectiveness [2, 227, 247, 248]. De la Plaza Esteban [227] explains output as the actual activities taking place such as issued regulations or certificates, produced reports, conducted research, and organized meetings. Outcome is the changes in behaviour of the targeted communities or people, while impact is the tangible changes in the targeted problem areas, for example, in the form of economic, social or environmental impacts. According to Hogl et al. [2] all three approaches in this tripartite model are conceptualised in a rather positivist manner because it is assumed that performance can be evaluated against the goals. Effectiveness may also be viewed in a more constructivist manner, which is concerned with who decides what effective means, for whom something is effective, and under what conditions. This is well in line with the proposed definition of good sustainability governance, as it relies on a legitimate political entity for finding answers to such questions (Sect. "Defining good sustainability governance and proposing an assessment framework"). In the following, we assume that such issues have been clarified.

As for the degree to which the rules have been implemented and enforced and the number of actors bound by the rules, we link this to the efficacy, effectiveness and efficiency of the design of enforcement and assurance systems.

#### Criteria for the governance system effectiveness

It is desirable from practitioners and policy makers’ point of view to understand what the effectiveness is of alternative policies, regulations and standards. There is broad agreement, however, that a governance system’s capacity to provide evidence of their own effectiveness is often limited for both public law [79] and non-state market driven systems [249], even if these have greatly proliferated in the last decades. The increasing occurrence of multilevel governance regimes or co-existing regimes further complicates the matter, as also seen in the bioenergy sector, for which Naiki [250] concludes that it works fairly well, even if there is potential for improvement in areas such as fairness and accountability.

Since it is harder to provide evidence of impact than outcome and harder to provide evidence of outcome than output, it is common to focus on the output, for example, the size of the certified area or the number of certificates. A study by Szulecki et al. [248] investigates 46 transnational energy partnerships and uses the sum of the outputs as a measure of effectiveness. The authors found that regulatory effectiveness is significantly correlated with the degree of institutionalisation of the governing organisation. They exemplify minimal institutionalisation by a partnership that only conducts self-reporting and have a website, and high levels of institutionalisation with formal organisations or partnerships, that have their own staff, steering committees and secretariats. The level of institutionalisation is not the same as high quality of performance, but high levels of institutionalisation are likely linked to the rigour of overall governance system design [248]. Next to the degree of institutionalisation, the power of partners, as well as the type of internal organisation are well correlated with the output.

Several studies measure effectiveness of certification based on outcomes, i.e. changes in behaviour of the regulated parties. In non-state market driven systems, the outcome is typically measured by the number and type of Corrective Action Requests (CAR), which are actions an enterprise needs to take before they can receive or maintain certification [245]. Several studies have used this approach [251–264] and a review concludes that there is reasonable evidence that certification can result in environmental and social improvement, but it is hard to find consistent patterns for particular indicators [245].

Dwivedi et al. [140] provide an example of another outcome-based approach in a spatially explicit assessment of the aggregated implementation rates of Best Management Practices (BMP) in sourcing areas of mill operations in the state of Georgia in the US, which are certified to the SFI Fiber Sourcing (SFI-FS) standard. Information about BMP implementation was available from audits performed by wood consuming SFI-FS certified mills and from the Georgia Forestry Commission biennial state-wide surveys that track BMP implementation rates on recently, typically less than two-year-old, harvested sites. The authors found a mean BMP implementation rate of 92.3% in certified sourcing areas, which was significantly more than a mean implementation rate of 90.9% in non-certified sourcing areas. This study was carried out under the SFI Conservation Impact Projects effort that began its work in 2015. The program aims to support projects that examine whether certification makes a difference on the ground [265], to understand more deeply if, how, and why the certification system leads to on-the-ground impacts, and to ultimately change the design if the impact is not as desired.

Several methods have emerged to assess the direct impact on the ground [66]. Elbakidze et al. [266], for example, measure habitat area set aside for biodiversity, habitat network functionality and modelled habitat connectivity on FSC certified land in Sweden and Russia to see how certification contributes to biodiversity conservation goals. The study remains inconclusive as the assessed parameters depended on a number of other factors than certification, such as the forest utilisation history, formal ambitions of the country and standard contents at the level of indicators. They suggest adaptive management and monitoring tools are needed to provide the evidence. In yet another study measuring on-the-ground impacts, Kalonga et al. [267] compared tree species richness, diversity and density on FSC certified community forests, non-certified open access forests, and non-certified state forest reserves, and find that registered biodiversity conservation indicators were positively correlated to certification. Novel technologies such as camera installations on the ground, on unmanned aerial vehicle, or mounted on harvesting equipment, aerial photo monitoring, LIDAR and remote sensing may increasingly provide opportunities for direct measurements of on-the-ground impact in the future. These technologies are increasingly possible and affordable, with the currently most common applications being registration of deforestation [268], harvesting rates [129] aboveground biomass [269], or soil disturbance [270]. Lopatin et al. [271] already found that remote sensing data could reliably verify 18% of the requirements of the PEFC forest management standard in Finland. However, remote sensing data still require better attributional data that have been verified with on-the-ground measurements and observations.

Perceived impact is also sometimes used to measure effectiveness, for example, in a global survey, where 85–96% of 27 respondents knowledgeable of forest certification systems perceived these are effective for issues such as traceability, biodiversity conservation, maintenance of soil and water quality, and social values, while only 10–14% thought these systems are effective in providing GHG emission and energy savings [148]. Conversely, 85–86% of 37–41 respondents knowledgeable of bioenergy certification schemes perceived these as effective with regard to traceability and GHG emissions savings, while 62–79% thought they are effective also for several other sustainability issues. An inherent weakness of this surveying method is respondents’ possible prejudices about how a particular certification system performs. Perceptions seemed to correlate with well-known differences in standard contents of forest and bioenergy certification systems, respectively, with perceptions likely portraying knowledge of standard contents rather than observations of on-the-ground impacts.

Even if the number of studies on effectiveness and impact has increased since the mid-2000s and the rigour of study designs has improved, the Steering Committee of the State-of-Knowledge Assessment of Standards and Certification [245] conclude that results from existing studies remain variable and are often not comparable, making it difficult to generalise conclusions. Knowledge about impacts of standards and certification is also still only addressing short-term impacts in a limited number of conditions [245]. Additionally, unintended impacts of certification have been poorly studied even if Pattberg and Widerberg [249] suggest that such impacts might be relatively abundant.

#### Criteria for the governance system efficiency

The efficiency concept focuses on avoiding the unnecessary use of limited resources as a result of regulatory activities, focusing on costs, time, resources and administrative efforts required to be and document compliance, as well as the efficient functioning of the governance system and organisation itself.

The governance system will eventually have to make decisions about standard and systems design with associated implications for the resource use. Such considerations may influence the number of goals a governance system chooses to pursue in its standards, how fast it requires regulated entities move towards the goals, and the methods used to monitor and evaluate the performance with implications for the quality of the evidence. In an assessment of forest certification impacts by Savcor Indufor Oy [244], efficiency was measured as the ratio between the quantity of output, outcome or impact, depending on the indicator, and the input of resources used to generate such performance. We suggest this is a useful measure for further work to examine efficiency as a criterion for good sustainability governance.

**Fig. 7:**
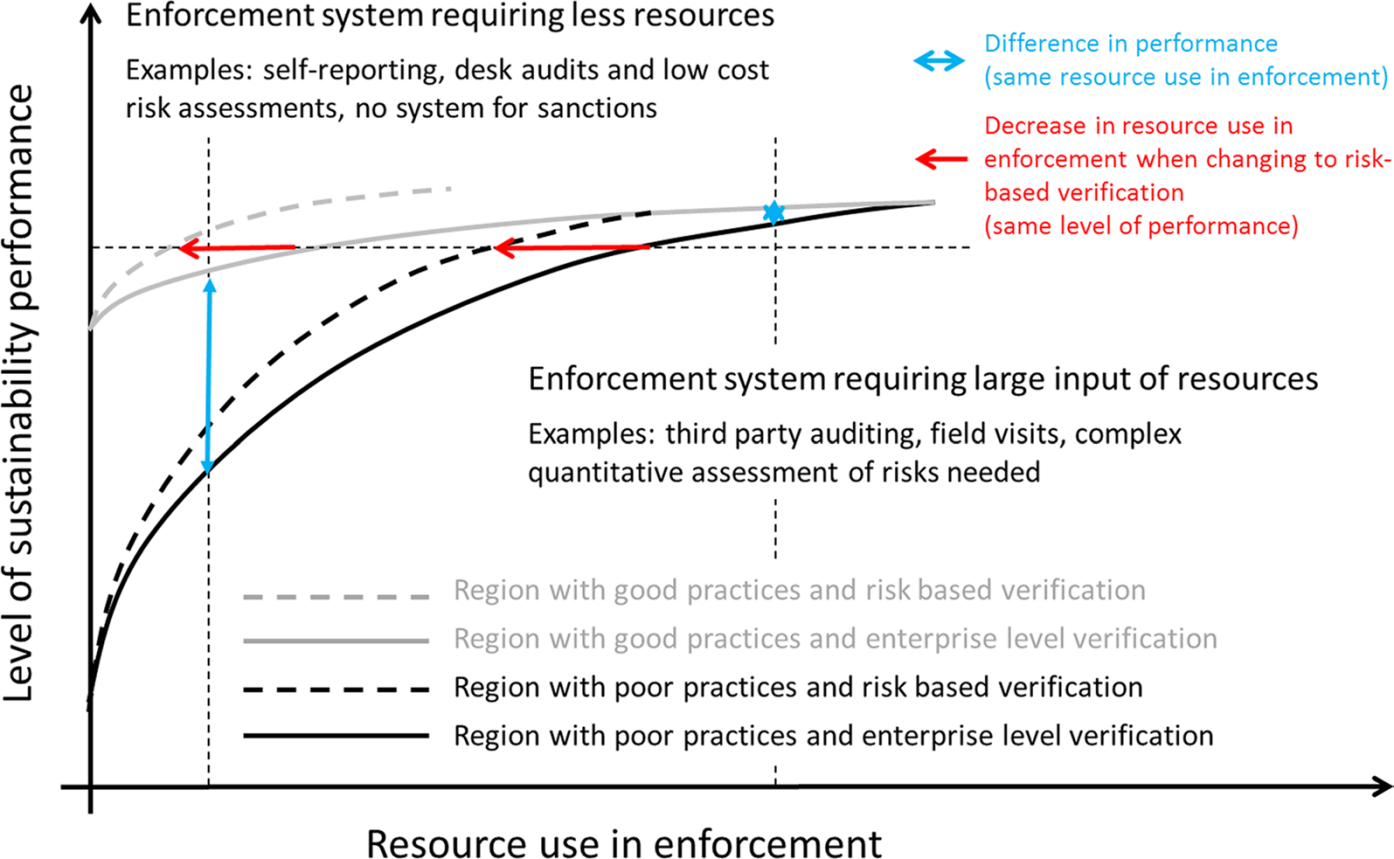
A proposed theoretical relationship between resource usage required for enforcement of a governance system and the level of performance that can be achieved in a region with good practices and high levels of trust and a region with poor practices and a low level of trust, for enterprise level and risk-based verification, respectively

The possibilities to keep the use of resources low for a certain level of performance will be site dependent [266], but the nature of the relationship has not been explored. It will likely depend on the extent to which the desired behaviours are already compliant without governance. We propose a theoretical relationship between effectiveness and cost efficiency that differs for locations where existing practices are good and poor, respectively (Fig. 7). Resource intensive systems will have a high level of performance in both situations. Less resource intensive systems may uphold performance in regions with good practices but are less likely to do so in regions with poor practices. This means that a higher efficiency can be achieved in locations that already have good practices compared to regions with poor practices.

Risk-based approaches are increasingly seen as a way to increase efficiency and achieve the same level of performance with lower resource usage (Fig. 7). This approach entails targeting enforcement resources and activities to the sites or values of greatest risks to the desired goals, thereby allowing the best use of limited resources [272]. Risk-based approaches are already widely applied both in public and private governance systems [272, 273] with several examples also for systems relevant to forestry and bioenergy including public regulation such as the EU Timber Regulation (EU TR), EU RED II and EU member state systems for sustainable solid biomass. Other examples include non-state market driven systems such as FSC Controlled Wood, PEFC Chain of Custody for Controversial Sources and the Sustainable Biomass Program (SBP) [61, 62, 170]. These example systems include a formalised risk assessment that must be conducted in accordance with written procedures and may be subject to public scrutiny and third party auditing before deciding where to focus the enforcement resources. Other types of enforcement systems require judgement of risk by the auditor or an inspector, with or without formal instructions [274, 275]. In this sense, most enforcement systems involve a risk-based element even if it may not be formal or explicitly expressed [62]. For example, in forest management unit level certification, audits are subject to time limitations and priorities made by the auditor on which criteria should receive most attention (Additional file 1: Table S2).

Rothstein et al. [272] contend that risk-based approaches lead not only to benefits but also embed challenges that are epistemic, institutional and normative in nature. Science is not always sufficiently advanced to provide answers about risks, and institutions do not always hold adequate resources and competences to establish credible processes to assess these. It may also be hard to agree internally on decision-making philosophies about risk with a subsequent need to risk manage the risk-based system [272]. As mentioned earlier, auditors or inspectors may hold considerable levels of discretion, with institutional credibility at risk, unless the quality of their conduct is high and consistent with institutional strategies [274]. Such consistence can be addressed through detailed guidance, special education or requirements of comprehensive experience. Finally, it can be a major challenge to legitimacy if there is only little normative agreement between institutional decisions and stakeholders’ perceptions about risk. Little is known about how the exact design of risk-based elements (see Additional file 1: Table S2) affects the trade-off between their effectiveness and efficiency in enforcement, but the increasing interest and usage suggests that there is an urgent need for more consolidated scientific knowledge [62].

Collaboration with other standard systems may also be a way to improve the overall efficiency of operating practices, for example, through higher levels of consistency among standards of different systems [226]. This is especially relevant in the context of bioenergy, which often relies on low-value feedstocks that are classified as waste from other production systems such as sawmill residues [276]. For such production systems, the technical and economic feasibility of sustainability documentation may entirely rely on the sustainability ambitions and associated governance systems of the broader industries, such as agriculture food supply chains, and forest-based timber or pulp and paper supply chains, for documentation of the sustainability of residual feedstocks [62, 245, 277]. Yet, there is little coordination between the bioenergy sector and the major value-creating industries. Existing systems for the larger sectors have rarely been developed to address major concerns of bioenergy sector stakeholders, for example, supply chain GHG emission savings and energy efficiency, that have not been a major concern to, for example, forest sector stakeholders [148]. This has resulted in additional layers of governance being added to the existing (Fig. 3). To the extent this has led to regulatory redundancy, lack of coordination, and contradictory requirements, it will have implications for efficiency and throughput legitimacy, generally. The challenge must be addressed across the involved sectors, possibly in the specific landscapes [138], as well as across jurisdictions for internationally traded products. The bioenergy sector, with its low-value production has few resources to drive such changes.

Agriculture and forestry sectors are increasingly attracting new industrial actors, most notably entrepreneurs that seek to supplement existing structures with innovative technologies for the circular bioeconomy. With their attention to unused residual resources, these actors are unlikely to disrupt the main forestry and agricultural industries, but their role as key actors in strategies to create more sustainable societies may hold the potential for a prominent role in setting direction and facilitating up-scaling of sustainability governance for any biobased sector. If larger shifts and changes take place in the forestry or agriculture sectors, this would also impact bioenergy supply chains and their existing sustainability governance systems.

Research on effectiveness and efficiency of governance systems has mostly been conducted at the production unit level, but the aggregated impact of these systems must also be considered [245]. Biermann et al. [278] emphasize, in the context of transnational governance, that there is a need to evaluate performance for the broader governance complex and not only scrutinize the performance for the individual certification system or governance element, separately. Research targeting the aggregated, cross sectoral and landscape level impacts of governance is emerging [138, 140], but challenges in terms of resources, rigorous methodology, or both, are pronounced.

##### Policy content and style as a basis for assessing rule effectiveness

McDermott et al. [79] suggest that the limited knowledge about the effectiveness of governance systems is partly due to lack of systematic analyses of what is required by policies. The authors see analyses of policy contents and styles as a platform from which questions about effectiveness can be better addressed. Frameworks for benchmarking of policies have been applied to provide structured knowledge about standards comparative substantive content, while classification systems for policies have been developed to provide structured knowledge about policy styles.

The WWF Certification Assessment Tool [231], for example, uses the concept of standard strength [231] to express the extent to which standards articulate a range of sustainability objectives. The WWF CAT consists of 80 indicators organized into eight categories: legality, tenure, use rights; community relations; workers’ rights; water and soil; biodiversity; pollution, waste and GHG emissions; planning and communication; and other good forestry practices. The tool is used to benchmark the FSC and PEFC international forest management standards [231]. Several other studies analyse and compare standard strength, using other benchmarking frameworks developed for the specific study. There is often a great overlap in benchmarking topics when standards are evaluated to see how they address sustainability of bioenergy, including topics such as biodiversity, carbon stocks, and soil or water quality [138, 216, 279–282].

McDermott et al. [79, 283] also develop classification systems for comparative analysis of forest policy styles with the “policy setting” as the unit for analysis. Keller et al. [284] create a similar system to analyze GHG policies of agri-food certification schemes. The policy setting is defined as specific on-the-ground requirements, as opposed to policy goals, which steers the overall policy development, or policy objectives, which are the specific aims being addressed by the policy [79, 283]. The policy setting is comprised of a set of policy variables, for example, biodiversity conservation, riparian zone design, or management of native forest, which links to standard strength. Combining the approaches by McDermott et al. [79, 283] and Keller et al. [283], we derive a classification system of two dimensions, “level of discretion” and “method”, taking three and four values, respectively, with their combinations resulting in a matrix of twelve policy styles (Table 8).

**Table 8:** Two-dimensional classification system for policy variables of policy settings in sustainability governance systems, with the first dimension being “level of discretion” and the other being “method”, making up twelve theoretical policy styles

Level of discretion → Method ↓	*Voluntary* Optional or recommended policies encouraging action	*Contingent* Requirements of specific actions are contingent	*Mandatory* Specific action required
*Procedural* Existence of management system or plan requirements (output based)	Management system or plan is recommended	A specified or non-specified management system or plan is required, in part or contingently	A specified management system or plan is required
*Implementation* Compliance with BMPs (outcome based, indirectly impact based)	Implementation of specified or self-developed BMPs is recommended	Implementation of specified or self-developed BMPs is required, in part or contingently	Specified BMPs must be implemented
*Progress* Progress demonstrated by measurement and monitoring (impact based)	Demonstration of progress compared to BAU or status quo by measurements and monitoring is recommended	Demonstration of progress compared to BAU or status quo by specified or non-specified measurements and monitoring is required, in part or contingently	Demonstration of progress compared to BAU or status quo by specified measurements and monitoring systems is required
*Substantive* Achievement of explicit on-the-ground goals or thresholds (impact based)	Achievement of specific goals or thresholds is recommended, perhaps with suggestions for actions	Achievement of specific goals or thresholds is required, in part or contingently, perhaps with specific suggestions for actions or method is flexible to own judgement of what actions are most effective and efficient	Specific goals or thresholds should be achieved through specific actions

See the text for further explanation of terms. The classification system was inspired by McDermott et al. [79] and Keller et al. [284]. *BMP* best management practices, *BAU* business as usual

The values taken by the level-of-discretion dimension includes “mandatory”, “contingent”, and “voluntary”, with mandatory rules requiring a specific course of action, and voluntary rules encouraging but not requiring a course of action [79, 283]. Contingent is intermediate between mandatory and voluntary rules, with requirements to be met under certain conditions such as temporary or seasonal requirements, or for some percentage of something, corresponding to “medium” in the framework by Keller et al. [284]. It can also be understood as a rule that varies depending on other jurisdictional regulations, in agreement with “contingent” as defined by McDermott et al. [285], or rules with a mandatory objective but flexibility and freedom to choose how the objective should be met, as described for Swedish forest legislation by Lindahl et al. [286]. The language of the policy setting may help discern the level of discretion. The subtle use of “may” versus “shall” or “must” is indicative of a voluntary versus mandatory policy, respectively [284]. Additionally, many exemptions to a rule or vague wording may result in it being more voluntary or contingent in nature.

The values of the method dimension are “procedural”, “implementation”, “progress”, and “substantive”. Substantive rules address on-the-ground practices, while procedural rules address the characteristics of the management system [79, 283]. An example of a substantive rule is a requirement not to exceed a specific maximum size of a forest clear-cut and an example of a procedural rule is the existence of a forest management plan.

Based on outcome effectiveness, i.e. measuring change in behaviours, the value implementation requires monitoring the extent to which BMPs are implemented for compliance of on-the-ground actions by land-owners or firms. BMPs can be understood as courses of action that “transform knowledge about local conditions and practices into prescriptions for low-impact operations” [287]. In certain situations, it may be required to develop one’s own BMPs if no existing BMPs apply. The BMPs may themselves be procedural or substantive in nature or a mix of these and may act as voluntary guidelines or mandated requirements. They are generally developed at national or local levels and are considered to be most effective when based on best available science, especially if this was conducted or validated for national or local conditions, depending on the scope of the BMPs [287]. To the extent that national and local level scientific knowledge is not available, local experiences and local expert knowledge often make valuable contributions to BMP development.

Based on impact effectiveness, progress as method requires continuous measurement of specific sustainability indicators as part of a formal monitoring and evaluation (M&E) system. The measurements can be compiled in databases that are used to monitor, analyze and evaluate progress as well as revise policy to better achieve the intended goals, cf. adaptive governance in Sect. "Adaptive sustainability governance systems".

The relationship between the policy style and regulatory effectiveness likely depends on the context. More regulation, may lead to a higher degree of progress towards sustainability goals or limit a trajectory of degradation. However, inflexible or mandatory policies applied to situations that are complex and site dependent may also lead to unintended undesired impacts or incentives [79], as, for example, experienced with large scale technocratic planning in tropical forestry in the 1970s [32]. This is likely the reason that a shift in regulatory focus from few simple to more complex policy goals in Swedish forest legislation was followed by a shift from easily comprehensible mandated, prescriptive rules to flexible approaches relying upon the competences of the local forest managers and owners for judgement of methods needed to achieve the goals [286]. To some extent, such deregulation processes are also taking place for forestry laws of the Baltic countries [288]. McDermott et al. [79] further note that more mandated and prescriptive regulations can also be a sign of lack of trust among key actors and sometimes also link to lack of effective enforcement. Prescriptive rules may also inhibit social learning, adaptivity of the management and building of trust in regulatory authorities or sustainability of business activities [79]. Hence, the choice between mandated prescriptive policies versus voluntary rules is not trivial.

##### Types of enforcement strategies as a basis for assessing degree of enforcement

The behavioural patterns or changes that the governance system intends to promote may occur voluntarily. As argued earlier, the chance of voluntarily compliance is higher when the rules are perceived as meaningful by the regulated entities. When behaviours are not compliant, it may be intentional or due to inherent difficulties. In both cases, an effective and cost-efficient enforcement system is necessary to gain or maintain trust, allowing high levels of trust to co-exist even in situations with high levels of suspicion (Table 3). A major challenge in design of enforcement systems is the trade-off between effectiveness and cost-efficiency. Constant monitoring and reporting of data is the most effective means of ensuring compliance but is very costly, and may be less needed where the level of normative agreement is high (Fig. 4).

Gunningham [274] reviews the literature on this topic and proposes the enforcement strategies that are most likely to be successful based on combinations of factors such as business size, level of impact that the regulated entity has on the problem that needs to be solved, the opportunities for regularity and depth of the contact between authorities and the regulated entity, and expected attitudes of the regulated entities. The author suggests that the chance of success of a given strategy will also differ depending whether the end goal is to only affect behaviour of the regulated entity or also attitude, and identify seven intervention strategies. The intervention strategies range from softer ones based on cooperation, dialogue and conciliation to more strict command and control approaches, with sanctions such as fines or prison. In between there are mixed strategies where sanctions are contingent on certain criteria such as the severity of the offense or the number and extent of previous violations.

The softer approaches are not generally found to be effective where used in isolation as they discourage compliant actors who see that offenders are not punished. Strict rules are also not effective in isolation where they create a culture of resistance or distrust against the rules. Strict rules can be effective, though, for actors that rationally calculate costs and benefits of compliance. No regulation is also sometimes recommended in situations with poor opportunities for contact, and where the entities are self-regulating through well-implemented, strategically aligned business plans or where actors care about their reputation and operate in markets with high levels of public scrutiny [274]. In those cases, the effectiveness may rely on private certification. We argue that there are no firm conclusions about the effectiveness of these systems nor are there firm conclusions on where certification systems failed to discipline offending companies due to vested financial interests [68]. Enforcement with frequent contact and a blend of persuasion and coercion has been found to create a collaborative environment that leads to changes in behaviour, rather than changes in attitude [274]. Finally, various risk-based approaches have, as mentioned, been proposed as a way to balance effectiveness and cost-efficiency [272], and systems with continuous monitoring and evaluation may have a better potential to achieve the intended impact [92] (see also Sect. "Adaptive sustainability governance systems").

### Throughput legitimacy

This section defines the concept of throughput legitimacy and reviews literature for the following two key throughput issues: fairness and truthfulness in regard to transparency of information (Table 7).

#### Defining throughput legitimacy

Bäckstrand [228] includes mechanisms for accountability, transparency of decisions, monitoring of effectiveness and sanctions as a second aspect of input legitimacy, besides participation and involvement, while Schmidt [223] and Schmidt and Wood [225] suggest adding throughput legitimacy as a third dimension to Scharpf’s theorisation to cover these topics. The term emerged in second half of the 2000s [289, 290] and in line with Scharpf’s summarisations of input legitimacy as “governance by people” and output legitimacy as “governance for people”, Schmidt [223] and Schmidt and Wood [225] summarise throughput legitimacy as the quality of the governance processes “with people”, and further describe it as the way in which the policy-making processes work to ensure the efficacy and fairness of governance, accountability in decision making and officials’ conduct, transparency of information and the inclusiveness, and openness to civil society.

The delimitation of the three legitimacy concepts is, however, ambiguous across the literature. In this paper, we keep inclusiveness and openness to consultation and accountability in decision making as aspects of input legitimacy. We keep the quality of the system’s conduct of implementation and enforcement as an aspect of throughput legitimacy principle, which includes the criteria of fairness and truthfulness with transparency of information (Table 7). The inclusion of the criteria of truthfulness is inspired by ISEAL [226], whose definition concerns that “claims and communications made by actors within standards systems and by certified entities about the benefits or impacts that derive from the system or from the purchase or use of a certified product or service are verifiable, not misleading, and enable an informed choice.” Hence, it addresses the credibility of the evidence of sustainability performance and its transfer along the whole supply chain. Transparency of such information is essential to communicate convincingly with people about the truthfulness of the evidence of the desired impact. Truthfulness and transparency are, however, also seen as critical to gaining credibility in input-related issues [226].

#### Criteria for governance system fairness

Fairness means implementing and enforcing the rules in way that can be seen as fair to all [225]. It may be captured in the concept of impartiality, which has been suggested as the theoretically strongest indicator of good governance overall [193]. A system is impartial when it maintains neutrality in how it treats its regulated entities or stakeholders [291] (cf. Sect. "The history of the good governance concept and existing assessment frameworks"). Inspired by the conceptualisation of the “rule of law” articulated by the Supreme Court of Canada, fairness can also be explained as a governance system free from arbitrariness, meaning that it must not arbitrarily exercise power. To maintain fairness, there must be the creation and maintenance of expressed rules intended to preserve and embody order in the application of the governance scheme that are clear, public, stable and applied evenly, for example, for those rules related to stakeholder engagement, monitoring, and enforcement [292, 293]. Other concepts related to fairness in how people are treated includes justice and accountability, and officials acting with integrity, credibility, trustworthiness, without bias and according to expected ethical and moral standards [225]. Each governance system needs to decide on the specific concepts that best serve increasing fairness in their case and make specifications on how it should be understood for practical purposes.

#### Criteria for governance system truthfulness

Evidence provided by M&E systems and experimental research form a basis for conveying information and knowledge for making credible claims about performance and sustainability risks [210]. Such evidence provides a profound basis for an agent’s effective communication with principals about the system’s performance, even if also important to adapt language and means of communication to the specific target group of principals.

The management literature makes an important point about distinguishing data from information, which is different from knowledge [294]. Data consist of facts that become meaningful through their combination and analysis into information. Information subsequently becomes knowledge when it is interpreted and discussed in a context or wider perspective. The concept of transparency is closely linked to truthfulness, as is accessibility, and both concepts can be interpreted in relation to the tripartite model with data, information and knowledge. If only data are publicly accessible there is a low level of transparency as it requires processing, resources and skills to understand what their meaning and the implications of them. There is also a low level of transparency when information and knowledge is publicly available but no data are available making it impossible to reproduce and check the truthfulness of the conveyed information and knowledge. Tuomi [294] additionally makes the point that data tend to emerge when information is available, and that subsequently, information tends to emerge where knowledge is available. Consideration of this reversed hierarchy suggests the importance of making priorities in establishing organisational memory, thereby creating higher levels of organisational flexibility and ability of renewal.

Governments and private organisations often report their sustainability policies and performance. For example, there is increasing sentiment on the part of the world’s largest investors that companies publicly disclose the risks posed to their economic activities from climate change. Specifically, there is movement for companies to disclose the physical, legal, technology, market, and reputational risks facing the company through the annual financial reports as mandated by regulators. The most prominent example of this is the Financial Stability Board’s Task Force on Climate Disclosure (TCFD), an intergovernmental initiative headed by Mark Carney, the former governor of the Bank of England, and Michael Bloomberg, a billionaire and former mayor of New York City. The TCFD, whose members consist of the world’s largest investment funds and insurance companies, published guidelines for the content of climate change disclosure as well as the processes [295]. At the same time, government regulators in Canada [296], the US [297] and Europe [298] have also published voluntary guidelines for climate change disclosure. These initiatives, however, are often limited to risks associated with climate change, and, therefore, do not wholly contribute to the goals of sustainability. Nonetheless, movement is being made towards broader sustainable disclosure for investors. In the bioenergy context, “Standards of Biomass Supply Chain Risk” (BSCR standards) are being elaborated to de-risk capital market investment in biomass projects [299]. The standards require disclosure of several issues, including environmental sustainability metrics in addition to those associated with climate change, for example, potential risks to wildlife, soil and water quality, water use, pesticide use, and related to use of genetically modified organisms (GMO).

For investment purposes, the collected data are processed into information and knowledge that is critical in decision making about investments, but in other situations, resources are not necessarily available to process the collected data into a form that is useable for public scrutiny. Characterisation of the type of transparency around data, information and knowledge and relevance of the scale of the data to the problems at hand (Additional file 1: Table S3) may act as a starting point for further examinations of how such features are perhaps linked to perceptions about the credibility and truthfulness of sustainability and performance claims.

Apart from truthfulness of the evidence, credible and unfalsified transfer of the documentation through the supply chain is also critical, especially where products are traded over longer distances, domestically from countryside to cities or internationally [138]. At least five supply chain control systems are used in practice, with varying levels of information disclosure, costs and risks of falsification (Table 9).

**Table 9:** Supply chain control systems for bioenergy feedstocks, with mixing and sustainability claim characteristics, and hypotheses about the benefits and challenges of each system, in terms of information transparency, costs and risks of falsification

Supply Chain Control System	Book and Claim	Mass Balance	Segregation	Full Segregation	Identity preserved
Level of mixing	Mixing	Controlled mixing	Controlled mixing	No mixing	No mixing
Sustainability claim	Supports the production of certified forest biomass (equivalent to xx % of the biomass utilised)	Supports the production of certified forest biomass (equivalent to xx % of the biomass utilised)	Contains minimum xx % of certified forest biomass	Only certified forest biomass	Only certified forest biomass with information about original producer
Information transparency	Low	Medium	Medium	High	Very high
Costs	Low	Medium	Medium	High	Very high
Risks of falsification	High	Medium	Medium	Low	Very low

Based on Stupak et al. [300]

The book and claim system allows producers of sustainable feedstock to convert supply into certificates, which are then placed on the market. The value of the certificates is sent to the producer, who gets the premium in exchange of commitment to environmental and social standards as required by the applied governance system. Supply chain control systems with mass balance determine the volumes in a supply chain that comply with the sustainability standards of the governance system, with a mechanism to verify that non-compliant material come from non-controversial sources. The percentage of compliant material may be disclosed in the claim.

Supply chain control system with segregation, full segregation and identity preserved all require that compliant and non-compliant materials are physically separated from production to sale. Full segregation allows no mixing of compliant and non-compliant material, while a system with segregation may allow some maximum amount of non-compliant, non-controversial sources. A system with identity preserved requires information about the original, individual producer is passed down through the supply chain.

The costs associated with each of these chain–of-custody systems typically increases from book and claim to segregation systems, and opposite for the risk of fraud (Table 9). As the bioenergy industry exists today, feedstocks come from multiple types of sources with sourcing regions that continuously change over time and feedstock coming with a range of different sustainability certificates as each actor often uses multiple systems [276]. This makes it a complex and demanding task to maintain transparency and communicate effectively about sustainability performance and claims.

New technologies for automated data and information transfer may contribute to control system decreased costs, increased harmonisation and consistency of the data flow, which may also contribute to increased transparency, efficiency, and, consequently, the credibility, and belief in truthfulness about the evidence and the claims. Drawbacks of such systems are that a company may lose privacy and control over internal data critical to its competitiveness.

Truthful data, information and knowledge (logos) are not only a basis for communication of historic performance and compliance. In the long term, these elements also provide a foundation for building reputation (ethos). When appeal to stakeholders through company appearance or pure authority (pathos), the linkage to performance data is even more critical, as this is where the risk of manipulation in communication is largest (Table 3).

Regardless of available and transparent truthful information, examples still exist of a decoupling between facts about performance and people’s perceptions of performance. This has, for example, been seen in relation to police professionalism and performance in the UK [301] and in relation to the health risk of coronavirus infections, with poor relationships between the real performance and risk and what was perceived by citizens [302]. If citizens or stakeholders do not react positively to the availability of truthful information about high levels of performance, with their granting of legitimacy and trust, it will be necessary to investigate what other factors shape public perceptions than facts [301] (Table 6).

### Adaptive sustainability governance systems

**Fig. 8:**
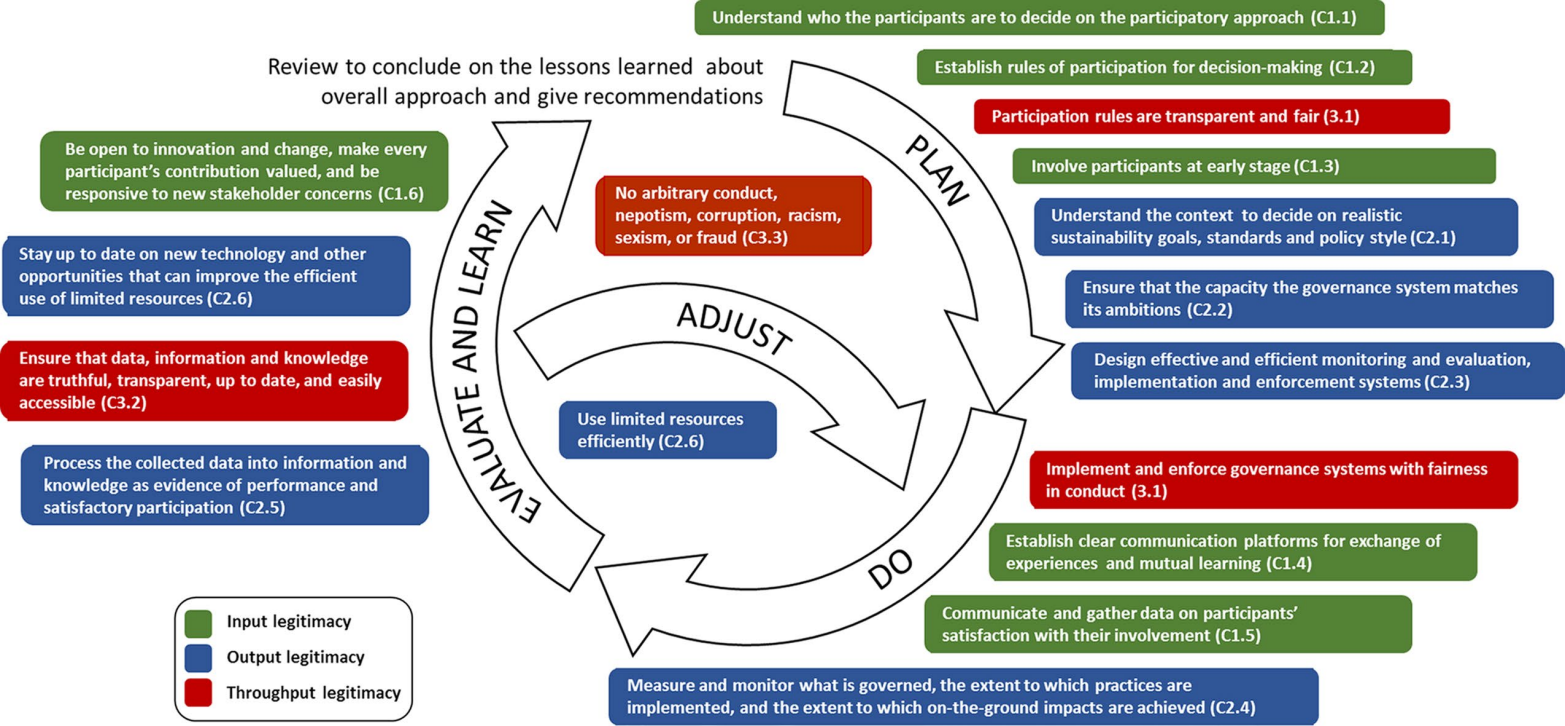
Model for adaptive sustainability governance with proposed linkages with the principles and open-ended criteria for good sustainable governance as enumerated in Table 7, organised sequentially

In this section, we explain why there is a need to supplement conventional ‘predict-and-act’ governance tools in with adaptive elements. Based on review of existing governance systems, we propose how governance systems can be classified with low or high levels of adaptivity with examples from a range of policy methods such as mandated laws and voluntary private certification systems (Table 8). Finally, existing Adaptive Forest Management (AFM) frameworks are used as a basis for proposing for an adaptive governance model (Fig. 8) that embeds the earlier proposed good sustainability governance principles and criteria (Table 7) in an adaptive setting.

#### The need for adaptive governance

Even if there are overall principles to be followed, it is hard to reach context specific conclusions about the best design of a sustainability governance system or regime. This creates the need for supplementary tools as an alternative to conventional ‘predict-and-act’ that enable making meaningful decisions in the presence of imperfect knowledge, poor levels of predictability, uncertainty and complexity, as an alternative to making decisions in the dark [3]. Such tools must be able to identify changes in political and stakeholder priorities, integrate new knowledge, and rapidly change direction if unintended impacts occur. The need for such tools was already recognised by the ecologist C.S. Holling and co-authors in the 1970s [303]. Combining knowledge about ecology with principles from system’s theory, the authors created a set of adaptive management principles that is now commonly known and institutionalised in forestry as the Adaptive Forest Management (AFM) concept. AFM integrates experimental research, management planning, monitoring and evaluation, and policy revision into a continuous process that encourages continual learning and adaptation [287]. Apart from adaptivity around practices, Malekpour and Newig [3] examine adaptivity in the stakeholder engagement processes, governance structures and in terms of available resources through a meta-analysis of 40 real-case applications of adaptive planning in a range of sectors. The authors conclude that adaptive planning is more effective when it is supported by dedicated governance structures that coordinate and lead negotiations, has long-term goals and investment strategies, and avoids solutions based on simplistic assumptions. Hence, adaptive management should not be separated from adaptive governance. This is in line with Rist et al. [304], who suggest that adaptive management is always embedded within a broader management, social, political, and institutional context.

Each governance system will have to make decisions about how priorities should be made, for example, if citizen or stakeholder involvement is prioritised over documentation of performance, or opposite. It is important to be aware that such choices may influence the extent to which legitimacy is granted to the system. Input and output legitimacy may allow trade-offs among them, where good performance can compensate lack of citizen and stakeholder participation, or vice versa [225], as we have seen in the case of China [196]. However, the opposite may also occur, where high levels of performance may be disregarded by low levels of participation leading to poor overall granting of authority or SLO due to some citizens’ or stakeholders’ dissatisfaction with their potential inability to adequately influence the government policies or business activities, respectively. In contrast, systems with high levels of citizen and stakeholder involvement may result in reduced ability to effectively achieve sustainability goals as inclusive decision-making processes may be too lengthy and inconclusive to effect real change [232, 305–309]. This may also jeopardize the granting of legitimacy. The experience is that a high level of throughput legitimacy cannot make up for poor quality of citizen or stakeholder participation or for poor performance, but in contrast, poor procedural quality can jeopardize people’s granting of input or output legitimacy [225]. Similar trade-offs and synergies may also occur among individual criteria under each legitimacy principle.

The current shift from public to increasing participation of private governance seems to result in a shift from an input-oriented towards an output-oriented legitimacy focus as private governance systems are often more driven by the ambition to offer solutions to specific environmental and social problems, compared to a wish to have people participate in an inclusive manner [77]. This has been put forward as a root cause in the criticism of systems governing sustainability of bioenergy (Sect. "The conflicting views over the sustainability"). An adaptive governance system can be a platform, from which such trade-offs can be made with the acceptance of the involved parties. It has thus been argued that less effective and efficient solutions must sometimes be chosen over better ones, because political needs or societal expectations require it, for example, for the enforcement to be perceived as impartial and fair [275].

#### Adaptive elements in existing governance systems

Governance systems with adaptive management elements exist in a variety of forms in both public and private regulation (Table 10). Examples include the Policy Framework for Sustainable Forests in Ontario, Canada, which captures the provincial commitment to SFM, as entrenched in the Crown Forest Sustainability Act (CFSA) [53]. The framework was adopted in 1994 together with the CFSA and it includes several “Stand and Site Guides” with site-specific on-the-ground operational guidance that are revised in regular 5-year cycles [53]. In a case study on adaptive management in relation to long-term soil productivity policies in Ontario, Morris et al. [310] explain that the publishing of peer-reviewed articles is a critical step in the adaptive management cycle, as it provides senior policy advisors and environmental NGOs with credible results on various elements. Further, the authors point out the importance of synthesis efforts and meta-analysis of these studies in the policy revision/implementation process in order to place regionally based results into a broader geographical context.

**Table 10:** Categorisation of governance systems according to the nature of their adaptive features

Method and involved agents	Low level of adaptivity	High level of adaptivity
	Systems without adaptive feature except law making processes with slow dynamics (standard change typically taking place over periods longer than 5 years)	Systems with adaptive features and fast dynamics (standard change typically taking place within a 1–5 year period)
Mandated public law	Law amendments, generally^a^	Law amendments, special cases^b^
Mandated hierarchical public systems	General mandated objectives or requirements^c^	State guidelines with site specific guidance^c^
Mandated surveillance reporting by public authorities	International conventions and agreements^d^	Public reporting to monitor changes that might potentially require changes to laws in a longer term^e^
Co-regulation with mandated public law and voluntary private systems to show compliance	General mandated public objectives or requirements, which may be contingent on subsidies^f^	Private certification systems with third party auditing as a basis for periodic assessment, to show compliance with public requirements^g^
Voluntary surveillance reporting by private actors	No examples available	Agreements between private partners with reporting of progress with third party audits as a basis for periodic assessment ^h^
Voluntary private systems	No examples available	Private certification systems with third party auditing as a basis for periodic assessment^i^ Landscape governance initiatives, most often without auditing^j^

^a^For example, Swedish forest law [287] and forest law for Crown land in Ontario [53, 313]

^b^For example, the German Renewable Energy Act (REA) [92]

^c^For example, the Policy Framework for Sustainable Forests in Ontario [53]

^d^For example, United Nations Framework Convention on Climate Change (UNFCCC), and the Convention on Biological Diversity (CBD).

^e^For example, EU RED I [18], and EU Recommendations on sustainability criteria for solid biomass [57]

^f^For example, EU RED I [18], EU RED II [19], SDE+ in the Netherlands and UK CPET [62]

^g^For example, systems approved under EU RED I: ISCC, Bonsucro, RTRS, RSB, 2BSvs, Red Tractor, SQC, r8. Red Cert, Better Biomass, RSPO, KZR INIG, Trade Assurance Scheme for Combinable Crops, Universal Feed Assurance Scheme, and SSAP [314]

^h^For example, the Danish Industry Agreement on sustainable wood chips and wood pellets [61]

^i^A proliferation of private certification systems, such as FSC, PEFC endorsed system, including SFI, and SBP, GGL, etc

^j^Examples from especially developing countries in Diaz-Chavez and van Dam [138].

Reviewing the literature on Ontario’s forest policy, it is clear that adaptive management is generally implemented through guidance rather than laws approved by elected officials, which is likely because laws cannot be easily changed as it requires the approval from the relevant legislature or parliament. Stability of forest law is also evident from an analysis of Swedish forest legislation through the 1900s; the first Forest Act was established in 1903 for privately owned forests, expanded to all forests in 1979, with more comprehensive revisions taking place in 1993 when goals were expanded from timber production to include environment and social goals [287]. This is also around the time of the latest revisions of the CFSA in Ontario, in 1994 [53]. In the case of Ontario’s forest policy, the law and government resources thus provide a very stable supporting structure for more effective adaptive management.

Another example of a legal approach to adaptive management is the requirement for periodic reports to the legislature by a designated body (i.e. an agent), as seen in the EU through EU RED I from 2009, which requires that (emphasis added) “By 31 December 2012, the Commission *shall report* to the European Parliament and to the Council on: (a) the effectiveness of the system in place for the provision of information on sustainability criteria; and (b) whether it is feasible and appropriate to introduce mandatory requirements in relation to air, soil or water protection, taking into account the latest scientific evidence and the Community’s international obligations. The Commission shall, if appropriate, propose corrective action” [18]. Another example from the EU is the voluntary EU recommendations for national sustainability criteria for solid biomass used in the heat and power sector from 2010 for which “The Commission *will report* by 31 December 2011 on whether national schemes have sufficiently and appropriately addressed the sustainability related to the use of solid biomass from inside and outside the EU, whether these schemes have led to barriers to trade and barriers to the development of the bioenergy sector” [57]. A noticeable recent example of an adaptive feature in law is the German Renewable Energy Act (REA) from 2012, which requires monitoring, evaluation, and law revisions in a four-year cycle [92] (Table 10). Due to introduction of significant subsidies for biogas, and quickly evolving deployment, the adaptive feature was prioritised over keeping the law unchanged for a longer period of time.

Voluntary, flexible governance systems, with soft enforcement through reporting, monitoring and assessment, are ways to gain experiences while minimising the risk of introducing undesirable, unintended incentives and impacts from rules that are more inflexible to changes. The experiences from voluntary systems may form a basis for new decisions about which contents and policy style to apply if the voluntary system is changed to legally mandatory systems. For example, the voluntary Industry Agreement between private partners in Denmark on sustainable wood pellets and wood chips for energy, adopted in 2016, required evaluation of its effectiveness by 2018 [59] (Table 10). The evaluation was published in 2019 [61] and it is still to be decided if the voluntary partner agreement should be revised and replaced with legal requirements [311].

Private forest certification systems also include well-known adaptive features with requirements, for example, that forest management standards must be revised on a regular basis in participatory processes, normally in five-year cycles for PEFC-endorsed systems, even if three-year rotations apply for the SFI in North America (Table 10). Adaptive management approaches have also been proposed to manage sustainability of Bioenergy with Carbon Capture and Storage (BECCS), again, due to rapid developments and deployment in the area [312]. However, little is known about how different types of adaptive features in system designs link to granting of input, output and throughput legitimacy in different conditions, but further analysis of what adaptive features exist and how they function may be a platform for further exploring the topic.

#### Adaptive sustainability governance model

Informed by the ISEAL credibility criteria, and inspired by Rist et al. [304], Lattimore et al. [287], the case of AFM in Ontario [315], and Malekpour and Newig [3] we propose a model for adaptive sustainability governance with a monitoring and evaluation (M&E) system that incorporates adaptivity. The model reflects the finding that an increase in adaptive capacity positively affects the quality of the participatory process as well as the ability to react to contextual factors that are critical to policy impact [233]. It is the intent that the model can serve as a starting point for analyses and development of adaptive features in sustainability governance systems.

The proposed model comprises of a whole cycle for policy design, implementation and enforcement, and monitoring and evaluation of level of compliance, performance, efficiency, and satisfaction with the involvement, respectively (Fig. 8). The cycle should be repeated on a regular basis to identify needs for change in direction due to new situations or citizen or stakeholder concerns, the new information about the system’s impacts, and new technologies or knowledge that can improve the system.

A critical element in AFM is the M&E program and other platforms to facilitate adaptivity. The ISEAL Codes of Good Practice define an M&E as a system that tracks progress towards the intended goals, and evaluates the contribution of the governance system to long-term social, environmental or economic goals, but also how the system itself can develop and improve [210]. It is well-known from AFM that M&E systems can be costly and difficult to implement [287, 316], which is likely the reason that many sustainability governance systems do not have rigorous M&E systems beyond what is registered during controls or audits. In order to get started, monitoring of outputs and outcomes may be prioritised over monitoring of impact, which is more expensive and complex [138, 287]. Examples can be found with deliberate use of adaptive communication platforms to facilitate openness to stakeholder input and their mutual exchange of experiences and subsequent learning [91], as well as consensus building [173], but dedicated M&E systems to learn about stakeholder’s satisfaction with their input are rare.

## Conceptual governance research framework

Assuming that sustainability governance is a useful tool for sustainability transition of societies (Premise 4), with best possible sustainability governance systems established through development of PCI&V (Table 7), it is still necessary for governance systems to continuously review their overall approach. Such an exercise forces the governance system to make firm conclusions on lessons learned and address opportunities for changes that may have been captured through the monitoring and evaluation (M&E) systems or platforms for stakeholder communication (Fig. 8). Based on existing literature, we propose that the conceptual governance research framework presented in this section can be a useful tool in the review process.

### Approach

We took a starting point in the experiences and knowledge gained from the work conducted in the case studies published in this special issue, and continued to search more widely for relevant literature, based on an intuitive sense of relevance to research question of this paper:

How is the design of sustainability governance systems linked to people’s granting of legitimacy to the system and trust that the system leads to more sustainable outcomes for the regulated economic activity; how do these relationships depend on various institutional, economic, social and environmental factors?

It became clear that relevant literature could be found within a very wide range of scientific disciplines (Sect. "Approach", Additional file 1: Table S4), and the question emerged how the results from this great diversity of studies could logically be combined to provide a meaningful answer to the research question above. As other researchers faced with great unstructured diversity, we turned to classification and typology. As contemplated by the neuroscientist David Bor [317]:

“Some of our greatest insights can be gleaned from moving up another level and noticing that certain patterns relate to others, which on first blush may appear entirely unconnected “… “It becomes a positive feedback loop, making the detection of new connections even easier, and creates a domain ripe for understanding how things actually work, of reaching that supremely powerful realm of discerning the mechanism of things.”

Prominent examples of useful and successful pattern seeking are the nomenclature for plant taxonomy by Carl von Linné and Mendeleyev’s periodic table based on 56 elements that were known at that time, which could predict the existence of yet unknown elements from the table’s empty spaces. However, Bor [317] also warns about pitfalls of the human mind in saying that “We are so keen to search for patterns, and so satisfied when we've found them, that we do not typically perform sufficient checks on our apparent insights.” An example is the Ptolemaic geocentric astronomic system. Nevertheless, there are also modern examples that show the power of pattern seeking in policy science, for example, the typology suggested by McDermott et al. [79] for policy styles, which was inductively derived from exploring forest management policies. Nichiforel et al. [288] provide another example, where they uncover patterns of policies on property rights of private forests across European countries.

From the first identified articles, we thus continued the work by iteratively moving between reading, classifying, conceptualising, and looking for more relevant literature to complement where there seemed to be gaps, up to a point, where a typology had emerged to form a coherent whole and a framework to support research on sustainability governance systems could be developed, for integrated analysis, identification of research gaps and generation of knowledge to better understand what can improve sustainability governance designs. A table with detailed information was developed as a tool to keep track of literature and ideas (Additional file 1: Table S5).

In the remainder of this section section, we first describe the developed typology and explain how the elements are linked together (Sect. "Typology") in order to understand how the conceptual governance research framework can be applied in a research context (Sect. "The governance research framework and its application in research"). We then discuss how various types of studies situated within the framework can be used, in principle, as a basis for giving policy recommendations on how to increase the legitimacy of sustainability governance systems, for example, in the bioenergy sector (Sect. "The governance research framework and its application to provide policy recommendations").

### Typology

**Fig. 9:**
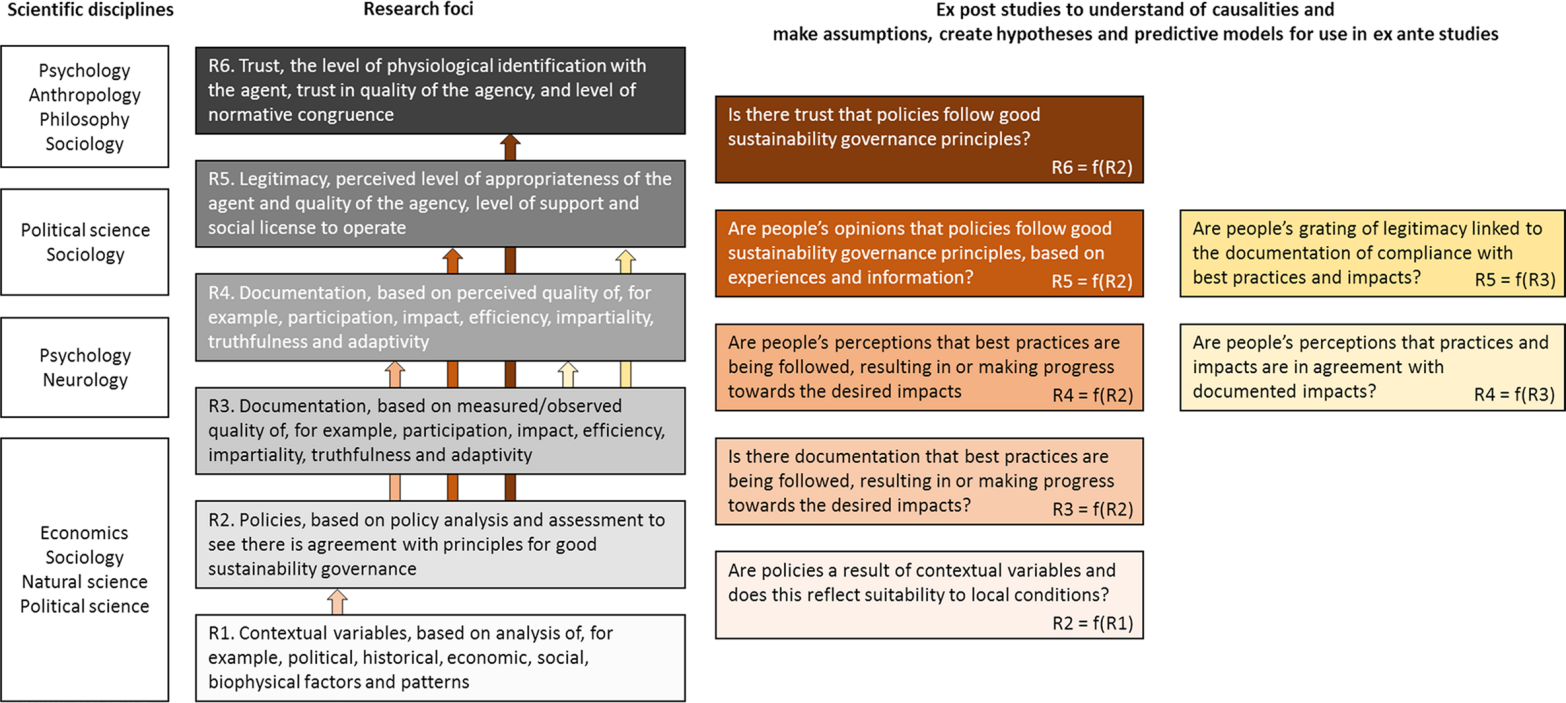
Conceptual governance research framework for integrated analysis, identification of research gaps, and generation of knowledge to better understand how legitimacy and trust are granted to sustainability governance systems or regimes. The framework is based on the typology presented in Table 11 including research foci (*R*1–*R*6) and the possible relationships between variables associated with them (indicated as a function, e.g. *R*3 = *f(R*2)). Brown boxes contain research questions to examine policies (*R*2) and their relationship with other research foci. Yellow boxes contain related questions that do not directly involve policy analysis. Ex post studies can help to understand causalities experienced in the past, which may help to make assumptions, create hypotheses and predictive models for use in ex ante studies to analyse the consequences of future policy scenarios in similar situations and conditions. White boxes indicate typical scientific disciplines involved for various research foci. The overall purpose of any analysis is to generate knowledge that can improve our understanding of what makes sustainability governance “good” (Table 7)

In this subsection, we first describe the three identified dimensions of our typology and general scientific approaches applied in policy analysis as a basis for linking the three dimensions together by use of common statistical principles. This helps us derive the conceptual governance research framework as a tool to ask questions that will aid in identifying the causes of sustainability governance crises, as well as designing new research to find solutions (Fig. 9).

#### The three dimensions

We identified three dimensions for the proposed typology. First, working our way through literature, *research focus* stood out as a clear difference among the relevant studies. The various research foci can be seen as corresponding to dependent or explanatory variables in statistical models. We identified six major research foci (R1–R6, Table 11), with a progression among them from context to policy, measured and perceived policy impact, and granted legitimacy and trust to governance systems and economic activities, even if all combinations are possible. The governance research framework presented in Fig. 9 intends to provide an integrated view of how each research focus is important to examining the overall research question of this paper about sustainability governance system or regime design. In line with the proposed principles for good sustainability governance (Table 7), many existing studies apply the input, output and throughput legitimacy concepts for their analysis. The choice to focus on any or more of the legitimacy aspects is embedded in the six research focus categories (not shown in Table 11 or Fig. 9).

**Table 11:** Typology for research relevant to studying the “goodness” of sustainability governance systems or regimes, and how to improve them to solve possible sustainability governance crises, using the bioenergy sector as an example

Dimension	Symbol	Value	Description with bioenergy as example
Research focus	R1	Context	Contextual variables, for example, political, historical, economic, social, biophysical factors and patterns
	R2	Policy	Policy variables, e.g., related to bioenergy, forest, agriculture, nature conservation, or waste policies or economic, environmental, social policies more generally
	R3	Measurable impact	Measurable impacts (parameters and indicators) of bioenergy, forestry, agriculture activities, or activities in another of the above-mentioned sectors
	R4	Perceived impact	Perceived impacts (parameters and indicators) of bioenergy, forestry, agriculture activities, or activities in another of the above-mentioned sectors
	R5	Legitimacy	Granted and achieved legitimacy of bioenergy, forestry, agriculture activities, policies or sustainability governance systems, or of those sectors mentioned above
	R6	Trust	Granted and achieved trust in sustainability of bioenergy, forestry, agriculture activities and policies or in the trust that the sustainability governance system achieves sustainability goals that it was designed to achieve
Comparative approach	A1	Temporal	Examining changes over time for one or more geographies or policies
	A2	Geographies	Examining differences in policies between different geographies, typically jurisdictions
	A3	Levels	Examining overlaps and complementarity of different levels of governance in a multilevel governance regime (Fig. 2)
Research question	Q1	If	Corresponding to a statistical test to show “if” a change or a difference is statistically significant
	Q2	How	Concerns the exact patterns in data, i.e. the nature of a change or a difference, for example, how did a policy change or how do policies differ, cf. the classifications systems presented in Sect. "Assessing if sustainability governance systems are good"
	Q3	Why	Concerns “why” certain changes or differences occur and if there is correlation and maybe causality

Second, there were differences in the chosen methodological approaches, which could be experimental, statistical, or comparative, or a singular case. Most approaches, however, included one of three different *comparative approaches*, and compared polices either at different points in time (A1), among different geographies (A2) or among different levels in a multi-governance regime (A3) (Table 11). Studies focusing on the changes over time were usually limited to one or very few geographical settings, while studies across geographies or multiple levels of governance were often limited to one or very few points in time. This reduces complexity and makes scientific analysis more practical and economically feasible.

Third, studies could further be classified according to the types of *research questions* they ask, i.e. asking “if” there is a change or difference (Q1), “how” something changes or differs (Q2), or “why” it change or differs (Q3) (Table 11). The question asked will lead to results that are descriptive or explanatory in nature, i.e. descriptive if the study classifies observations (“how does something differ”), and explanatory, if the study seek correlations among research foci variables (“why” does something differ), respectively. Koven [312] provides an example of a study that address all three types of research questions, i.e. if forest polices in Ontario changed during the period 1998–2014, how they changed, and why.

#### Methodological approaches in a policy study perspective

As a basis for explaining the linkages between the three dimensions of the typology, we describe the strengths and weaknesses of various methodological research approaches applied in policy studies, including experimental, statistical, comparative and case study approaches, based especially on Collier [318].

A commonly experienced barrier to make experiments is the need to establish a proper control [318] or counterfactual [245]. This requires the existence of regulated and unregulated units or operations, that are comparable except from the studied regulatory activities. These units should also be available to the experimental work that may involve relatively long-term monitoring of developments over time to detect possible changes. However, ever changing economic, social and environmental factors are hard to control, which makes it hard to find units that are truly comparable. The speed of evolution is a special challenge for certification systems; considerable time and resources are needed to set up the experimental work, and by the time the research results are available, the time may have passed to make policy recommendations that are relevant [79].

For statistical approaches, it is often a challenge to identify or collect sufficiently large and reliable data sets [318]. For this reason, comparative and case studies have become especially important in investigations of policy design and impact, even if these approaches also have their limitations and challenges. A case study is often the most feasible approach but results are hard to generalize, and it is hard to unravel causality and create predictive models. The number of possible explanations will often exceed the number of available case studies, corresponding to a situation with many possible predictor variables and only one or few observations in statistics [318].

Regardless of the comparative approach (A1-A3), it may be difficult to obtain enough observations to allow for statistical analysis [319]. However, the use of typologies, classification and characterisation systems for a structured comparison of policies can still provide a critical basis for theoretical and empirical analyses of the strengths and weaknesses of different policies [165]. Such information can also be a useful basis for concept formation, inductive creation of hypotheses and theories, and perhaps future statistical analysis and predictive models [318].

Another challenge in policy analysis may arise from the diversity of data to be compared among points in time or between geographies or levels in a multilevel governance regime. In an analysis comparing SFM policy variables, McDermott et al. [79], for example, experience that prescriptive legal requirements were easier to compare than less prescriptive requirements (Table 11), thus adding uncertainty from interpretation of the latter. The authors emphasize the usefulness of large comparative studies for identifying coarse-grained policy patterns, while more contextual information would be needed to draw firm conclusions regarding on-the-ground policy impacts. In-depth case studies may be helpful to compensate such weaknesses of larger comparative studies.

#### Links between the three dimensions of the typology

The three dimensions of the typology are conceptually linked in the same way as the elements of a statistical model created to test a hypothesis. In line with McDermott et al. [79], we use the term “policy setting” to refer to the specific research area targeted by the policy analysis, for example, biodiversity conservation or legality of harvested wood. Each policy setting consists of a set of policy variables or on-the-ground requirements, e.g. requirements for riparian zone management and specific rules to confirm legality. Hence, political science meets natural, social or other sciences through the policy variables.

If *R*2_*i*_ is a policy variable (Table 11), and *i* = 1, 2, 3….*n* expresses the number of policy settings being compared, then a model is needed to detect if *R*2_*i*_ change over time (A1, Table 11), differ among geographies (A2, Table 11), or differ among levels in a multilevel sustainability governance regime (A3, Table 11).

The research question “if” (Q1) corresponds to a statistical test to show “if” a significant difference in values of a policy variable occurs among policy settings at different points in time, among geographies, etc., even if the nature of policy variables may not allow testing in a strict statistical sense. After detecting an overall difference, it may be further evaluated how a policy variable, *R*2_*i*_, differs, for example, with regard to policy contents, policy style, use of risk-based elements in verification, nature of applied adaptive features, etc., cf. Sect. “Assessing if sustainability governance systems are good”, i.e. what are the policy patterns. An example is again the study by McDermott et al. [79] that compares forest management policy variables, *R*2_*i*_, among more than 30 jurisdictions globally (*i *> 30). This analysis is mainly descriptive, focussing on the classification of the different policy styles to create structured information, from which implications for effectiveness were discussed and elucidated.

In policy studies, it is often complicated to proceed to a data-based analysis of correlations that can potentially be used for predictive purposes, but correlations can, in principle, be examined with the following model (Fig. 9):

$$Y_{i} = f\left( {R2_{i} } \right) + e_{i}$$

where *Y*_*i*_ is research foci *R*3, *R*4, *R*5 or *R*6. Studies may thus examine the relationship between policies and e.g. their measurable (*R*3) or perceived (*R*4) impacts before and after implementation of a policy, or based on differences among geographies. Examples could be studies of the development of the biogas sector, where *Y*_*i*_ = *R*3_*i*_ is the number and size of biogas plants in Denmark [91] or Germany [92], as new policies (*R*2_*i*_) are introduced, *i* = various points in time (A1, Table 11). Another example could be a study of the level of biodiversity conservation (*R*3_*i*_) in FSC certified forest, *i* = Sweden and Russia (A2, Table 11) [266], or a study of the level of water BMP implementation, *R*3_*i*_, for *i* = SFI certified and uncertified forest in Georgia, the US (A2, Table 11) [140].

There are also examples of studies that examine why different policies occur in different settings, and how such differences may be linked to differences in contextual characteristics (*R*1, Table 11).

$$R2_{i} = f\left( {R1_{i} } \right) + e_{i}$$

As an example, Koven [313] addresses how changes in forest policies in Ontario (*R*2_*i*_), *i* = the period 1988–2014 (A1, Table 11), occurred as a consequence of *R*1_*i*_ = emerging new networks of actors that increasingly gained power over policies, explained by “successful political advocacy”, “failed tactics causing estrangement from the government” or “ongoing budget cuts” and “economic crisis”, depending if the network consisted of environmental NGOs or actors from the traditional forest management and timber harvesting sector. Another example is a study by Ring et al. [320] who compare forest management requirements (*R*2_*i*_) for riparian zones in *i* = selected Nordic and Baltic countries (A2, Table 11), and suggest that differences could be related to for example, *R*1_*i*_ = historical differences in political systems among Nordic and Baltic countries. Another study also examines how perceived changes in police behavioural performance (*R*4_*ijk*_) are related to policy and measurable behavioural changes (*R*3_*i*_), *R*4_*ijk*_ = *f* (*R*3_*ij*_) + *e*_*ijk*_, *i* = before and after the policy change (A1), *j* = the US and Europe (A2), *k* = replication, e.g. asked individuals [301].

### The governance research framework and its application in research

The typology and described linkages among its dimensions can be applied as a tool to systematically go through possible causes of sustainability governance crises (Fig. 9). Combinations of research foci may also be used to create research questions to address knowledge gaps and conduct new research. Such research questions may involve direct examination of policies as explanatory variables (brown boxes, Fig. 9), or examination of which contextual factors have potentially lead to the differences in policy contents and styles among different settings, even if the intent of the policy and the biophysical conditions were comparable. It may also be relevant to study other related questions even if these do not directly address the policy setting (yellow boxes, Fig. 9). Several other relevant research questions than those shown in Fig. 9 are theoretically possible and may be relevant.

Ex post studies can help to understand causalities experienced in the past that may help to make assumptions, create hypotheses and predictive models for use in ex ante studies that analyse consequences of future policy scenarios in similar situations and conditions. It is always embedded with uncertainty when historical findings about relationships are used to make predictions for the future, but some mechanisms will likely be more robust over time than others. Otherwise, this is where adaptive governance frameworks have a complementary role to play in policy development (Fig. 8).

### The governance research framework and its application to provide policy recommendations

The conceptual governance research framework (Fig. 9) is indicative of how different types of scientific studies can, individually and all together, form the basis for practical policy recommendations on how to improve sustainability governance system design to achieve higher levels of legitimacy and trust, possibly with each system being an element of a larger sustainability governance regime relevant to a particular issue.

As mentioned above, verified or hypothesised relationships between policies and their impacts may form the basis for ex-ante policy analysis to examine the possible outcomes of alternative theoretical policies [321] and sustainability governance system designs. The main aim of ex-ante policy analysis is to predict how the introduction of alternative new policies may change people’s behaviours in a way that leads to a desired social, economic or environmental change [322]. Such analyses have traditionally been conducted in the context of public administration and public policy making but could possibly also be developed to address private governance systems, or broader multilayered governance regimes.

Comparison of different policy settings among different geographies may push evolution of policies and possibly induce convergence over time, because of an improved understanding of general norms that exist across jurisdictions or governance regimes [323, 324]. Policies that are perceived as successful in one place may be adopted in another place with similar challenges and conditions or point to conditions that need to be changed before policies can be successfully adopted, implemented or enforced. Studies of contextual drivers of policy development over time may also provide a valuable knowledge source that may lay the ground-work for transfer of successful approaches to other settings or policy areas. As experiences with the WGI indicators have shown, however, there are also reasons to be cautious before assuming that governance experiences from one setting can automatically be transferred with success to a different one.

Comparison of policy settings at different levels in a multi-governance regime will obviously be useful to indicate where resources might be saved due to overlap among governance systems or identify where gaps exist that need to be filled. However, overlaps may be acceptable to maintain overall legitimacy to the extent that each governance system has been endorsed by its citizens or stakeholders and accurately represents their concerns, needs and interests.

Comparison of perceived and measured policy impacts may inform the agents about possible needs for strategies to close gaps between measurable and perceived impact. Analyses of the levels of legitimacy or trust related to certain policies will also be helpful to inform policy makers about problems, or the nature of these, as a basis for developing new policies or strategies for engaging with citizens or stakeholders to learn about their concerns and interests, and possibly finding higher levels of commonality in views.

Economists are often responsible for policy analyses aiming at practical policy recommendation to governments. A common assumption in economics is that the regulated actors’ behaviours are rational with this allowing identification of an optimal theory-based “first-best” policy, based on effectiveness and cost-efficiency as good governance evaluation criteria [4]. However, actors will often not rationally balance the benefits and the costs of abiding a policy. For this reason, theories of “second-best” options emerged in the early 2000s as an alternative to “first-best” approaches. In the context of bioenergy policies, Purkus [321] identifies six types of limitations to “first-best” approaches that may advocate the choice of “second-best” solutions. Limitations include, market failures due to externalities; limited consideration of uncertainty; neglect of transaction costs; neglect of political feasibility constraints in situations of great complexity; and neglect of institutional context. In line with this, van den Bergh et al. [4] argue that it is also necessary to introduce “third-best” policy approaches which take account of the interaction between policies and technology, institutions, social and economic subsystems of specific sectors, for example, energy, water, food, and housing. When policies aim at sustainability transition, they advocate “third-best” approaches. This is also in agreement with McDermott et al. [79] who warn about drawing conclusions about effectiveness of policies without consideration of the context to which these policies are applied.

While scientific knowledge is critical to make informed and sound political decisions, there are also limitations to what science can solve, as seen from both practical experiences and theoretical considerations. This highlights, again, that other means are necessary in situations with imperfect knowledge, lack of resources to generate the necessary knowledge, or when transfer of knowledge from one situation to another is not meaningful. In the search for second-best or third-best solutions, adaptive governance is likely the best solution we have at hand (Fig. 8).

## Outlook

In the context of developing and operating sustainability governance systems and regimes, several studies emphasize the need to consider the broader context, which include social norms, technology, institutions and markets [4, 325]. Moog et al. [326] go further to suggest that the lack of supportive global governance structures for environmental protection has limited the effectiveness of multi-stakeholder governance initiatives, such as the FSC, and prevented them from reaching their full potential, in spite of their success as a platform for deliberation of social and ethical responsibilities of corporations.

Biermann [327] and Dodds et al. [328], among others, expand on this argument further based on an identified gap in global level governance that calls for better balance between economic development and environmental conservation and mitigation of inherent impact of economic activity. Such criticism generally focuses on the voluntary nature of international agreements, with no mechanism for enforcement and limited incentive to join. In many countries, it is easier for a government to pull out of an environmental agreement than to sign onto one. For example, during the year 2013, the Canadian government unilaterally withdrew from a number of international agreements aimed at environmental conservation, including the International Tropical Timber Organization (ITTO) and the UN Convention to Combat Desertification [329]. While the withdrawal was unilaterally decided by the government in power, the ratification of a new environmental agreement is a long and complicated process requiring the involvement of relevant departments and agencies to develop memoranda and guide the international agreement, whether binding or non-binding, through a multi-year tabling process to be made into a policy. While participation in non-state market-driven governance, such as certification, is commonly understood as a mitigation measure, Moog et al. [326] also find that such participation can reinforce governmental withdrawal. This is most unfortunate, if the effectiveness of such private systems depends on the existence of rigorous global governance structures.

The need to rethink the design and capacity of global institutions to better reflect modern-day challenges has long been expressed. Dodds et al. [328] and Medhora and Owen [330] suggest tax reporting as a tool, as multinational corporations generally report profits in countries with the lowest corporate-tax rates, but the pattern is again that governments pull out; the latest vote by EU member states rejected legislation that would have required corporations to report earnings and pay taxes within each EU country. Medhora and Owen [330] conclude that these distorted principles of equity and efficiency in tax regimes are due to the lack of international governance and coordination.

This has led to calls by scholars, such as Dodds et al.[328], to develop autonomous global multilateral institution that could enforce multilateral environmental agreements between nations, in the same way as the World Trade Organization (WTO) enforces international trade agreements between nations. The authors note that the WTO has overcome the challenges facing global governance and accrued immense power as an international negotiating, coordinating and judicial authority. For example, the WTO hosts a dispute settlement system, where member countries may bring complaints that a trade agreement has been violated by a nation, and where an enforceable penalty may be imposed. Such penalties may reach hundreds of millions of dollars, which is a significant incentive that incentivises countries to comply. Trachtman [331] identifies various areas within WTO legislation and case law that offer the possibility that the WTO may begin to make rulings related to the violation of international environmental law. He points to the *U.S.-Gasoline* decision,^6^ in which the WTO Appellate Body wrote that the pre-eminent WTO law, the General Agreement on Tariffs and Trade (GATT), “is not to be read in clinical isolation from public international law." In the context of trade agreements, which are agreed between nations upon common, environmental provisions or international environmental agreements, should be incorporated. As an example, 21 member states of the Asian Pacific Economic Cooperation Initiative (APEC) and the Environmental Goods Agreement (EGA) agreed to reduce tariffs across 54 product categories that are meant to be environmentally friendly, including the criteria that they benefit the climate (e.g. solar water heaters) [332, 333]. Notably, the US has imposed conditions on the WTO dispute-settlement system, which some authors suggest has resulted in it turning into a stagnant and ineffective system [331, 334]. In a situation where countries move away from intergovernmental institutions, Trachtman [331] and Mayroidis and Neven [332] argue, that, at minimum, trade agreements and the WTO dispute settlement system should be protected from deterioration as tools to implement and enforce global sustainability governance regimes.

There are signs that as society moves towards more realism in how we must understand international relations, nations may increasingly base their foreign policies on self-preservation rather than collaborative liberalism, resulting in the erosion of free markets and other global processes. At the time this paper was written, the COVID-19 pandemic had spread throughout the world, and it has been questioned if this catastrophe has the power to disrupt the trend of realism in favour of more collaborative and globally sustainable directions. According to McKibben [335], the acuteness of the pandemic has revealed that societies are vulnerable and not built to be sturdy when faced with a global crisis. As governments try to deal with COVID-19, an isolated concern, it becomes clear that the consequences of climate change, an all-encompassing concern, will become even more catastrophic with increasing barriers to implement preventative and mitigating measures, for example, in relation to reducing GHG emissions, combatting flooding, or fighting forest fires. It has also become more difficult to deal with criminal acts, such as deforestation of the Amazon rainforest [336].

## Conclusion

Human choices about our activities have severe harmful impacts on the life of other human beings and organisms on this planet, with various forms of sustainability governance in place that incentivise or restrict behaviours in a way that puts us on a trajectory towards more sustainable societies. However, there is still immense concern over undesired sustainability impacts of economic and subsistence activities and criticism that existing sustainability governance is failing to achieve its aims and overall sustainability. We could call this a sustainability governance crisis. With this paper, we set out to learn from existing literature on how the design of sustainability governance systems is linked to their effectiveness, people’s granting of legitimacy to the system and their granting of trust in the governed activities being a solution that promotes sustainability. We approached our learning with an iterative search for structure in a broad, disparate, but still relevant body of literature, and derived what we chose to call a governance research framework. The framework can be used to systematically search for causes of the sustainability governance crisis and to develop solutions to it, for example through the syntheses of information and commissioning of new studies on the extent to which sustainability policies and governance system designs create real or perceived impact, or facilitate the granting of legitimacy and trust. Ex post studies can thus help to understand the mechanisms behind such relationships for existing policies, which may help to make valid assumptions, create hypotheses or predictive models for use in ex ante studies that analyse potential consequences of future policy scenarios in similar situations and conditions. However, as it is also evident from the literature, that there are significant limitations to predict-and-act governance solutions with the best alternative at hand being the embedding of good sustainability governance systems in adaptive governance setting. Based on literature, again, we conveyed, through suggestions for principles and criteria, a summary of what is known about the factors that make sustainability governance “good” and how such principles and criteria relate to an adaptive governance approach. In a globalised world with very high levels of connectivity, we suggest with others, that there is a need to create new empowered global level governance institutions, which focus more on sustainability and robustness of our societies than the present ones. Until international cooperation is able to bring about such reform, sustainability governance allowing for gradual improvements at smaller scales is likely to be the most important tool for transitioning towards more sustainable societies. It is at this level that this paper intends to make a contribution, mainly, by providing a conceptual tool for analysing and finding solutions to situations with a complex legitimacy and trust crisis, where governance systems are used to obtain trust in the sustainability of specific policies and practices, for example in the bioenergy and bioeconomy sectors.

## Supplementary Information

**Additional file 1: Table S1.** Benchmark of good governance principles; **Table S2.** Classification of risk-based elements; **Table S3.** Classification of transparency level; **Table S4.** List of literature upon which Table 11 and Fig. 9 is based, **Table S5**. Working table upon which Fig. 9 is based, including reference to literature from Table S4.

## Data Availability

All data generated or analyzed during this study are included in this article, or clearly referenced.

## Abbreviations

AFM: Adaptive Forest Management
APEC: Asian Pacific Economic Cooperation Initiative
AWG: Anthropocene Working Group
BAU: Business as usual
BECCS: Bioenergy with carbon capture and storage
BMP: Best management practices
BSCR: Standards of Biomass Supply Chain Risk
CAR: Corrective action requests
CFSA: Ontario Crown Forest Sustainability Act
CSA: Canadian Standards Association
CSR: Corporate Social Responsibility
EGA: Environmental Goods Agreement
ELoGE: Council of Europe published the European Label of Governance Excellence
ETS: EU Emission Trading System
EU: European Union
EU TR: European Union Timber Regulation
FoE: Friends of the Earth
FSC: Forest Stewardship Council
GATT: General Agreement on Tariffs and Trade
GDP: Gross Domestic Product
GGL: Green Gold Label
GHG: Greenhouse gas
GRI: Global reporting indicator
IEA: International Energy Agency
iLUC: Indirect land use changes
IMF: International Monetary Fund
ITTO: International Tropical Timber Organization
ISEAL: International Social and Environmental Accreditation and Labelling Alliance
LULUCF: Land use, land use change and forestry
M&E: Monitoring and evaluation
MDGs: UN Millennium development goals
NGO: Non-Governmental Organization
PCI&V: Principles, criteria, indicators & verifiers
PEFC: Programme for the Endorsement of Forest Certification
R&D: Research & development
REA: German Renewable Energy Act
RED I: Renewable Energy Directive (2009)
RED II: Renewable Energy Directive (2018)
RenovaBio: Brazil’s Biofuels National Policy
RFS: Renewable Fuels Standard
RSPO: Roundtable on Sustainable Palm Oil
SBP: Sustainable Biomass Program
SDGs: Sustainability Development Goals
SDO: Standard Development Organization
SFI: Sustainable Forestry Initiative
SFI-FS: Sustainable Forestry Initiative Fiber Sourcing
SFM: Sustainable Forest Management
SLO: Social License to Operate
TCFD: Task Force on Climate Disclosure
UK: United Kingdom
UN: United Nations
US: United States of America
WGI: World Bank Worldwide Governance Indicators
WTO: World Trade Organization
WWF: Worldwide Fund for Nature

## Footnotes

1 MacPherson v. Buick Motor Co. [1916] 217 NY 382 (United States of America, New York Court of Appeals).

2 Donoghue v. Stevenson, [1932] UKHL 10 (United Kingdom House of Lords).

3 Urgenda Foundation v. The State of The Netherlands (Ministry of Infrastructure and the Environment), [2015] Case C/09/456,689/HA_ZA 13–1396 (District Court of The Hague, Chamber for Commercial Affairs).

4 Leghari v. Federation of Pakistan, (2015) W.P. No. 25501/201 (Pakistan, Lahore High Court).

5 Re Greenpeace Southeast Asia and Others, [2019] Case No. CHR-NI-2016–0001 (Philippines Commission on Human Rights).

6 US-Gasoline, 2006 WT/DS2/AB/R (World Trade Organization Appellate Body).
