# Supplementary material for: Conceptual framework for increasing legitimacy and trust of sustainability governance
Source: Energy Sustain Soc. 2021 Mar 18;11(1):5. doi: 10.1186/s13705-021-00280-x (PMC7972028; doi:10.1186/s13705-021-00280-x)
Supplement: Supplementary file 1 — Additional file 1: Table S1. Benchmark of good governance principles; Table S2. Classification of risk-based elements; Table S3. Classification of transparency level; Table S4. List of literature upon which Table 11 and Fig. 9 is based, Table S5. Working table upon which Fig. 9 is based, including reference to literature from Table S4. [file 13705_2021_280_MOESM1_ESM.docx]

**Conceptual framework for increasing legitimacy and trust of sustainability governance**

Inge Stupak^a,^*, Maha Mansoor^b^, C. Tattersall Smith^b^

^a^Department of Geosciences and Natural Resource Management, University of Copenhagen, Rolighedsvej 23, DK-1958 Frederiksberg C, Denmark, ism@ign.ku.dk

^b^University of Toronto, Canada, mahamansoor@outlook.com, tat.smith@utoronto.ca

*corresponding author, ism@ign.ku.dk

# Supplementary

Submitted to Energy Sustainability and Society

19. January 2021

**Table of Contents of the main text of the article**

**INTRODUCTION**

**THE EXAMPLE OF BIOENERGY**

BIOENERGY POLICY AND SECTOR DEVELOPMENT

BIOECONOMY POLICIES

THE CONFLICTING VIEWS OVER THE SUSTAINABILITY OF BIOENERGY

GOVERNANCE AS A TOOL TO RECONCILE CONFLICTING VIEWS OVER SUSTAINABILITY OF BIOENERGY

**APPROACH**

**UNDERLYING PREMISES, CONCEPTS, AND TERMINOLOGY**

PREMISES

Human choices about our activities significantly impact life on Earth and there is a duty to care to transition towards more sustainable societies

Societal trust is needed to transition towards more sustainable societies and governance is a tool

Cooperation is needed to shape good sustainability governance systems

KEY CONCEPTS AND TERMINOLOGY

Sustainability

Sustainability transition

Sustainability governance

Trust

Legitimacy

PRINCIPAL-AGENT RELATIONSHIPS

Types of agents and agent systems

The principal

Principals’ motives to grant legitimacy and trust

Agents’ strategies to achieve legitimacy and trust

**ASSESSING IF SUSTAINABILITY GOVERNANCE SYSTEMS ARE GOOD**

THE HISTORY OF THE GOOD GOVERNANCE CONCEPT AND EXISTING ASSESSMENT FRAMEWORKS

DEFINING GOOD SUSTAINABILITY GOVERNANCE AND PROPOSING AN ASSESSMENT FRAMEWORK

INPUT LEGITIMACY

Defining input legitimacy and level of ambitions

Criteria for quality of the stakeholder participation

OUTPUT LEGITIMACY

Defining output legitimacy and level of ambition

Criteria for the governance system effectiveness

Criteria for the governance system efficiency

Policy content and style as a basis for assessing rule effectiveness

Types of enforcement strategies as a basis for assessing degree of enforcement

THROUGHPUT LEGITIMACY

Defining throughput legitimacy

Criteria for governance system fairness

Criteria for governance system truthfulness

ADAPTIVE SUSTAINABILITY GOVERNANCE SYSTEMS

The need for adaptive governance

Adaptive elements in existing governance systems

Adaptive sustainability governance model

**CONCEPTUAL GOVERNANCE RESEARCH FRAMEWORK**

APPROACH

TYPOLOGY

The three dimensions

Methodological approaches in a policy study perspective

Links between the three dimensions of the typology

THE GOVERNANCE RESEARCH FRAMEWORK AND ITS APPLICATION IN RESEARCH

THE GOVERNANCE RESEARCH FRAMEWORK AND ITS APPLICATION TO PROVIDE POLICY RECOMMENDATIONS

**OUTLOOK**

**CONCLUSION**

**Table S1** Benchmark of principles and criteria for “good governance” across different systems described in the literature [1-9], grouped by the authors of this paper under four different “principles” that largely correspond to the tripartite legitimacy concept, with an additional category for substantive content. Numbers in round brackets refer to the number of the principle in the original publication.

| Principle | Governmental governance | | | Private governance | Scientific studies (by example) | | | | |
| --- | --- | --- | --- | --- | --- | --- | --- | --- | --- |
|  | Global | Regional | | Global | Government | Climate | Forestry | Maritime | EU multilevel |
|  | World Bank WGI [1] | UNESCAP.[2] | Council of Europe [3] | ISEAL [4] | Rothstein [5] | Davidovic and Harring [6] | Göhler et al. [7] | Bennett and Sattersfield [8] | Schmidt and Wood [9] |
| Justice, impartiality, accountability, transparency, truthfulness  (throughput legitimacy) |  |  |  | Impartiality (6) | Impartiality |  |  |  |  |
|  | Rule of Law (5) | Rule of law (8) | Rule of Law (5) |  |  | Law and order |  |  |  |
|  |  | Equitable (7) |  |  |  |  |  | Equitable** |  |
|  | Control of Corruption (6) | Corruption minimized |  |  |  | Corruption |  |  |  |
|  |  | Transparent (4) | Openness and Transparency (4) | Transparency (7) |  |  | Transparency |  | Transparency |
|  | Regulatory Quality (4) |  |  |  |  | Bureaucracy quality |  |  |  |
|  | Accountability (1) | Accountable (3) | Accountability (12) | Rigour (4) |  |  | Accountability |  | Accountability |
|  |  |  | Ethical Conduct (6) | Truthfulness (9) |  |  |  |  |  |
|  | Political Stability and Absence of Violence (2) |  |  |  |  |  |  |  |  |
|  |  |  | Innovation and Openness to Change (8) |  |  |  |  |  | Openness |
|  |  | Inclusive (7) |  |  |  |  | Inclusiveness |  | Inclusiveness |
| Representation, participation, voice, responsiveness (input legitimacy) |  |  | Representation (1) |  |  |  |  |  |  |
|  | Voice (1) | Participatory* (1) | Participation (1) | Engagement (5) |  |  |  |  |  |
|  |  | Responsive (5) | Responsiveness (2) |  |  |  |  | Responsive***** |  |
|  |  |  |  |  |  |  |  | Robust*** |  |
|  |  | Consensus oriented (2) |  |  |  |  |  |  |  |
| Outcome, impact, effectiveness and efficiency  (output legitimacy) |  |  |  |  |  |  |  |  |  |
|  | Government Effectiveness (3) | Effective (6) | Effectiveness (3) | Improvement (2) |  |  |  | Effective**** |  |
|  |  |  | Competence and Capacity (7) |  |  |  |  |  |  |
|  |  |  |  | Accessibility (8) |  |  |  |  |  |
|  |  |  |  | Relevance (3) |  |  |  |  |  |
|  |  | Efficient (6) | Efficiency (3) | Efficiency (10) |  |  |  |  |  |
| Values/substantive contents |  |  | Sustainability and Long-Term Orientation (9) | Sustainability (1) |  |  |  |  |  |
|  |  |  | Sound Financial Management (10) |  |  |  |  |  |  |
|  |  |  | Human Rights, Cultural Diversity and Social Cohesion (11) |  |  |  |  |  |  |
| *Minority views are taken into account, and the voices of the most vulnerable in society are heard in decision-making  ** Recognition, Participation, Fair, Just  *** Legitimate, Connected, Nested, Polycentric  **** Direct, Coordination, Capacity, Informed, Accountable, Efficient  ***** Learning, Anticipatory, Adaptive, Innovative, Flexible | | | | | | | | |  |

| **Table S2** Formalised and informal risk-based elements in different types of certification when considering the whole supply chain, based on Stupak and Smith [10]. Risk may be evaluated, for example in terms of quantity, severity, and frequency of the impact. | | | | |
| --- | --- | --- | --- | --- |
|  | Forest management unit (FMU) level certification | Group certification | Risk-based certification, with supply base evaluation (SBE) | Risk-based certification, with regional risk assessment (RRA) |
| Coverage in a region, jurisdiction, or country | Certified organizations | Certified organizations | All certified and uncertified FMUs supplying the biomass producer (BP), if specified risks are mitigated | All certified and uncertified organizations in a region or country, if specified risks are mitigated |
| No coverage of the region, jurisdiction, or country | Non-certified organizations | Non-certified organizations | Uncertified organizations outside the certified biomass producer’s supply base | Uncertified organizations that do not mitigate specified risks, e.g. if they do not supply certified biomass producers |
| Area subject to the possible risk assessment | Management unit area | Aggregated area of the group | Supply base area of the certified biomass producer | All potential supply areas in a region or a country |
| Unit responsible for risk assessment | Auditor | Auditor | Biomass producers, usually commissioning the assessment to consultancy | Collaborative group of biomass producers and possibly users, usually commissioning the assessment to consultancy |
| Assessor of the risk | Individual auditor | Individual auditor | The organization that is responsible and candidate to get certified, with third party auditing | The body that coordinates and is responsible for the RRA, with third party auditing |
| Level of formalization of the risk-based element | Low  Instruction or guidance may exist on how to decide on priorities for the limited auditing time and resources | Low  Instruction or guidance may exist on priorities for which organizations to select for auditing. | High  Formalized written risk assessment procedures, with reporting of the outcome that is subject to public consultation | High  Formalised written risk assessment procedures with reporting of the outcome that is subject to public consultation. |
| Nature of the risk-based element | Selection of issues to be audited | Selection of the group members to be audited + selection of the issues to be audited | Risk assessment to appoint indicators with specified risk | Risk assessment to appoint indicators with specified risk |
| High auditing priority | Sustainability criteria that entail the largest risk in a certified property | Organizations that entail the largest risk to sustainability goals of the standard among the certified organizations within the group | Sustainability indicators with specified risks, for implementation of mitigation measures | Sustainability indicators with specified risks, for implementation of mitigation measures |
| Low auditing priority | Other sustainability criteria with low risk in the certified property | Other forest owners in the group | Sustainability indicators assessed with low risk, for which no mitigation measures are needed | Sustainability indicators assessed with low risk, for which no mitigation measures are needed |
|  | | | | |

| **Table S3** Data, information, and knowledge collected and processed at difference scales, illustrating the differences in transparency depending if data, information. and/or knowledge are publicly available. | | | |
| --- | --- | --- | --- |
| -------------------------------- Increased information and knowledge transparency ------------------------------> | | | |
| <-------------------------------------------- Increased data transparency ----------------------------------------------- | | | |
| Scope of verification | Data collection  (data) | Data presentation (information) | Data interpretation (knowledge) |
| Landscape or jurisdictional level | Landscape level measurements and registrations at the international, national, sub-national or local level, e.g. databases with on-the-ground data, aerial photos, remote sensing data etc. | Geographically explicit data presentation, e.g. maps, graphs, tables, with links back to the data on which it is based | Scientific reporting of landscape level information with appropriate context-specific interpretation and discussion, also about accuracy, uncertainty and risk of bias, and links back to the information and data on which it is based |
| Company sourcing area level | Company level measurements and registrations for the sourcing area, e.g. on-site data, e.g. collected by drones, or possibly aerial photos, remote sensing data etc. | Geographically explicit data presentations, e.g. maps, graphs, tables, with links back to the data on which it is based | Companies’ sustainability reporting, with appropriate context-specific interpretation and discussion and links back to the information and data on which it is based |
| Management unit level | Company audit documentation files | Audit report for the company, with links back to the document files on which it is based | Full interpretation and qualified discussion of the audit report in terms of sustainability goal achievement and progress towards sustainability goals |

| **Table S4** Selected references identified as useful for developing the typology in Table 11, and the conceptual governance research framework in Fig. 9. | |
| --- | --- |
| No | Reference |
| 1 | Abbott, K.W. and Snidal, D. 2009. The Governance Triangle: Regulatory Standards Institutions and the Shadow of the State. In The Politics of Global Regulation. Princeton University Press [11] |
| 2 | Al-Seadi, T., Stupak, I., and Smith, C.T. 2018. Governance of environmental sustainability of manure-based centralised biogas production in Denmark. IEA Bioenergy Task 37, Edited by: Jerry D. Murphy (MaREI Centre, University College Cork, Ireland). Rep. Report 2018:7 [12] |
| 3 | Bennett, N.J., Di Franco, A., Caló, A., Nethery, E., Niccolini, F., Milazzo, M., and Guidetti, P. 2019. Local support for conservation is associated with perceptions of good governance, social impacts, and ecological effectiveness. Conservation Letters 0(0): e12640. doi: doi: 10.1111/conl.12640 [13] |
| 4 | Buliga, B. and Nichiforel, L. 2019. Voluntary forest certification vs. stringent legal frameworks: Romania as a case study. Journal of Cleaner Production 207: 329-342 [14] |
| 5 | Cheung, Q., Smith Jr, C.T., and Stupak, I. 2019. Governance of sustainable forest management and bioenergy feedstock harvesting in Ontario, Canada. Rep. IEA Bioenergy: Task 43: TR2019:04 [15] |
| 6 | Dale, V.H., and Kline, K.L. 2017. Interactive posters: A valuable means of enhancing communication and learning about productive paths toward sustainable bioenergy. Biofuels Bioproducts & Biorefining-Biofpr, 11(2): 243-246 [16] |
| 7 | Di Lucia, L. and Kronsell, A. 2010. The willing, the unwilling and the unable - explaining implementation of the EU Biofuels Directive. Journal of European Public Policy 17(4): 545-563. doi: doi: 10.1080/13501761003673559 [17] |
| 8 | Forest Europe 2015. State of Europe's Forests 2015. FOREST EUROPE Liaison Unit Madrid., p. 1-314. https://www.foresteurope.org/docs/fullsoef2015.pdf [18] |
| 9 | Gunningham, N. 2009. Environment Law, Regulation and Governance: Shifting Architectures. jel 21(2): 179-212 [19] |
| 10 | Kittler, B., Stupak, I. and Smith, C.T. 2020. Assessing the wood sourcing practices of the U.S. industrial wood pellet industry supplying European energy demand. Energy, Sustainability and Society 10 (1) 23 [20] |
| 11 | Koven, A. 2015. Policy Networks and Paradigm Change in Ontario Forest Policy 1988-2014. Faculty of Forestry, University of Toronto [21] |
| 12 | Lehtonen, M. 2007. Environmental policy integration through OECD peer reviews: Integrating the economy with the environment or the environment with the economy? Environmental Politics 16(1): 15-35 [22] |
| 13 | Lindahl, K.B., Sténs, A., Sandstrøm, C., Johansson, J., Lidskog, R., Ranius, T., and Roberge, J.M. 2017. The Swedish forestry model: More of everything? Forest Policy and Economics 77: 44-55 [23] |
| 14 | Mai-Moulin, T., Fritsche, U.R., and Junginger, M. 2019. Charting global position and vision of stakeholders towards sustainable bioenergy. Energy, Sustainability and Society 9(1): 48 [24] |
| 15 | McDermott, C., Cashore, B., and Kanowski, P. 2010. Global Environmental Forest Policies - An international comparison. Earthscan Forest Library, London, Washington DC. pp. 1-372 [25] |
| 16 | McDermott, C.L., Cashore, B., and Kanowski, P. 2009. Setting the bar: an international comparison of public and private forest policy specifications and implications for explaining policy trends. Journal of Integrative Environmental Sciences 6(3): 217-237. doi: doi: 10.1080/19438150903090533 [26] |
| 17 | Mrosek, T., Basille, D., and Schleifenbaum, P. 2006. Field testing of a criteria and indicators system for sustainable forest management at the local level. Case study results concerning the sustainability of the privat forest Haliburton Forest and Wild Life Reserve in Ontario, Canada. 8 ed. pp. 593-609 [27] |
| 18 | Nichiforel, L., Keary, K., Deuffic, P., Weiss, G., Thorsen, B.J., Winkel, G., Avdibegovic, M., Dobsinska, Z., Feliciano, D., Gatto, P., Mifsud, E.G., Hoogstra-Klein, M., Hrib, M., Hujala, T., Jager, L., Jarsky, V., Jodlowski, K., Lawrence, A., Lukmine, D., Malovrh, S.P., Nedeljkovic, J., Nonic, D., Ostoic, S.K., Pukall, K., Rondeux, J., Samara, T., Sarvasova, Z., Scriban, R.E., Silingiene, R., Sinko, M., Stojanovska, M., Stojanovski, V., Stoyanov, N., Teder, M., Vennesland, B., Vilkriste, L., Wilhelmsson, E., Wilkes-Allemann, J., and Bouriaud, L. 2018. How private are Europe's private forests? A comparative property rights analysis. Land Use Policy 76: 535-552 [28] |
| 19 | Purkus, A. 2016. Concepts and Instruments for a Rational Bioenergy Policy - A New Institutional Economics Approach. Springer. pp. 1-418 [29] |
| 20 | Thrän, D., Schaubach, K., Majer, S., and Horschig, T. 2020. Governance of sustainability in the German biogas sector - adaptive management of the Renewable Energy Act between agriculture and the energy sector. Energy, Sustainability and Society 10(1): 3 [30] |
| 21 | Visseren-Hamakers, I.J. and Pattberg, P. 2013. We Can't See the Forest for the Trees The Environmental Impact of Global Forest Certification Is Unknown. Gaia-Ecological Perspectives for Science and Society 22(1): 25-28 [31] |

**Table S5** Working table which was used as a tool to develop the typology presented in Table 11, and the conceptual governance research framework presented in Fig. 9. The literature included in Table S5 was a basis for developing the structure. This table does as such does not represent a comprehensive review and there are not examples in every combination of research focus (R1-R6), comparative approach (A1-A3) and type of research question (Q1-Q3). Type of question combined with research foci has 11 levels in the table and is, in principle, a product of research focus and type of research question, except omitting the “if” question and the research focus “context” (R1). SFM: Sustainable Forest Management.

|  | Type of research question | Type of research question combined with various research foci | Comparative approach | | | Contributions to… | |
| --- | --- | --- | --- | --- | --- | --- | --- |
|  |  |  | Temporal: period of time (A1) | Spatial: multiple policy settings (A2) | Vertical: multiple governance levels (A3) | …answering the overall research question of this paper | …policy recommendations for creating behavioral change |
|  |  |  | Policy setting and governance level held constant | Governance level and point in time held constant | Policy setting and point in time held constant |  |  |
| Policies (R2) | “if”, Q1 | Type 1 questions  Occurrence of changes or differences in policy/ institutional characteristics (R2) | Has a change in policy/institutions occurred over a period of time for a particular regulatory setting at a certain regulatory level? | Do policies/ institutions differ across regulatory settings, for a certain regulatory level at a certain point of time? | Do policies/institutions exist at multiple levels, for a particular regulatory setting at a certain point in time? | Helps to answer the basic question if any further research is relevant. | Usually there will be no doubt among policy makers or other agents about changes to policy/institutional characteristics, whereas principals are not necessarily as well informed. |
|  |  |  |  |  |  |  |  |
|  | “how”, Q2 | Type 2 questions  Patterns of policy /institutional characteristics (comparative analysis) (R2) | When and how has changes in policy/institutional characteristics occurred during a specific period of time, with regard to inputs, outputs and/or throughputs? | How do policies/ institutional characteristics differ across regulatory settings, with regard to inputs, outputs and/or throughputs? | How and to which extent do policiy/institutional characteristics overlap, complement or conflict with one another, with regard to inputs, outputs and/or throughputs? | - Basis for developing and testing hypotheses about causality of the observed changes or differences in policy/institutional characteristics with context (Type 3). | Benchmarking of policy /institutional characteristics may lead to convergence of agent policies and/or principal behaviors, because of improved understanding of general norms or (perceived) better policies in other places with conditions that are seen as be similar to that of the individual agent or principal. |
|  |  |  | *Examples:*   - *Koven (2015) about forest policy in Ontario 1984-2015* - *Al Seadi et al. (2018) about biogas developments in Denmark* - *Gunningham (2009) about environmental law in US and Europe* - *Lindahl et al. (2017) about changes in goals and approach in Swedish forest legislation over 100 years.* - *Abbot and Snidal (2009). Developments in types of agent and agent partnerships occurring in a specific period of time.* | *Examples:*   - *Nichiforel et al. (2018) comparing jurisdictional forest legisilation European scale* - *McDermott et al. (2010) comparing jurisdictional forest legislation at global scale* | *Examples:*   - *Cheung et al. (2019) for the forest sector in Ontario* - *Buliga et al. (2019) for extent of overlap between legal requirements and FSC standards.* | *Examples:*   - *McDermott et al. (2010) indicate hypotheses that differences are due to public or private ownership, developed and developing countries etc. and that some similarities are due to emergence of international norms* | *Examples:*   - *Forest Europe (2015) indicate that the European collaboration on SFM standards as well as benchmarking of forest policies has led to an increasing number of countries that include an increasing number of Forest Europe SFM indicators in their forest legislation.* - *Visseren-Hamaker and Pattberg (2013)* - *Lehtonen (2007) address the use of environmental and economic country reviews and comparisons to enhance environmental policy integration* |
|  | “why”, Q3 | Type 3 questions  Patterns of policy/ institutional characteristics and causality with contextual factors (R2=f(R1)) | Why has this policy/institutional change occurred in a specific period of time? | Why do policies/ institutions differ across regulatory settings? | Why do policies/ institutions overlap, complement or conflict with one another across levels? For which intended or unintended reasons? | - Helps to explain and develop a deeper understanding of which policy/institutional characteristics that occur or result from different contexts. - Helps to create hypotheses about policy/institutional characteristics that lead to real changes or differences (Type 5). - Classified policy/institutional characteristics may be used as indicators of expected real or perceived changes or differences,, and principal’s granted legitimacy and/or trust.* | May point to conditions that indicate political, social and cultural feasibility of different policy/institutional characteristics, and perhaps what needs to change before certain policy/institutional designs can be decided. |
|  |  |  | *Examples:*   - *Koven (2015) – change in forest laws related to changed power relationships* - *Thrän et al. (2019) – change in RES policy related to monitoring of biophysical factors and market development* - *Gunningham (2009) about environmental law in US and Europe* | *Examples:*   - *Kittler et al. (2019) – different wood pellet company policies related to supply chain structure.* |  |  |  |
| Measureable impacts (R3) | “how”, Q2 | Type 4 questions  Patterns of real changes or differences (comparative analysis) (R3) | Have real changes relevant to inputs, outputs and/or throughputs occurred in a specific period of time? | Do real differences relevant to inputs, outputs and/or throughputs occur across regulatory settings? | Have real changes relevant to inputs, outputs and/or throughputs occurred? | - Basis for developing and testing hypotheses about causality of the observed real changes or differences with policy/ institutional characteristics (Type 5). | Benchmarking of real changes or differences for a specific set of factors may motivate agents to develop new policies/institutional designs and/or change principals’ views on these, because of improved understanding of general norms or (perceived) performance under conditions that are seen as similar to that of the individual agent or principal. |
|  |  |  | *Examples:*   - *Al Seadi et al. (2018) and Thrän et al. (2019) – changes in biogas production* | *Examples:*   - *Forest Europe (2015) – comparing forest conditions at European scale* | *Examples:*   - *NGO reports, concerns* |  |  |
|  | “why”, Q3 | Type 5 questions  Patterns of real changes or differences and causality with policy/ institutional characteristics (R3=f(R2)) | How (and why) do real changes relevant to inputs, outputs and/or throughputs link to policy/institutional changes for this period of time? | How (and why) do real differences relevant to inputs, outputs and/or throughputs link to policy/institutional differences? | How (and why) do real changes relevant to inputs, outputs and/or throughputs link to added levels of regulation? Is more being obtained by adding more levels? | - Helps to explain and develop deeper understanding of which policy/ institutional characteristics that are linked to real changes or differences. - Helps to create hypotheses about which real changes that lead to perceived changes (Type 7) - Classified real changes or differences may be used as indicators of expected perceived changes or differences,, and changes to or differences in principal’s granted legitimacy and/or trust.* | May help to create models for prediction of real change, if the basic conditions under which the causality was proved do not change in a way that leaves the causality no longer valid. This includes economic policy analyzes to identify the first, second or third most efficient and effective policy among a set of alternatives. |
|  |  |  | *Examples:*   - *Al Seadi et al. (2018) – Development of the biogas sector in Denmark related to policy* - *Thrän et al. (2019) –Development of biogas sector in Germany related to policy* | *Examples:*   - *Di Lucia and Kronsell (2010). - Effectiveness in reaching biofuel targets depending on EU Member State policy and institutional capacity variables* | *Examples:*   - *Mrosek et al. (2006) –effectiveness of forest certification measured by corrective action requests (CAR***)* |  | *Examples:*   - *Purkus (2016) – suggesting instruments for a rational bioenergy policy* |
|  | “how”, Q2 | Type 6 questions  Patterns of perceived of changes or differences (comparative analysis)  (R4) | Have perceptions of changes relevant to inputs, outputs and/or throughputs occurred in a specific period of time?  Are there differences among specific groups? | Do perceptions relevant to inputs, outputs and/or throughputs differ across regulatory settings  Are there differences among specific groups? | Are perceptions of changes relevant to inputs, outputs and/or throughputs occurring before?  Are there differences among specific groups? | - Basis for developing and testing hypotheses about causality between perceived changes or differences and policy/ institutional characteristics, or real changes or differences (Type 7). | Benchmarking may motivate agents to develop new policies or strategies to change principals’ views on existing policies/institution or their effectiveness, based on researchers or own hypotheses about how perceived changes are linked to real changes or changes in policies or institutions |
| Perceived impacts (R4) |  |  |  |  | *Examples:*   - *Mai-Moulin et al. (2019) – differences in perceptions among stakeholder groups* - *Dale et al. Interactive poster – perceptions of environmental risk* |  |  |
|  | “why”, Q3 | Type 7 questions  Patterns of perceived changes or differences and causality with policy/  institutional characteristics or real changes or differences  (R4=f(R2), R4=R(R2)) | How (and why) do perceived changes relevant to inputs, outputs and/or throughputs link to policy/institutional changes or to real changes for a specific period of time? | How (and why) do differences in perceptions relevant to inputs, outputs and/or throughputs link to policy/institutional differences or real changes? | How (and why) do perceived changes relevant to inputs, outputs and/or throughputs link to added levels of policies/institutions or to real changes? | - Helps to explain and develop deeper understanding of how perceived changes are linked to policy/ institutional characteristics, or real changes or differences. - Helps to create hypotheses about which perceived changes or differences that are linked to which changes or differences in legitimacy (Type 9) - Classified perceived changes or differences may be used as indicators of expected changes to or differences in principal’s granted legitimacy and/or trust.* | May prompt agents to elaborate or improve their communication strategies about their polices and their effectiveness, to change principal’s perceptions about these matters, based on what is known about possible gaps between real and perceived changes, and ultimately achieve higher levels of legitimacy and principals’ granting of trust, assuming that this will change with a change in perceptions. |
|  |  |  |  |  |  |  |  |
| Legitimacy (R5) | “how”, Q2 | Type 8 question  Changes or differences in legitimacy (comparative analysis) (R5) | Have changes in granting of input, output and/or throughput legitimacy occurred in a specific period of time?  Are there differences among specific groups? | Do granted input, output and/or throughput legitimacy differ across regulatory settings?  Are there differences among specific groups? | Have changes in granting of input, output and/or throughput legitimacy occurred?  Are there differences among specific groups? | - Helps to develop hypotheses about causality between the legitimacy changes and changes to or differences in policy/ institutional characteristics, or real or perceived changes (Type 9). | Benchmarking may motivate or push agents to develop new policies and/or change principals’ views on policies or institutions, because of improved understanding of general norms or (perceived) levels of legitimacy under conditions that are seen as similar to that of the individual agent or principal. |
|  |  |  |  |  |  |  |  |
|  | “why”, Q3 | Type 9 questions  Changes or differences in legitimacy and causality with policy/ institutional characteristics, real or perceived changes  (R5=f(R2), R5=f(R3), R5=f(R4)) | How (and why) do changes ing granting of input, output and/or throughput legitimacy link to policy/institutional changes or real or perceived changes in a specific period of time?  Are there differences among specific groups? | How (and why) do differences in input, output and/or throughput legitimacy link to policy/instructional differences or real or perceived differences?  Are there differences among specific groups? | How (and why) do changes in input, output and/or throughput legitimacy link to added levels of policies/institutions or real or perceived changes?  Are there differences among specific groups? | - Helps to explain and develop deeper understanding of how principals’ granting of legitimacy is linked to policy/ institutional characteristics, or real or perceived changes or differences. - Helps to create hypotheses about how changes to or differences in principal’s granting of legitimacy is linked to changes to or differences in granting of trust (Type 11) - Classified changes to or differences in principal’s granted legitimacy may be used as indicators of expected changes to or differences in principal’s granted trust.* | May prompt agents to elaborate or improve their strategies to increase principals’ granting of legitimacy, based on what is known about its dependency on the effectiveness of polices/institutions in creating real or perceived change in factors related to policy/institutional inputs, outputs, or throughputs. |
|  |  |  |  | *Examples:*   - *Bennet et al. (2019) – local support for conservation was linked to perception of good governance, environmental effectiveness and social impact* |  |  |  |
| Trust (R6) | “how”, Q2 | Type 10 questions  Patterns of changes or differences in trust (R6) | Have changes in granting of trust occurred in a specific period of time?  Are there differences among specific groups? | Do granting of trust differ across regulatory settings?  Are there differences among specific groups? | Have changes in granting of trust occurred?  Are there differences among specific groups? | - Helps to develop hypotheses about causality between changes to or differences in trust and changes to or differences in with policy/ institutional characteristics, real or perceived changes or differences, or with changes or differences in legitimacy (Type 11) * | Benchmarking may motivate or push agents to develop new policies and/or change principals’ views on policies or institutions, because of improved understanding of general norms or (perceived) levels of trust under conditions that are seen as similar to that of the individual agent or principal. |
|  |  |  |  |  |  |  |  |
|  | “why”, Q3 | Type 11 questions  Changes or differences in trust and causality with policy/ institutional characteristics, real or perceived changes or differences, or with changes or differences in legitimacy**  (R6=f(R2), R6=f(R3), R6=f(R4),  R6=f(R5)) | How (and why) do changes in trust link to policy/ institutional changes or real or perceived changes, or with changes in legitimacy?  Are there differences among specific groups? | How (and why) do differences in trust link to policy/institutional differences or real or perceived differences, with with differences in legitimacy?  Are there differences among specific groups? | How (and why) do changes in trust link to added levels of regulatory activity or real changes, assuming that real changes occur as a consequence of policy changes?  Are there differences among specific groups? | - Helps to explain and develop deeper understanding of how principals’ granting of trust is linked to policy/ institutional characteristics, real or perceived changes or differences, and principal’s granting of legitimacy. | May prompt agents to elaborate or improve their strategies to increase principals’ granting of trust, based on what is known about its dependency on legitimacy, or effectiveness of polices/institutions in creating real or perceived change in factors related policy/institutional to inputs, outputs, or throughputs. |
|  |  |  |  |  |  |  |  |
| *In the absence of verified knowledge.  **To the extent that trust and legitimacy are not overlapping, cf. Fig. 3a.  ***See also Table 6. | | | | | | | |

**Reference List**

1. World Bank (2020) Worldwide Governance Indicators. <http://info.worldbank.org/governance/wgi/>. Accessed 15 Aug 2020

2. UNESCAP (2009) What is Good Governance? United Nations Economic and Social Commission for Asia and the Pacific, 3 pp. <https://www.unescap.org/resources/what-good-governance>. Accessed 15 Aug 2020

3. Council of Europe (2008) 12 Principles of Good Democratic Governance. European Label of Governance Excellence (ELoGE), Council of Europe. Enshrined in the Strategy on Innovation and Good Governance at local level, endorsed by a decision of the Committee of Ministers of the Council of Europe in 2008, 8 pp. <https://www.coe.int/en/web/good-governance/12-principles>. Accessed 15 Aug 2020

4. ISEAL (2013) Principles for Credible and Effective Sustainability Standards Systems. ISEAL Credibility Principles. ISEAL Alliance, 18 pp. <https://www.isealalliance.org/credible-sustainability-standards/iseal-credibility-principles>. Accessed 15 Aug 2020

5. Rothstein B (1992) The Quality of Government. Corruption, Social Trust, and in Inequality in International Perspective. The University of Chicago Press, Chicago and London, 285 pp.

6. Davidovic D, and Harring N (2019) Does Quality of Government and Trust explain the Cross-national Variation in Public Support for Climate Policy? The Quality of Government Institute, Department of Political Science, University of Gothenburg, Gothenburg, Sweden. QoG Working Paper Series 2019(2), 42 pp. <https://qog.pol.gu.se/digitalAssets/1719/1719925_2019_2_davidovic_harring.pdf>. Accessed 15 Aug 2020

7. Göhler D, Cashore B, Blom B (2014) Forest Governance and Sustainable Rural Development. In Pretzsch J, Darr D, Uibrig H, Auch E (eds) For Rural Dev. Springer, Berlin, Heidelberg, pp. 333-373. <https://doi.org/10.1007/978-3-642-41404-6_12>

8. Bennett NJ, Satterfield T (2018) Environmental governance: A practical framework to guide design, evaluation, and analysis. Conserv Lett 11(6):e12600. <https://doi.org/10.1111/conl.12600>

9. Schmidt V, Wood M (2019) Conceptualizing throughput legitimacy: Procedural mechanisms of accountability, transparency, inclusiveness and openness in EU governance. Public Adm 97(4):727-740. <https://doi.org/10.1111/padm.12615>

10. Stupak I, Smith CT (2019) Acceptance and feasibility of FMU-level and risk-based certification for ensuring sustainability of wood pellet supply chains - conceptual overview. Presentation given in the workshop "Adequacy of spatial databases for conducting risk assessments of sustainable wood sourcing practices of the U.S. industrial wood pellet industry supplying European energy demand", Athens, Georgia, U.S.A, 1-3 May 2019.

11. Abbott KW, Snidal D (2009) The Governance Triangle: Regulatory Standards Institutions and the Shadow of the State. In: Mattli W, Woods N (eds) The Politics of Global Regulation. Princeton University Press, pp. 44-88.

12. Al-Seadi T, Stupak I, Smith CT (2018) Governance of environmental sustainability of manure-based centralised biogas production in Denmark. Murphy JD (ed) IEA Bioenergy Task 37, Report 2018:7, 32 pp. <https://www.ieabioenergy.com/publications/governance-of-environmental-sustainability-of-manure-based-centralised-biogas-production-in-denmark/>. Accessed 15 Aug 2020.

13. Bennett NJ, Di Franco A, Caló A, Nethery E, Niccolini F, Milazzo M, Guidetti P (2019) Local support for conservation is associated with perceptions of good governance, social impacts, and ecological effectiveness. Conserv Lett 12(4):e12640. <https://doi.org/10.1111/conl.12640>

14. Buliga B, Nichiforel L (2019) Voluntary forest certification vs. stringent legal frameworks: Romania as a case study. J Cleaner Prod 207:329-342. <https://doi.org/10.1016/j.jclepro.2018.10.021>

15. Cheung Q, Smith CT, Stupak I (2019) Governance of sustainable forest management and bioenergy feedstock harvesting in Ontario, Canada. IEA Bioenergy Task 43: TR2019:04, 62 pp. <http://task43.ieabioenergy.com/publications/governance-of-sustainable-forest-management-and-bioenergy-feedstock-harvesting-in-ontario-canada/>. Accessed 15 Aug 2020

16. Dale VH, Kline KL (2017) Interactive posters: A valuable means of enhancing communication and learning about productive paths toward sustainable bioenergy. Biofuels Bioprod Biorefin 11(2):243-246. <https://doi.org/10.1002/bbb.1753>

17. Di Lucia L, Kronsell A (2010) The willing, the unwilling and the unable − explaining implementation of the EU Biofuels Directive. J Eur Public Policy 17(4):545-563. <https://doi.org/10.1080/13501761003673559>

18. Forest Europe (2015) State of Europe's Forests 2015. Forest Europe Liaison Unit, Madrid, 134 pp. <https://www.foresteurope.org/docs/fullsoef2015.pdf>. Accessed 15 Aug 2020.

19. Gunningham N (2009) Environment Law, Regulation and Governance: Shifting Architectures. J Environ Law 21(2):179-212. <https://doi.org/10.1093/jel/eqp011>

20. Kittler B, Stupak I, Smith CT (2020) Assessing the wood sourcing practices of the U.S. industrial wood pellet industry supplying European energy demand. Energy Sustain Soc 10(1):23. <https://doi.org/10.1186/s13705-020-00255-4>

21. Koven A (2015) Policy Networks and Paradigm Change in Ontario Forest Policy 1988-2014. Ph.D thesis, Faculty of Forestry, University of Toronto, Toronto, 281 pp. <https://tspace.library.utoronto.ca/handle/1807/71038>. Accessed 15 Aug 2020

22. Lehtonen M (2007) Environmental policy integration through OECD peer reviews: Integrating the economy with the environment or the environment with the economy? Environ Polit 16(1):15-35. <https://doi.org/10.1080/09644010601073432>

23. Lindahl KB, Sténs A, Sandstrøm C, Johansson J, Lidskog R, Ranius T, Roberge JM (2017) The Swedish forestry model: More of everything? For Policy Econ 77:44-55. <https://doi.org/10.1016/j.forpol.2015.10.012>

24. Mai-Moulin T, Fritsche UR, Junginger M (2019) Charting global position and vision of stakeholders towards sustainable bioenergy. Energy Sustain Soc 9(1):48. <https://doi.org/10.1186/s13705-019-0225-0>

25. McDermott CL, Cashore B, Kanowski P (2010) Global Environmental Forest Policies - An international comparison. Earthscan Forest Library, London, Washington DC, 372 pp.

26. McDermott CL, Cashore B, Kanowski P (2009) Setting the bar: an international comparison of public and private forest policy specifications and implications for explaining policy trends. Journal of Integrative Environ Sci 6(3):217-237. <https://doi.org/10.1080/19438150903090533>

27. Mrosek T, Balsillie D, Schleifenbaum P (2006) Field testing of a criteria and indicators system for sustainable forest management at the local level. Case study results concerning the sustainability of the private forest Haliburton Forest and Wild Life Reserve in Ontario, Canada. For Policy Econ 8(6):593-609. <https://doi.org/10.1016/j.forpol.2004.11.002>

28. Nichiforel L, Keary K, Deuffic P, Weiss G, Thorsen BJ, Winkel G, Avdibegovic M, Dobsinska Z, Feliciano D, Gatto P, Mifsud EG, Hoogstra-Klein M, Hrib M, Hujala T, Jager L, Jarsky V, Jodlowski K, Lawrence A, Lukmine D, Malovrh SP, Nedeljkovic J, Nonic D, Ostoic SK, Pukall K, Rondeux J, Samara T, Sarvasova Z, Scriban RE, Silingiene R, Sinko M, Stojanovska M, Stojanovski V, Stoyanov N, Teder M, Vennesland B, Vilkriste L, Wilhelmsson E, Wilkes-Allemann J, Bouriaud L (2018) How private are Europe's private forests? A comparative property rights analysis. Land Use Policy 76:535-552. <https://doi.org/10.1016/j.landusepol.2018.02.034>

29. Purkus A (2016) Concepts and Instruments for a Rational Bioenergy Policy - A New Institutional Economics Approach. Springer, Lecture Notes in Energy 55, 418 pp. <https://doi.org/10.1007/978-3-319-31135-7>

30. Thrän D, Schaubach K, Majer S, Horschig T (2020) Governance of sustainability in the German biogas sector − adaptive management of the Renewable Energy Act between agriculture and the energy sector. Energy Sustain Soc 10(1):3. <https://doi.org/10.1186/s13705-019-0227-y>

31. Visseren-Hamakers IJ, Pattberg P (2013) We Can't See the Forest for the Trees The Environmental Impact of Global Forest Certification Is Unknown. Gaia - Ecol Perspect Sci Soc 22(1):25-28. <https://doi.org/10.14512/gaia.22.1.8>
